# Combining High-Resolution Mass Spectrometry and Chemiluminescence Analysis to Characterize the Composition and Fate of Total *N*-Nitrosamines in Wastewater Treatment Plants

**DOI:** 10.1021/acs.est.4c06555

**Published:** 2024-09-10

**Authors:** Changcheng Pu, Benjamin R. Cavarra, Teng Zeng

**Affiliations:** Department of Civil and Environmental Engineering, Syracuse University, 151 Link Hall, Syracuse, New York 13244, United States

**Keywords:** *N*-nitroso compounds, TONO, high-resolution mass spectrometry, nontargeted analysis, chemiluminescence, WWTPs

## Abstract

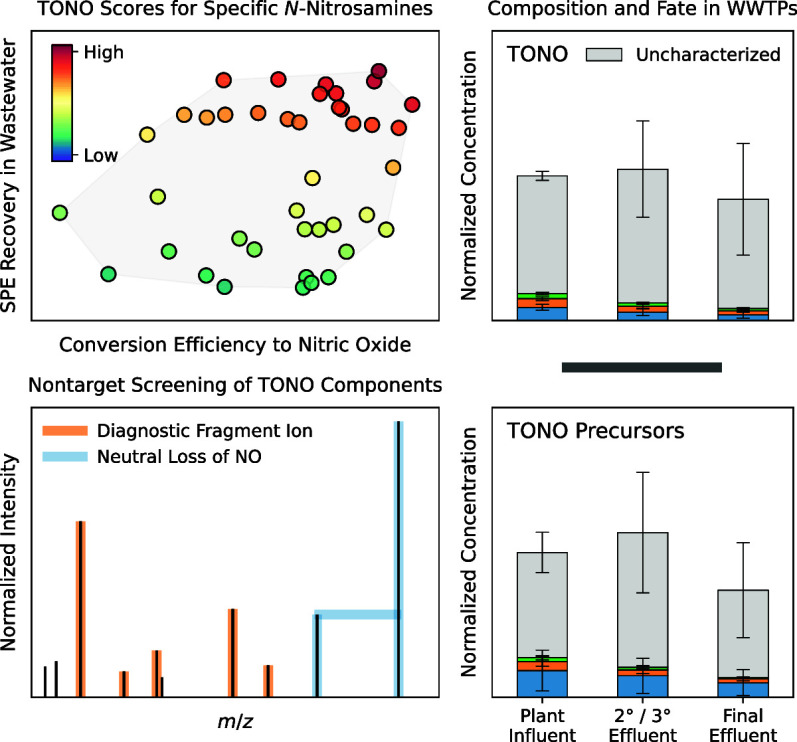

Monitoring the prevalence and persistence of *N*-nitrosamines and their precursors in wastewater treatment plants (WWTPs) and effluent-receiving aquatic compartments is a priority for utilities practicing wastewater recycling or exploiting wastewater-impacted source waters. In this work, we developed an analytical framework that combines liquid chromatography-high-resolution mass spectrometry (LC-HRMS) with acidic triiodide-chemiluminescence analysis to characterize the composition and fate of total *N*-nitrosamines (TONO) and their precursors along the treatment trains of eight WWTPs in New York. Through the parallel application of LC-HRMS and chemiluminescence methods, the TONO scores for 41 *N*-nitrosamines containing structurally diverse substituents on their amine nitrogen were derived based on their solid-phase extraction recoveries and conversion efficiencies to nitric oxide. Correcting the compositional analysis of TONO using the TONO scores of target *N*-nitrosamines refined the assessment of the reduction or accumulation of TONO and their precursors across treatment steps in WWTPs. Nontargeted analysis prioritized seven additional *N*-nitrosamines for confirmation by reference standards, including three previously uncharacterized species: *N*-nitroso-*tert*-butylphenylamine, *N*-nitroso-2-pyrrolidinmethanol, and *N*-nitrosodesloratadine, although they only served as minor components of TONO. Overall, our study establishes an adaptable methodological framework for advancing the quantitative and qualitative analysis of specific and unknown components of TONO across water treatment and reuse scenarios.

## Abstract 2

Targeted analysis and nontarget screening based on LC-HRMS are combined with acidic triiodide-chemiluminescence measurements to characterize the composition and fate of total *N*-nitrosamines in wastewater treatment plants.

## Introduction

*N*-Nitrosamines are compounds characterized by a nitroso functional group attached to a nitrogen atom and can occur as impurities in consumer and industrial products^1^ or form as byproducts during oxidative water treatment.^2^ Since the discovery of *N*-nitrosodimethylamine (NDMA) as a drinking water contaminant,^3^ mitigating public exposure to *N*-nitrosamines via potable water consumption has attracted significant scientific and regulatory attention due to their mutagenic potential and carcinogenic potency.^4−7^ For example, dialkyl, alkylaryl, and cycloalkyl *N*-nitrosamines may undergo metabolic activation via α-hydroxylation catalyzed by cytochrome P450 enzymes to form intermediates that further decompose into reactive diazonium ions capable of alkylating cellular biomacromolecules such as DNA,^8^ although the potency of *N*-nitrosamines varies by several orders of magnitude^9^ depending on their structural features (e.g., the degree of steric hindrance at the α-carbon, electron-withdrawing potential at the β-carbon, or the size and nature of ring moieties).^10,11^ Over the past two decades, the occurrence, formation, and control of NDMA and five other *N*-nitrosamines on the U.S. Environmental Protection Agency (EPA)’s Contaminant Candidate List have been extensively studied in the broad context of drinking water and wastewater treatment^12−17^ as well as in water reuse scenarios.^18−26^ However, NDMA and the few commonly detected *N*-nitrosamines (e.g., *N*-nitrosomorpholine (NMOR)) constitute only a fraction of total *N*-nitrosamines (TONO).^27−31^ Concurrent measurements of specific *N*-nitrosamines and TONO in domestic sewage, treated effluents, recycled wastewater, and source waters have also provided evidence that organic nitrogen species, ranging from secondary and tertiary amines to polymeric and protein-like substances, collectively serve as precursors to uncharacterized TONO.^27−31^ Together, previous research has established baseline levels of TONO in a range of environmental water samples and identified potential sources of their precursors in natural and engineered aquatic systems.

To date, studies reporting the occurrence of TONO in wastewater matrices have primarily applied gas chromatography-tandem mass spectrometry (i.e., GC–MS/MS^32^) in parallel with chemiluminescence assays (e.g., based on the detection of nitric oxide released from samples via chemical or photolytic denitrosation) to analyze specific and total *N*-nitrosamines, respectively.^27,28,30,31^ With this approach, the comparability of GC–MS/MS and chemiluminescence data was limited by inconsistencies in sample preconcentration protocols and variability in denitrosation efficiencies among structurally diverse *N*-nitrosamines.^27,28,31,33^ Complementing targeted analysis by GC–MS/MS, more recent work has developed suspect and nontarget screening workflows enabled by liquid chromatography-high-resolution mass spectrometry (LC-HRMS) to identify precursors to NDMA and a few *N*-nitrosamines of current interest through diagnostic fragmentation pattern filtering^22,34,35^ or stable isotope labeling.^36^ Comparatively limited efforts have, however, investigated uncharacterized components of TONO and their precursors in wastewater through the parallel application of LC-HRMS and chemiluminescence methods.^31^

Considering the need to refine the compositional analysis of TONO, our primary objective of this work was (i) to develop an analytical framework that combines LC-HRMS and chemiluminescence analyses for scoring the significance of specific *N*-nitrosamines as TONO components based on their solid-phase extraction (SPE) recoveries and conversion efficiencies to nitric oxide upon denitrosation. Two additional objectives were (ii) to examine the composition and fate of TONO, as well as their chloramine-reactive precursors, in eight full-scale wastewater treatment plants (WWTPs) in New York and (iii) to demonstrate a nontarget screening approach for prioritizing and identifying uncharacterized *N*-nitrosamines in wastewater.

## Materials and Methods

Chemical sources and reagent preparation are described in Text S1. *N*-Nitrosamine reference standards and isotope-labeled internal standards (ILIS) are listed in Table S1.

### Sample Collection

Wastewater samples (i.e., 24-h flow-proportional composite) were collected on midweek days by automatic samplers installed along the treatment trains of eight WWTPs in New York (Table S2). WWTPs A-F serve urban residential and commercial communities, whereas WWTPs G and H serve rural communities but also receive effluents discharged by a pharmaceutical manufacturing facility and a regional medical center, respectively. WWTPs A-H utilized activated sludge or attached growth systems for biochemical oxygen demand (BOD) removal and/or nitrification, aluminum sulfate, or ferric chloride for phosphorus precipitation, and sodium hypochlorite (NaOCl) or low-pressure UV systems for disinfection. WWTPs A-F were sampled in spring and fall, while WWTPs G and H were sampled in summer. Within 24 h of collection, samples were transported on ice to Syracuse University, filtered sequentially through precombusted 2.7-μm (to minimize clogging by solids) and 0.7-μm glass fiber filters (to minimize leaching of *N*-nitrosamine precursors^13,15^), and stored at −20 °C until analysis. Note that filtration precluded the analysis of particle-associated *N*-nitrosamines and their precursors.^13,37^ Optical properties (e.g., fluorescence intensities of organic matter components; Figures S1−S4) of filtered samples were measured by a HORIBA Scientific Aqualog spectrofluorometer. General operational (e.g., flow rates) and wastewater quality parameters (e.g., BOD_5_, total Kjeldahl nitrogen, and ammonia nitrogen; Table S3) were provided by the WWTP operators.

### Sample Treatment and Preconcentration

Each sample (or field blank) was split into eight 250 mL aliquots for duplicate LC-HRMS and chemiluminescence analyses. Four of these aliquots were treated with 2 mM of freshly prepared preformed monochloramine (NH_2_Cl) for 10 d in the dark at 21 ± 1 °C following the formation potential test protocol (initially developed to promote the conversion of chloramine-reactive precursors to *N*-nitrosamines),^13^ followed by 1.1 mM of l-ascorbic acid to quench chloramine residuals and 20 mM of sulfamic acid to eliminate nitrite, respectively.^38,39^ Four remaining aliquots were directly treated with l-ascorbic and sulfamic acids to quantify the background levels of *N*-nitrosamines. Sample aliquots were then passed through surface-modified polymeric styrene-divinylbenzene copolymer cartridges (Strata-X 33 μm, 200 mg/6 mL; Phenomenex) mounted on top of coconut-shell activated carbon cartridges (Enviro-Clean 521, 2000 mg/15 mL; United Chemical Technologies).^33,40^ Sample aliquots intended for LC-HRMS analysis were spiked with a mixture of eight ILIS (100 ng/L each) before SPE. SPE extracts were concentrated by rotary evaporation and reconstituted with methanol:water to 1 mL. Selected sample aliquots were also shipped to the U.S. Geological Survey (USGS) National Water Quality Laboratory for targeted analysis of pharmaceuticals and other wastewater-derived substances using liquid chromatography-tandem mass spectrometry (LC–MS/MS).^41^

### Sample Analysis by LC-HRMS

Half of the SPE extracts were analyzed for specific *N*-nitrosamines by a Thermo Fisher Scientific TriPlus RSH autosampler and liquid handling system hyphenated with a Vanquish Horizon ultrahigh-performance liquid chromatograph and an Orbitrap Exploris 240 quadrupole-Orbitrap mass spectrometer. Typically, 20 μL of SPE extracts were injected onto a Poroshell 120 phenyl-hexyl column (150 × 2.1 mm, 3 μm; preceded with a 10 × 2.1 mm guard cartridge) running a gradient of LC–MS grade water and methanol (both acidified with 0.1% v/v formic acid) as the mobile phases at a flow rate of 200 μL/min and a column temperature of 35 °C. High-resolution accurate mass screening was conducted in positive and negative electrospray ionization modes. Full scan mass spectra were acquired from 50 to 1000 Da with a mass resolution of 60,000 at *m*/*z* 200 using fluoranthene for internal mass calibration. Full scan triggered data-dependent tandem mass (dd-MS2) spectra were acquired for precursor ions for *N*-nitrosamines targeted by EPA Method 521,^32^ NMOR, and *N*-nitrosodiphenylamine, or for the five most intense precursor ions, with a mass resolution of 15,000 at *m*/*z* 200 using higher energy collisional dissociation. Calibration standards, method blanks, and quality control samples were preconcentrated by SPE and run with each analytical sequence. Complete details of the LC-HRMS instrument settings and method parameters are provided in Tables S4 and S5.

Quantification of target *N*-nitrosamines was performed in *TraceFinder 5.1 SP3* (Thermo Fisher Scientific) using calibration curves generated by the nonweighted linear regression. *N*-Nitrosamines were confirmed by verifying their retention times and dd-MS2 spectra against those of the reference standards. Concentrations of six target *N*-nitrosamines (i.e., NDMA, NMOR, *N*-nitrosomethylethylamine (NMEA), *N*-nitrosodiethylamine (NDEA), *N*-nitrosodipropylamine (NDPA), and *N*-nitrosopiperidine (NPIP)) detected in samples were quantified with reference to ILIS. Nontargeted data were processed in *Compound Discoverer 3.3 SP2* (Thermo Fisher Scientific) using a node-based workflow consisting of spectra selection, retention time alignment, peak componentization, gap filling, background subtraction, batch effect correction, *N*-nitroso compound list search, NO loss search, and a postprocessing *R* script. With this workflow, mass spectral features containing at least two nitrogen atoms and one oxygen atom were prioritized for inspection if they met all of the following criteria: a positive log2-fold change in peak area ratios between chloraminated and raw samples, single or multiple matches in an in-house *N*-nitroso compound list (Table S7) with a mass tolerance of 5 ppm, and the presence of diagnostic fragment ions and/or NO loss. Complete details of the *Compound Discoverer* node settings are provided in Table S6. Mass spectral features of interest were further evaluated using *FreeStyle 1.8 SP2* (Thermo Fisher Scientific) and, when possible, confirmed by reference standards (i.e., confidence level 1^42^). Quantification of nontarget *N*-nitrosamines confirmed at level 1 was also performed in *TraceFinder*.

### Sample Analysis by Chemiluminescence Detection

Half of the SPE extracts were analyzed in parallel for TONO based on chemiluminescence detection of nitric oxide liberated from the denitrosation of *N*-nitrosamines via acidic triiodide treatment (abbreviated as “HI_3_–CL”).^33^ Typically, 100 μL of SPE extracts were injected into a custom-made water-jacketed purge vessel (maintained at 80 °C and filled with 40 mL of glacial acetic acid plus 4 mL of freshly prepared triiodide solution) surmounted by a coil condenser (maintained at 5 °C). Throughout each analytical sequence, nitric oxide was continuously purged by N_2_ into a CLD 88Yet NO/NO_*x*_ analyzer (EcoPhysics). Chemiluminescence signals resulting from the relaxation of excited state nitrogen dioxide (formed by nitric oxide oxidation by ozone within the analyzer) to the ground state were monitored by a *Microsoft Excel* macro program. Calibration standards and method blanks were analyzed with each sample sequence. Quantification of TONO was performed in *ACD/Labs Spectrus Processor 2021.2.2* by calibrating the peak areas of SPE extracts against those of calibration standards.

### Data Analysis

For each sample, the concentration of TONO measured by HI_3_–CL was partitioned into specific and uncharacterized components. Monte Carlo simulations were performed using *Colab Pro* (Google) to propagate the uncertainty associated with the summed contribution of target *N*-nitrosamines (i.e., NDMA, NMOR, NMEA, NDEA, NDPA, and NPIP) to TONO, which was calculated by correcting their molar concentrations measured by LC-HRMS with their respective TONO scores (i.e., operationally defined as the product of their SPE recoveries in pooled wastewater samples and conversion efficiencies to nitric oxide upon acidic triiodide treatment). Multiple comparisons tests, Spearman’s correlation analysis, and principal component analysis were also performed using *Colab Pro*.

## Results and Discussion

### TONO Scores for Specific *N*-Nitrosamines

**Figure 1:**
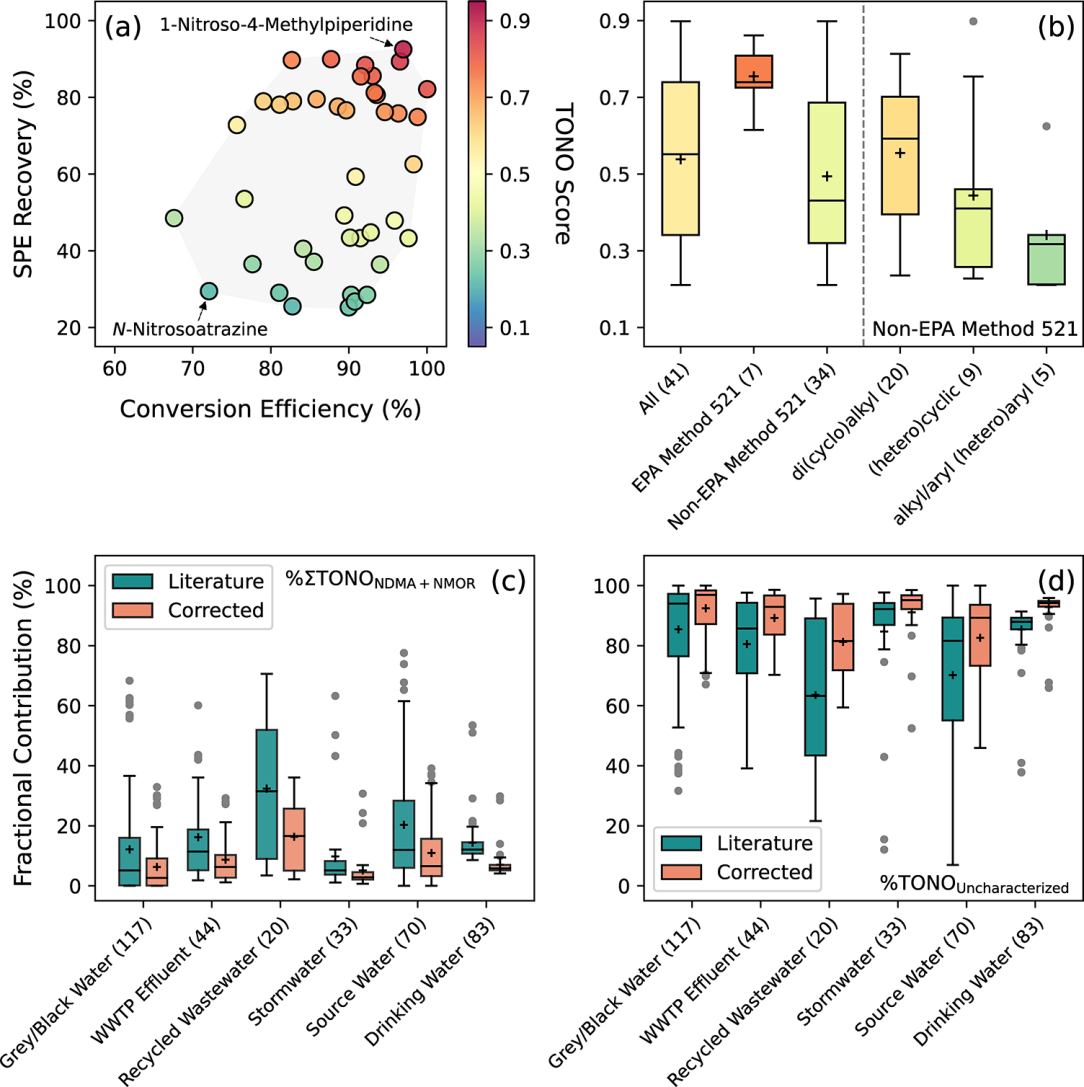
Calculation of TONO scores and their example application in TONO compositional analysis: (a) TONO scores for *N*-nitrosamines (*n* = 41) with di(cyclo)alkyl, (hetero)cyclic, and alkyl/aryl (hetero)aryl substituents. The color bar measures TONO scores calculated based on the SPE recoveries of *N*-nitrosamines in pooled wastewater samples and their conversion efficiencies to nitric oxide upon acidic triiodide treatment. (b) Comparison of TONO scores for different categories of *N*-nitrosamines. (c) Comparison of the summed fractional contribution of NDMA and NMOR to TONO in environmental water samples before and after applying corrections with TONO scores. (d) Comparison of the fractional contribution of uncharacterized *N*-nitrosamines to TONO in environmental water samples before and after applying corrections with TONO scores. Numbers in parentheses represent the number of data points from the literature. Each box extends from the 25th–75th percentiles. The whiskers extend down to the 25th percentile minus 1.5 times the interquartile range and up to the 75th percentile plus 1.5 times the interquartile range. The centerline and “+” sign mark the median and mean, respectively. The gray circles represent the outliers.

Concurrent GC–MS/MS and chemiluminescence measurements applied by previous studies at best provided a semiquantitative assessment of the contributions of specific *N*-nitrosamines to TONO. For GC–MS/MS, activated carbon-based SPE cartridges were eluted with dichloromethane;^32,43^ however, for chemiluminescence analysis, cartridges were eluted with either methanol alone^30^ or dichloromethane followed by solvent exchange into methanol,^27,28^ leading to discrepancies in the absolute recoveries of *N*-nitrosamines. Taking NDMA and NMOR as examples, the recoveries derived from EPA Method 521 (e.g., 90 ± 5%) for GC–MS/MS were higher than those obtained for chemiluminescence analysis (e.g., 58 ± 18%),^27,28^ so their fractional contributions to TONO would have been overestimated without accounting for such discrepancies. Complicating the interpretation of TONO data further, the conversion efficiencies of *N*-nitrosamines to nitric oxide were not necessarily equal to that of NDMA.^33^ To more accurately quantify the significance of specific *N*-nitrosamines as TONO components, it is also necessary to correct for variability in their conversion efficiencies to nitric oxide when calibrating chemiluminescence signals with NDMA. To this end, the TONO scores for 41 *N*-nitrosamines were calculated by multiplying their SPE recoveries in pooled wastewater samples by their conversion efficiencies to nitric oxide. For these *N*-nitrosamines, the SPE recoveries ranged from 25 ± 3% for *N*-nitrosoanatabine to 93 ± 3% for 1-nitroso-4-methylpiperidine with a median of 63% (similar to the ranges reported by prior work^39,40,43^), and the conversion efficiencies to nitric oxide ranged from 68 ± 12% for *N*-nitrosohydroxyproline to 99 ± 8% for NMEA with a median of 90% relative to NDMA (Table S8).^33^ Only half of these *N*-nitrosamines had a TONO score above 0.6 under our experimental conditions, whereas the TONO scores for the remaining *N*-nitrosamines with low SPE recoveries (i.e., <60%) were consistently low (e.g., 0.21 ± 0.03 for *N*-nitrosoatrazine) regardless of their conversion efficiencies (Figure 1a). On average, the TONO scores for seven *N*-nitrosamines (i.e., 0.75 ± 0.09; Figure 1b) targeted by EPA Method 521 overlapped with the ranges for non-EPA Method 521 *N*-nitrosamines with di(cyclo)alkyl substituents (i.e., 0.55 ± 0.19; *n* = 20) on the amine nitrogen (Tukey’s multiple comparisons test *p* = 0.1611) but were higher than those for *N*-nitrosamines with (hetero)cyclic (i.e., 0.44 ± 0.23; *n* = 9) and alkyl/aryl (hetero)aryl (i.e., 0.34 ± 0.16; *n* = 5) substituents (Tukey’s multiple comparisons test *p* = 0.0061–0.0227).

To assess the relevance of TONO scores in estimating the fractional contributions of specific *N*-nitrosamines to TONO, 367 sets of *N*-nitrosamine data for six categories of environmental water samples were collected from the literature for reanalysis.^27−29,44^ Without considering TONO scores, the mean fractional contributions of NDMA and NMOR to TONO summed to 12% for gray and black waters from residential households (*n* = 117),^28^ 16% for secondary or tertiary effluents from WWTPs (*n* = 44),^27,29^ 32% for recycled wastewater from advanced treatment trains (*n* = 20),^27,29^ 10% for urban and agricultural stormwater runoff (*n* = 33),^29^ 20% for pristine and bloom-impacted source waters (*n* = 70),^29^ and 14% for non-nitrifying and nitrifying drinking water (*n* = 83),^44^ respectively. Taking into account the TONO scores for NDMA and NMOR reduced their summed fractional contributions to TONO by 48 ± 2% to 6%, 9%, 16%, 5%, 11%, and 7%, respectively (Figure 1c). Consequently, the fractional contribution of uncharacterized *N*-nitrosamines to TONO increased from 64–85% to 81–93% for the corresponding samples (Figure 1d). Together, these analyses supported the necessity of incorporating TONO scores as correction factors for the concentrations of specific *N*-nitrosamines to refine the compositional analysis of TONO.

### Fate of TONO in WWTPs

Overall, TONO ranged from 1.4 ± 0.1 to 17 ± 1.8 nM with a median of 4.5 nM in samples from WWTPs A-H (Figures S5–S7) and overlapped with the concentrations (i.e., 1.2–13 nM) in WWTP samples from other U.S. regions (e.g., California and Arkansas).^27,29,30^ Only three target *N*-nitrosamines (i.e., NDMA, NMOR, and NMEA) were detected at quantifiable levels (i.e., 0.2–1.2 nM) in samples from WWTPs A-H. Consistent with earlier findings,^28,29^ the fractional contribution of uncharacterized *N*-nitrosamines to TONO (i.e., 88 ± 5%) dominated over the summed contribution of NDMA, NMOR, and NMEA, with the highest percentage (i.e., 96 ± 3%) measured for return activated sludge (RAS) supernatants.

**Figure 2:**
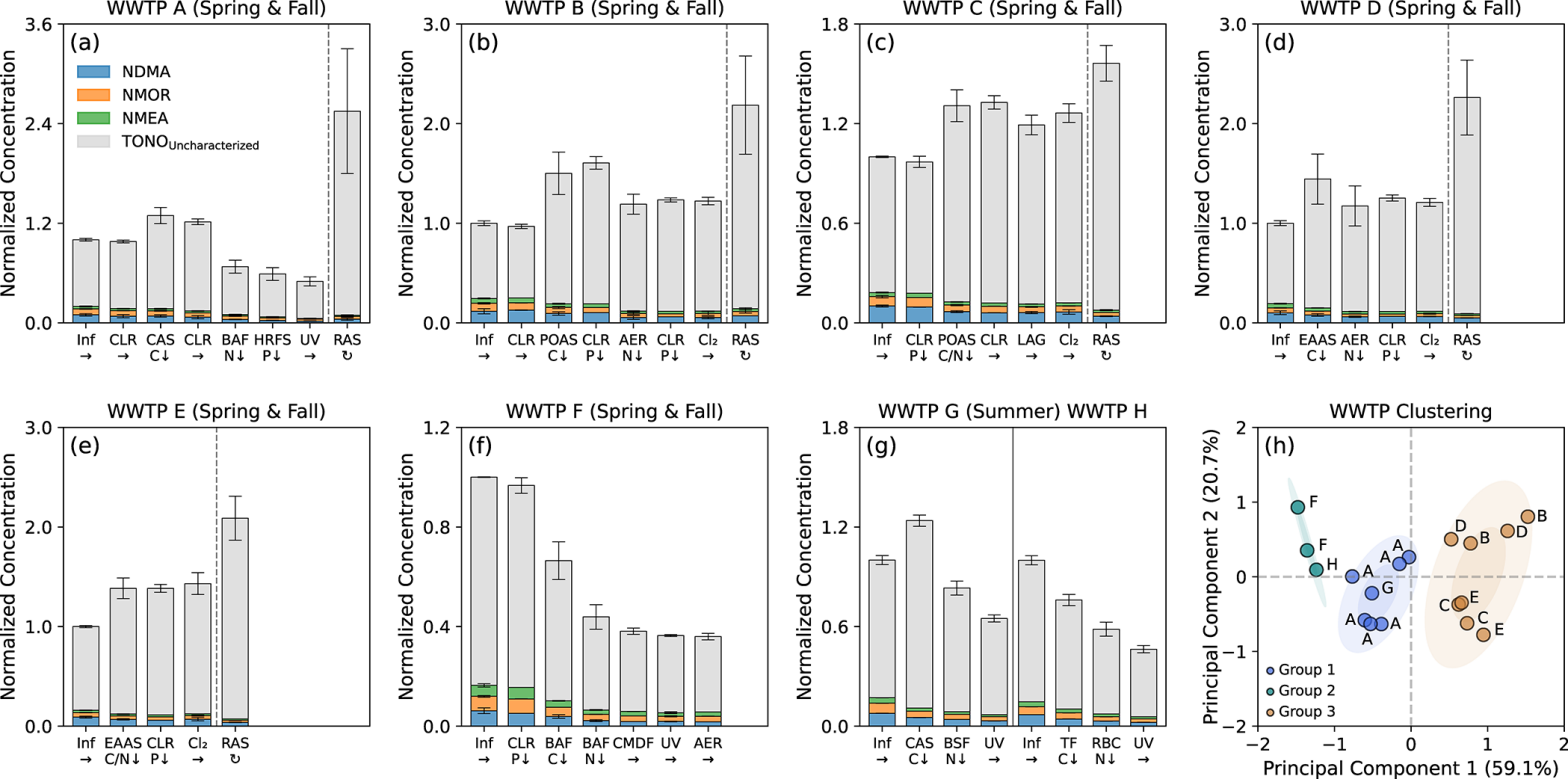
Compositional changes in TONO along the treatment trains of eight WWTPs: (a–g) Normalized concentration profiles of TONO (i.e., the sum of specific and uncharacterized *N*-nitrosamines) at WWTPs A-H. Profiles for different sampling events at the same WWTP were consolidated for clarity of presentation. Inf = plant influent; CLR = primary or secondary clarifier; CAS = conventional activated sludge aeration tank; POAS = pure oxygen activated sludge aeration tank; EAAS = extended aeration activated sludge aeration tank; AER = post-aeration tank; BAF = biological aerated filter; BSF = biological sand filter; TF = trickling filter; RBC = rotating biological contactor; HRFS = high-rate flocculated settling tank; CMDF = cloth media disc filter; LAG = lagoon; UV = UV irradiation; Cl_2_ = NaOCl application; and RAS = returned activated sludge. “ → ” indicates the flow direction. “↓” indicates BOD removal, nitrification, or phosphorus precipitation. “
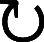
” indicates sludge recirculation. Error bars represent the standard deviations from duplicate measurements of TONO. Note the differences in the *y*-axis scales. (h) Principal component analysis of relative changes in the composition of TONO across secondary and tertiary treatment steps at WWTPs A-H for all sampling events (*n* = 18).

Concentrations of specific *N*-nitrosamines and TONO in samples from each WWTP were normalized against those in the corresponding plant influents and consolidated across sampling events for comparative analysis (Figure 2a–g). WWTPs A-E and G operated conventional, pure oxygen, or extended aeration activated sludge systems for BOD removal. On average, TONO in effluents (i.e., 3.4 ± 0.2 to 7.5 ± 0.3 nM) from the aeration tanks of these systems were 24 ± 6 to 50 ± 18% higher than those in plant influents (i.e., 2.1 ± 0.1 to 6.0 ± 0.2 nM), despite a 14 ± 3 to 36 ± 1% reduction in the summed concentrations of NDMA, NMOR, and NMEA in aeration tank effluents as observed at WWTPs in California and Switzerland.^14,15^ Concurrently, the fractional contribution of uncharacterized *N*-nitrosamines to TONO increased slightly from 81 ± 4% in plant influents to 89 ± 2% in aeration tank effluents. One plausible explanation for the lack of apparent TONO reduction during activated sludge treatment at these WWTPs was the recycling of TONO via RAS into aeration tanks. To quantify the contribution of RAS to the TONO budget, a mass balance analysis was performed on aeration tanks at WWTPs A-E using 24-h average flow rates and concentrations of *N*-nitrosamines measured in plant influents, aeration tank effluents, and RAS supernatants. Calculating TONO in aeration tanks with the assumption of plant influents and RAS as two major inflows yielded concentrations corresponding to 96 ± 5 to 102 ± 10% of those measured in aeration tank effluents. For these WWTPs, TONO in RAS (i.e., 5.3 ± 0.6 to 17 ± 1.8 nM) contributed between 34 ± 7 and 62 ± 8% to those entering aeration tanks, which supports the hypothesis that RAS might serve as an internal source of *N*-nitrosamines within activated sludge-based facilities. Carbonyl-catalyzed *N*-nitrosation (e.g., those involving a nucleophilic attack by nitrite on an iminium ion intermediate followed by the collapse of the adduct into *N*-nitrosamines)^45,46^ might partly explain the accumulation (in RAS) and/or external inputs (from plant influents) of *N*-nitrosamines. For instance, previous research has demonstrated that spiking nitrite and carbonyl compounds (e.g., formaldehyde) into sludge samples from WWTPs in the southeastern U.S. promoted the formation of NDMA from dimethylamine.^47^ More recent work has provided further evidence that carbonyl-catalyzed *N*-nitrosation contributed to fluctuating levels of NMOR in plant influents entering WWTPs in Japan^17^ and high concentrations of NDMA and NMOR in effluents discharged from chemical or pharmaceutical factories in Switzerland.^43,46^ One other possible factor contributing to elevated TONO in aeration tank effluents and RAS supernatants could be *N*-nitrosation mediated by nitrifying bacteria as previously observed during activated sludge treatment at WWTPs in Arkansas,^48^ Spain,^49,50^ and France.^51^ WWTPs F and H, on the other hand, operated biological aerated filters and trickling filters, respectively, for BOD removal, which reduced TONO in plant influents (i.e., 3.9 ± 0.2 to 5.5 ± 0.5 nM) by 24 ± 5 to 33 ± 6%. Comparable levels of TONO reduction (i.e., 23 ± 3 to 47 ± 4%) also occurred in biological aerated filters at WWTPs A and F, biological sand filters at WWTP G, and rotating biological contactors at WWTP H for nitrification. WWTP A further applied ferric for high-rate flocculated settling of phosphorus followed by low-pressure UV disinfection, which removed 27 ± 4% of TONO from nitrified effluents. WWTPs F and H also disinfected nitrified effluents with UV and achieved an additional 14 ± 3 to 22 ± 2% reduction in TONO. WWTPs B-E applied chlorination, which exerted a minimal impact on TONO as expected for nitrified effluents.^13,52^ Neither chlorination nor UV altered the fractional contribution of uncharacterized *N*-nitrosamines to TONO (i.e., 87 ± 4 to 91 ± 2%) in effluents (i.e., 1.4 ± 0.1 to 7.9 ± 0.3 nM) leaving these facilities.

Principal component analysis clustered WWTPs into three groups (Figure 2h) based on changes in the normalized concentration profiles of TONO components across secondary and tertiary treatment steps for all sampling events. For WWTPs (i.e., A and G) that utilized conventional activated sludge and attached growth systems for BOD removal and nitrification followed by UV, the aqueous removal efficiencies^15^ varied from 38 ± 10% for uncharacterized *N*-nitrosamines to 68 ± 12% for NDMA, resulting in a 43 ± 9% reduction in TONO in final effluents compared to plant influents. For WWTPs (i.e., F and H) that utilized attached growth systems for both BOD removal and nitrification followed by UV, the aqueous removal efficiencies for specific and uncharacterized *N*-nitrosamines reached 57 ± 6 to 67 ± 4%, which led to a 58 ± 6% reduction in TONO. For WWTPs (i.e., B–E) that utilized pure oxygen or extended aeration activated sludge systems for BOD removal and nitrification followed by chlorination, the aqueous removal efficiencies for specific *N*-nitrosamines ranged from 23 ± 8 to 51 ± 6%. However, TONO in final effluents exceeded those in plant influents by 21 ± 3 to 43 ± 11% due to a 35 ± 8 to 56 ± 12% increase in uncharacterized *N*-nitrosamines, indicating that these facilities might act as a source rather than a sink of TONO.

### Fate of TONO Precursors in WWTPs

Chloramine-reactive TONO precursors, approximated by the TONO formation potential (calculated as the difference in TONO between chloraminated and raw samples), ranged from 6.2 ± 0.2 to 125 ± 13 nM with a median of 24 nM (Figures S8–S10) and exhibited a linear relationship with organic nitrogen (i.e., estimated as the difference between total Kjeldahl nitrogen and ammonia nitrogen) in samples from WWTPs A-H (Figure S11). On average, each mg/L of organic nitrogen formed 6.1 ± 0.4 nM of TONO upon chloramination, similar to the 4.1 ± 2.5 nM per mg of organic nitrogen reported for effluents from WWTPs in Arkansas.^30^ Changes in the TONO formation potential also exhibited a linear relationship with variations in organic nitrogen along the treatment trains (Figure S12), further supporting the use of organic nitrogen as a surrogate for wastewater-derived TONO precursors.

On average, the TONO formation potential in effluents from aeration tanks (i.e., 17 ± 1 to 92 ± 3 nM) was 44 ± 2 to 81 ± 16% higher than that in plant influents (i.e., 11 ± 1 to 64 ± 1 nM) at WWTPs A-E and G, although the summed formation potential of specific *N*-nitrosamines was reduced by 14 ± 1 to 27 ± 7% in aeration tank effluents as previously observed during activated sludge treatment.^13,37,53^ Calculating TONO formation potential in aeration tank effluents using plant influents and RAS as the inputs also yielded values corresponding to 96 ± 4 to 100 ± 9% of that measured for samples from WWTPs A-E, pointing to RAS as a source of precursors in addition to plant influents. For example, precursors contributed by RAS (i.e., 30 ± 3 to 125 ± 13 nM) accounted for 43 ± 12 to 67 ± 4% of TONO formed upon chloramination of aeration tank effluents, which qualitatively aligned with the high levels of secondary amines and uncharacterized NDMA precursors measured in sludge samples from WWTPs in the southeastern U.S.^47^ Contrary to the patterns observed for activated sludge-based facilities, the TONO formation potential in effluents from attached growth systems (i.e., 15 ± 1 to 70 ± 2 nM) at WWTPs F and H for BOD removal was 18 ± 3 to 35 ± 8% lower than that in plant influents (i.e., 29 ± 1 to 85 ± 3 nM). Comparable levels of reduction in TONO formation potential (i.e., 14 ± 1 to 46 ± 4%) also occurred following nitrification in attached growth systems at WWTPs A and F−H. Consistent with the deactivation of NDMA precursors by preoxidation,^54^ UV and chlorination eliminated an additional 8 ± 2 to 35 ± 1% of the TONO formation potential in nitrified effluents. Compared to influents entering WWTPs A, B, D, F, G, and H, the TONO formation potential in final effluents (i.e., 6.2 ± 0.2 to 53 ± 3 nM) was 19 ± 7 to 63 ± 5% lower; in contrast, WWTPs C and E did not achieve net precursor reduction as the TONO formation potential in final effluents (i.e., 33 ± 1 to 82 ± 2 nM) was 24 ± 8 to 27 ± 11% higher than that in plant influents, suggesting their potential to serve as sources of TONO precursors.

**Figure 3:**
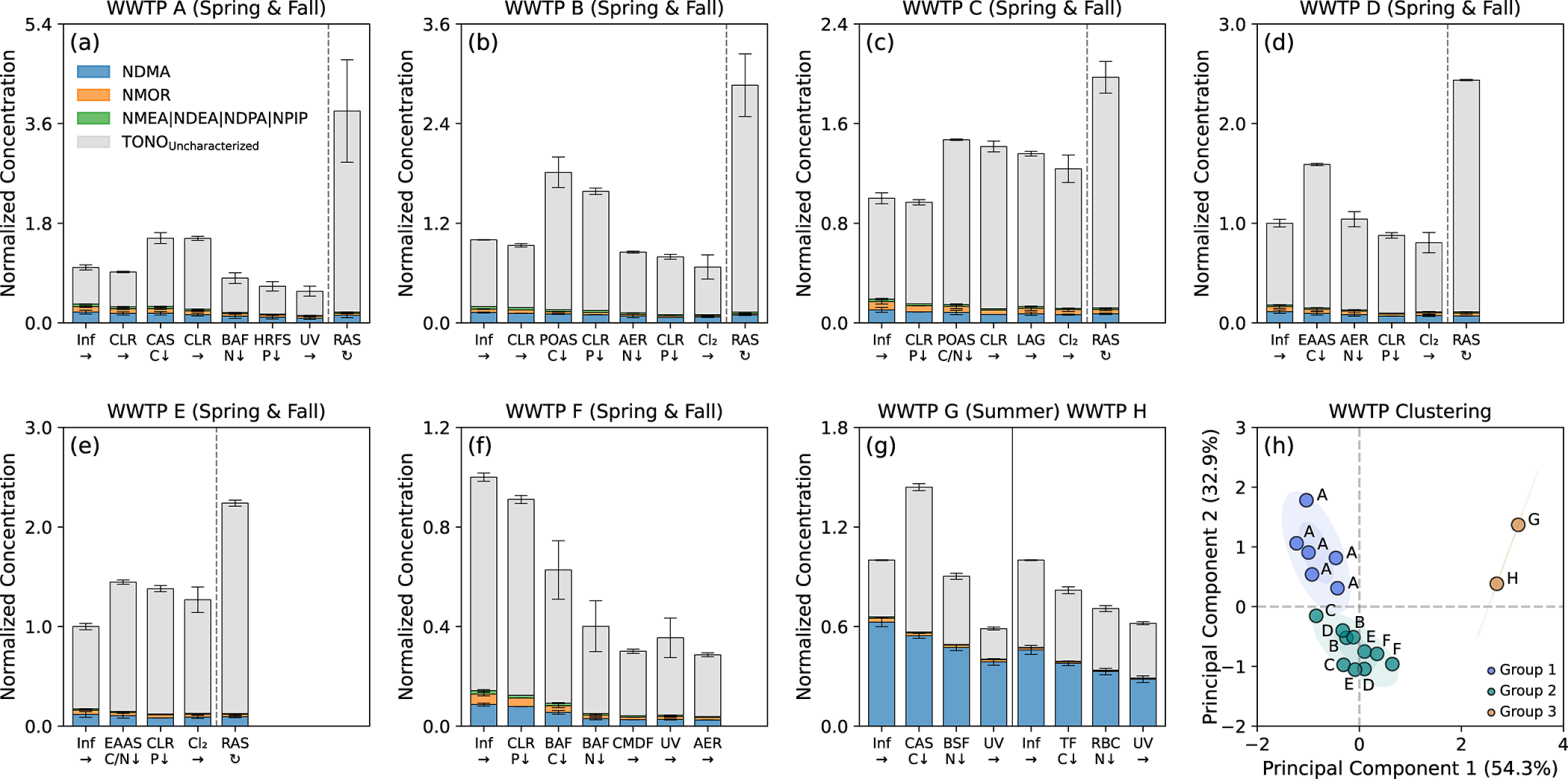
Compositional changes in TONO precursors along the treatment trains of WWTPs: (a–g) Normalized concentration profiles of TONO precursors (i.e., the sum of precursors to specific and uncharacterized *N*-nitrosamines) at WWTPs A-H. Profiles for different sampling events at the same WWTP were consolidated for the clarity of presentation. Inf = plant influent; CLR = primary or secondary clarifier; CAS = conventional activated sludge aeration tank; POAS = pure oxygen activated sludge aeration tank; EAAS = extended aeration activated sludge aeration tank; AER = post-aeration tank; BAF = biological aerated filter; BSF = biological sand filter; TF = trickling filter; RBC = rotating biological contactor; HRFS = high-rate flocculated settling tank; CMDF = cloth media disc filter; LAG = lagoon; UV = UV irradiation; Cl_2_ = NaOCl application; and RAS = returned activated sludge. “ → ” indicates the flow direction. “↓” indicates BOD removal, nitrification, or phosphorus precipitation. “
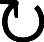
” indicates sludge recirculation. Error bars represent the standard deviations from duplicate measurements of TONO precursors. Note the differences in the *y*-axis scales. (h) Principal component analysis of relative changes in the composition of TONO precursors across secondary and tertiary treatment steps at WWTPs A-H for all sampling events (*n* = 18).

Principal component analysis clustered WWTPs based on changes in the normalized concentration profiles of TONO formation potential attributable to specific and uncharacterized components across secondary and tertiary treatment steps for all sampling events (Figure 3h). On average, the fractional contribution of NDMA precursors to TONO formation potential reached up to 55 ± 8 and 46 ± 2% in samples from WWTPs G and H, respectively, which far exceeded that in samples from WWTPs A-F (i.e., 8 ± 3 to 13 ± 4%). Conversely, the fractional contributions of uncharacterized TONO precursors were considerably higher in samples from WWTPs A-F (i.e., 78 ± 10 to 89 ± 5%) than in those from WWTPs G and H (i.e., 43 ± 13 and 53 ± 1%, respectively). Targeted LC–MS/MS analysis measured elevated concentrations of pharmaceuticals containing the *N,N*-dimethylamino moiety (e.g., metformin, methadone, and ranitidine) in influents entering WWTPs G and H (Table S9), which receive ∼20 and ∼25% of their inflow from a pharmaceutical manufacturing facility and a regional medical center, respectively. Metformin occurred at the highest concentrations in samples from WWTP G (i.e., ranging from 670 ± 27 nM in plant influents to 9.7 ± 0.4 nM in final effluents), followed by methadone (i.e., ranging from 90 ± 1 nM in plant influents to 65 ± 1 nM in final effluents) and ranitidine (i.e., ranging from 10 ± 0.2 nM in plant influents to 3.4 ± 0.1 nM in final effluents), respectively. Metformin (i.e., ranging from 620 ± 34 nM in plant influents to 63 ± 3 nM in final effluents) and ranitidine (i.e., ranging from 36 ± 2 nM in plant influents to 22 ± 1 nM in final effluents) also occurred at high concentrations in samples from WWTP H. Together, metformin, methadone, and ranitidine contributed to 89 ± 4% of NDMA formation potential in samples from WWTPs G and H. Multiplying their concentrations by respective NDMA molar yields (i.e., 0.23% for metformin, 91 ± 2% for ranitidine, and 28 ± 2% for methadone, as measured in pooled wastewater samples under the formation potential test conditions described above) and the TONO score for NDMA explained 34 ± 1 to 46 ± 3% of the TONO formation potential. On the contrary, WWTPs A-F predominantly treat domestic sewage, so dimethylamino-containing pharmaceuticals did not emerge as major contributors to TONO formation potential at these facilities. For example, these pharmaceuticals only accounted for 9 ± 3% of the TONO formation potential in plant influents and final effluents from WWTP A.

### Nontarget Screening beyond Target *N*-Nitrosamines

**Figure 4:**
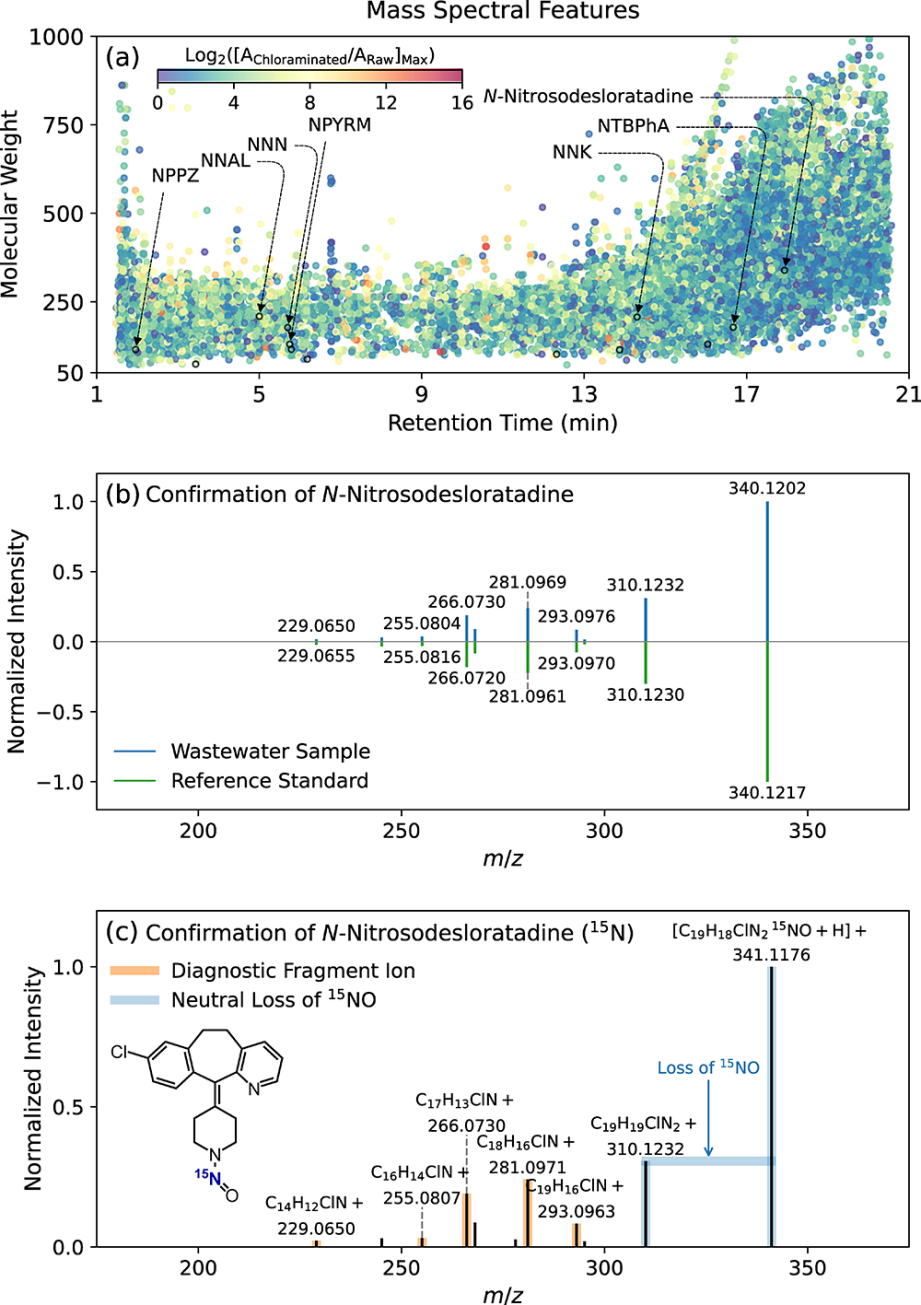
Nontargeted analysis of uncharacterized *N*-nitrosamines in wastewater samples: (a) Mass spectral features (nonredundant) with a range of molecular weights, retention times, and log 2-fold changes in peak area ratios between chloraminated and raw samples. The color bar measures the maximum log 2-fold changes in peak area ratios, ranging from low (blue) to high (red). Circles with black outlines represent mass spectral features confirmed at confidence level 1 by reference standards (including NDMA, NMOR, NMEA, NDEA, NDPA, and NPIP confirmed by targeted analysis). NNN = *N*-nitrosonornicotine; NNK = 4-(methylnitrosamino)-1-(3-pyridyl)-1-butanone; NNAL = 4-(methylnitrosamino)-1-(3-pyridyl)-1-butanol; NPPZ = *N*-nitrosopiperazine; NTBPhA = *N*-nitroso-*tert*-butylphenylamine; and NPYRM = *N*-nitroso-2-pyrrolidinmethanol. (b) Head-to-tail plot of experimental dd-MS2 spectra of *N*-nitrosodesloratadine measured for wastewater sample (top) and reference standard (bottom) with additional fragmentation information provided in Table S16. Head-to-tail plots of experimental dd-MS2 spectra of NNN, NNK, NNAL, NPPZ, NTBPhA, and NPYRM are shown in Figures S13–S18 with additional fragmentation information provided in Tables S10–S15. (c) Experimental dd-MS2 spectrum of ^15^N-labeled *N*-nitrosodesloratadine with diagnostic fragment ions and the neutral loss of ^15^NO as detailed in Table S17.

Nontargeted analysis of samples from WWTPs A-H led to the detection of mass spectral features spanning a range of molecular weights, polarities, and fold changes in peak intensities resulting from chloramination (Figure 4a). Seven of these features were further confirmed at confidence level 1 by reference standards as *N*-nitrosamines, including *N′-*nitrosonornicotine (NNN), 4-(*N*-nitrosomethylamino)-1-(3-pyridyl)-1-butanone (NNK), and 4-(methylnitrosamino)-1-(3-pyridyl)-1-butanol (NNAL), *N*-nitrosopiperazine, *N*-nitroso-*tert*-butylphenylamine, *N*-nitroso-2-pyrrolidinmethanol, and *N*-nitrosodesloratadine. NNN, NNK, and NNAL (Figures S13–S15) are tobacco-specific *N*-nitrosamines that have previously been detected at low nM levels (e.g., 0.01–0.04 nM for NNK and NNAL) in influent samples from WWTPs in Europe^55^ and Australia,^56^ and at higher concentrations (e.g., up to 0.28 nM for NNK and 1.7 nM for NNAL) in chloraminated secondary and tertiary effluent samples from WWTPs in Canada.^57^ Together, NNN, NNK, and NNAL only constituted ∼0.1% of TONO due to their low concentrations (i.e., 0.08 ± 0.02 to 0.18 ± 0.03 nM in a subset of samples from WWTPs A, D, and E) and low TONO scores (e.g., from 0.24 ± 0.02 for NNN to 0.26 ± 0.02 for NNK). *N*-Nitrosopiperazine (Figure S16) is a cyclic *N*-nitrosamine known to form via the nitrosation of piperazine in postcombustion CO_2_ capture systems,^58^ whereas *N*-nitroso-*tert*-butylphenylamine (Figure S17) and *N*-nitroso-2-pyrrolidinmethanol (Figure S18) were detected for the first time in wastewater. *N*-Nitrosopiperazine (i.e., 0.36 ± 0.08 nM in a subset of samples from WWTPs E and F), *N*-nitroso-*tert*-butylphenylamine (i.e., 0.39 ± 0.27 nM in a subset of samples from WWTPs A, E, and F), and *N*-nitroso-2-pyrrolidinmethanol (i.e., 0.22 ± 0.12 nM in a subset of samples from WWTP E) were also minor TONO components and constituted only 0.1–0.7% of TONO.

*N*-Nitrosodesloratadine (i.e., 0.07 ± 0.01 nM in a subset of samples from WWTPs E and F; Figure 4b) is an *N*-nitroso derivative of desloratadine, a tricyclic antihistamine used for treating allergic rhinitis and chronic idiopathic urticaria.^59^ Multiple classes of secondary amine-containing pharmaceuticals, including desloratadine, are prone to acid-catalyzed *N*-nitrosation in the presence of nitrite (e.g., in the human stomach^60^ or during drug manufacturing^61^) to form corresponding *N*-nitroso derivatives with the potential to induce positive responses in short-term genotoxicity tests and/or long-term carcinogenesis assays.^62^ Given the widespread occurrence of secondary amine-containing pharmaceuticals as organic micropollutants in wastewater, their *N*-nitroso derivatives may represent a class of transformation products. For example, multiple studies have reported the presence of *N*-nitroso derivatives, such as *N*-nitroso-*N′*-phenylpiperazine (a building block found in fluoroquinolones),^48^*N*-nitrosodiclofenac,^49,50^*N*-nitrosociprofloxacin,^51^ and *N*-nitrosohydrochlorothiazide,^51^ in samples from WWTPs employing activated sludge systems. To investigate the extent to which such *N*-nitroso derivatives may form under chloramination conditions, formation potential tests were performed to assess the nitrosatability of desloratadine as well as eight other pharmaceuticals (or building blocks) containing dialkyl or (cyclic)diaryl secondary amine moieties (i.e., fenfluramine, fluoxetine, betahistine, nebivolol, metoprolol, desipramine, nortriptyline, and iminodibenzyl). Chloramination of desloratadine with preformed NH_2_Cl or ^15^NH_2_Cl indeed led to the formation of *N*-nitrosodesloratadine or its ^15^N-labeled analog (Figure 4c). Control chloramination experiments conducted inside a vinyl anaerobic chamber (Coy Laboratory Products) and/or with the addition of 6-hydroxy-2,5,7,8-tetramethyl-3,4-dihydrochromene-2-carboxylic acid (Trolox) as a scavenger^63,64^ resulted in the suppression of *N*-nitrosodesloratadine formation, suggesting the potential involvement of dissolved O_2_ as well as peroxynitrite and its conjugate acid in the *N*-nitrosation of desloratadine as hypothesized for the reactive nitrogen species-mediated formation pathway of NDMA from dimethylamine.^65^ For example, the one-electron oxidation of desloratadine by nitryl and/or hydroxyl radicals generated through the homolytic decomposition of peroxynitrous acid^66^ might lead to the formation of its aminyl radical, which further reacted with nitric oxide^67−69^ to yield *N*-nitrosodesloratadine (Figure S29). On the other hand, nucleophilic substitution between dichloramine and desloratadine might form its chlorinated unsymmetrical hydrazine derivative,^70^ which subsequently reacted with peroxynitrous acid^65^ to produce *N*-nitrosodesloratadine (Figure S30). Chloramination of the other eight secondary amine-containing precursors also confirmed the formation of their corresponding *N*-nitroso derivatives (Tables S18–S25 and Figures S21–S28). Concurrent analysis of chloraminated samples by LC-HRMS and HI_3_–CL showed that the molar yields of *N*-nitroso derivatives ranged from 0.8 ± 0.1% for iminodibenzyl to 1.5 ± 0.1% for fluoxetine, as measured by LC-HRMS, which were lower than the yield of NDMA from dimethylamine (i.e., 2.5 ± 0.2%). Nevertheless, yields measured by HI_3_–CL were higher, ranging from 2.0 ± 0.1% for fenfluramine to 4.2 ± 0.1% for metoprolol (Figure S31), likely due to contributions from products other than *N*-nitroso derivatives. For instance, *N*-nitrosoiminodibenzyl emerged as an additional *N*-nitrosation product of desipramine upon chloramination (e.g., via the nitrosative cleavage of its cyclic tertiary amine moiety^71^).

### Environmental Implications

This study improved the characterization of TONO in wastewater matrices by developing an analytical framework to quantitatively relate LC-HRMS data to chemiluminescence measurements. Through the parallel application of LC-HRMS and HI_3_–CL, we demonstrated that correcting the concentrations of specific *N*-nitrosamines measured by LC-HRMS with their SPE recoveries and conversion efficiencies to nitric oxide more accurately reflected their fractional contributions to TONO, although the predominance of uncharacterized TONO and their precursors in wastewater still holds as observed by previous work.^27−29^ Matrix-specific TONO scores should be determined for both known and newly identified *N*-nitrosamines, as inconsistencies in their recoveries across different aqueous matrices can result in deviations from the TONO scores reported herein. Our comparative analysis further revealed high variability in the reduction and/or accumulation of TONO and their precursors along the treatment trains of conventional WWTPs such as those sampled in this study, with some serving as sinks, while others acted as sources depending on the nature of plant influents and the configurations of secondary and tertiary treatment processes. Our nontarget screening workflow prioritized additional *N*-nitrosamines beyond traditionally targeted species for confirmation, even though these newly identified species did not necessarily represent quantitatively significant components of TONO. Our efforts to confirm the identities of unknown *N*-nitrosamines by LC-HRMS, however, were still constrained to those with reference standards, especially given the potential interferences from isobaric or isomeric substances in wastewater. Moreover, moieties in structurally complex amine precursors may exhibit a range of nitrosatability under oxidative treatment conditions, as illustrated in the case of secondary amine-containing pharmaceuticals, whose *N*-nitroso derivatives explained only a fraction of the chemiluminescence signals following chloramination due to the concomitant formation of nontarget *N*-nitrosamines. Complementary preconcentration techniques and physicochemical fractionation methods should be implemented in future work to capture the fraction of TONO not effectively captured by the SPE protocol applied in our study and to identify and quantify TONO precursors associated with particles, colloids, or macromolecules.^13,37^ For instance, the formation potential of uncharacterized TONO showed strong positive correlations not only with the maximum fluorescence intensity of a protein-like organic matter component (i.e., excitation/emission maxima around 275/340 nm; Figure S32) but also with the intensities of tyrosine-like and tryptophan-like fluorescent organic matter fractions (i.e., Peak B and Peak T, respectively; Figures S33–S34). Continued efforts are therefore warranted to assess the nitrosatability of precursors of proteinaceous nature^30^ given that they comprise a significant fraction of organic nitrogen in wastewater.^72^ For example, peptides such as those containing tryptophan and proline are known to undergo acid-catalyzed *N*-nitrosation in the presence of nitrite,^73−75^ potentially contributing to TONO formation if stable *N*-nitroso products other than diazopeptides^74^ are formed upon chloramination. Overall, our study moves beyond source-related quantification of TONO, provides new insights into the fate of TONO and their precursors in WWTPs, and establishes an analytical framework to support continued research into the identification of uncharacterized *N*-nitrosamines in environmental systems.

## Supporting Information Available

Sampling events at eight WWTPs; physicochemical and optical properties of wastewater samples; LC-HRMS instrument settings and method parameters; nontarget screening workflow settings; TONO scores for specific *N*-nitrosamines; concentration profiles of specific *N*-nitrosamines and TONO across all sampling events; concentration ranges of pharmaceuticals and other wastewater-derived substances measured by LC–MS/MS; mass spectral data of nontargeted *N*-nitrosamines confirmed in wastewater samples by reference standards; and mass spectral data and yields of *N*-nitroso derivatives formed from nine secondary amine-containing pharmaceuticals (PDF)
