# Supplementary material for: Combining High-Resolution Mass Spectrometry and Chemiluminescence Analysis to Characterize the Composition and Fate of Total N-Nitrosamines in Wastewater Treatment Plants
Source: Environ Sci Technol. 2024 Sep 10;58(38):17081–91. doi: 10.1021/acs.est.4c06555 (PMC11428135; doi:10.1021/acs.est.4c06555)
Supplement: Supplementary file 1 — es4c06555_si_001.pdf [file es4c06555_si_001.pdf]

Supporting Information for

Combining High-Resolution Mass Spectrometry and Chemiluminescence Analysis to Characterize the Composition and Fate of Total *N*-Nitrosamines in Wastewater Treatment Plants

Changcheng Pu[1], Benjamin R. Cavarra[1], Teng Zeng[,1]*

[1]Department of Civil and Environmental Engineering, Syracuse University, 151 Link Hall, Syracuse, New York 13244, United States

*Corresponding Author: Teng Zeng: Email: tezeng@syr.edu; Phone: +1-315-443-1099

(Total 86 pages, 2 texts, 25 tables, 34 figures)

Table of Contents

1. Chemicals, reagents, and supplies S3

2. WWTP sampling information S7

3. Physicochemical and optical properties of wastewater samples S10

4. LC-HRMS instrument settings and method parameters S16

5. Nontarget screening workflow settings S20

6. Summary of TONO scores for specific *N*-nitrosamines S33

7. Concentration profiles of specific *N*-nitrosamines and TONO across sampling events S37

8. Covariation of TONO formation potential with organic nitrogen S43

9. Concentration ranges of pharmaceuticals and other wastewater-derived substances S44

10. Confirmation of nontargeted *N*-nitrosamines by reference standards in wastewater samples S47

11. Formation of *N*-nitroso derivatives from pharmaceuticals upon chloramination S61

12. Covariation of uncharacterized TONO formation potential with protein-like organic matter S84

References S86

1. Chemicals, reagents, and supplies

Chemicals and reagents were used as received without further purification. Methanol (HPLC and LC-MS grade), water (HPLC and LC-MS grade), methylene chloride (Optima grade), pentane (HPLC grade), ethyl acetate (HPLC grade), acetic acid (glacial HPLC grade), formic acid solution (FA; ≥99.0%; LC-MS grade), poly(ethylene glycol) (average M.W. 6000), sodium hypochlorite solution (NaOCl; 5.65-6% laboratory grade), sodium sulfate (anhydrous, ≥99.0%), and sodium hydroxide (>99%) were purchased from Fisher Scientific. Ammonium chloride (NH_4Cl; 99.5%), L-ascorbic acid (99%), potassium iodide (99+%), iodine (99.5%), sodium dihydrogen phosphate monohydrate (99+%), and sodium phosphate dibasic heptahydrate (99+%) were purchased from ACROS Organics. Sulfamic acid (99.3%), *N,N*-diethyl-*p*-phenylenediamine sulfate salt (≥98.0%), and (±)-6-hydroxy-2,5,7,8-tetramethylchromane-2-carboxylic acid (Trolox; 97%) were purchased from Sigma-Aldrich. ^{15}N Labeled ammonium chloride ($^{15}NH_4Cl$; 99%) was purchased from Cambridge Isotope Laboratories.

N-Nitroso compound (NOC) reference standards and isotope-labeled internal standards (ILIS) were purchased from Enamine, Sigma-Aldrich, Toronto Research Chemicals, and C/D/N Isotopes as high-purity substances (**Table S1**). Stock solutions of NOCs were prepared by dissolving or diluting a gravimetrically weighted amount of solid or liquid reference standards into ultrapure water or methanol. Calibration standards were prepared by diluting predetermined volumes of stock solutions into ultrapure water.

Preformed monochloramine (NH_2Cl or $^{15}NH_2Cl$) working solutions were prepared by dissolving NH_4Cl or $^{15}NH_4Cl$ solids in ultrapure water (resistivity 18.2 MΩ•cm; produced by a Thermo Scientific Barnstead MicroPure UV/UF water purification system) adjusted to pH 9, followed by dropwise addition of standardized NaOCl solution to obtain a Cl:N molar ratio of 1:1.2. The concentration of NH_2Cl or $^{15}NH_2Cl$ was determined spectrophotometrically using a Thermo Scientific Evolution 201 UV-visible spectrophotometer by deconvoluting solution absorbance at 245 nm ($\varepsilon_{245nm}(NH_2Cl) = 445\ M^{-1}\ cm^{-1}$) and 295 nm ($\varepsilon_{295nm}(NHCl_2) = 14\ M^{-1}\ cm^{-1}$).[1]

Two SPE cartridges, Strata-X (33 µm polymeric reversed phase, 200 mg/6 mL) and Enviro-Clean 521 (coconut shell activated carbon, 2000 mg/15 mL), were purchased from Phenomenex and United Chemical Technologies, respectively. Quartz cuvettes (1-I-10 and 3-Q-10) used for spectrophotometry were purchased from Starna Cells.

High-density polyethylene (HDPE) sampling bottles, large-volume sample transfer tubes, and microsyringes were rinsed 5 times with HPLC grade methanol, followed by 5 times with ultrapure water, and dried overnight. Volumetric glassware and purge vessels were rinsed with HPLC grade methanol and ultrapure water and dried at 70 °C in a Fisherbrand Isotemp general purpose heating and drying oven. Non-volumetric glassware was rinsed and combusted at 450 °C in a Thermo Scientific Lindberg/Blue M Moldatherm box furnace for 5 h.

**Table S1.** List of *N*-nitrosamine reference standards and isotope-labeled internal standards							
Compound	Acronym	Supplier	Purity	CAS	Molecular Formula	Molecular Weight	Category
N-Nitrosodimethylamine	NDMA	Sigma-Aldrich	99.9%	62-75-9	$C_2H_6N_2O$	74.08	dialkyl
N-Nitrosomethylethylamine	NMEA	TRC	97.0%	10595-95-6	$C_3H_8N_2O$	88.11	dialkyl
N-Nitrosodiethylamine	NDEA	TRC	98.0%	55-18-5	$C_4H_{10}N_2O$	102.14	dialkyl
N-Nitrosodipropylamine	NDPA	TRC	98.0%	621-64-7	$C_6H_{14}N_2O$	130.19	dialkyl
N-Nitrosodibutylamine	NDBA	TRC	98.0%	924-16-3	$C_8H_{18}N_2O$	158.24	dialkyl
N-Nitrosopyrrolidine	NPYR	TRC	98.0%	930-55-2	$C_4H_8N_2O$	100.12	cyclic
N-Nitrosopiperidine	NPIP	TRC	98.0%	100-75-4	$C_5H_{10}N_2O$	114.15	cyclic
N-Nitrosomorpholine	NMOR	TRC	98.0%	59-89-2	$C_4H_8N_2O_2$	116.12	heterocyclic
N-Nitrosodiphenylamine	NDPhA	TRC	98.0%	86-30-6	$C_{12}H_{10}N_2O$	198.22	diaryl
N-Nitrosodibenzylamine	NDBzA	TRC	98.0%	5336-53-8	$C_{14}H_{14}N_2O$	226.28	diaryl
N-Nitrosodiethanolamine	NDELA	TRC	98.0%	1116-54-7	$C_4H_{10}N_2O_3$	134.14	dialkyl
N-Nitrosohydroxyproline	NHPRO	TRC	95.0%	30310-80-6	$C_5H_8N_2O_4$	160.13	cyclic
N-Nitrosoproline	NPRO	TRC	98.0%	7519-36-0	$C_5H_8N_2O_3$	144.13	cyclic
N-Nitrososarcosine	NSAR	TRC	98.0%	13256-22-9	$C_3H_6N_2O_3$	118.09	dialkyl
N-Nitroso-2-Methylthiazolidine 4-Carboxylic Acid	NMTCA	TRC	95.0%	103659-08-1	$C_5H_8N_2O_3S$	176.20	heterocyclic
N-Nitrosothiazolidine-4-Carboxylic Acid	NTCA	TRC	98.0%	88381-44-6	$C_4H_6N_2O_3S$	162.17	heterocyclic
N-Nitrosoanabasine	NAB	TRC	99.8%	1133-64-8	$C_{10}H_{13}N_3O$	191.23	cyclic
N-Nitrosoanatabine	NAT	TRC	99.8%	887407-16-1	$C_{10}H_{11}N_3O$	189.22	cyclic
N-Nitrosonornicotine	NNN	TRC	100.0%	16543-55-8	$C_9H_{11}N_3O$	177.21	cyclic
4-(Methylnitrosamino)-1-(3-Pyridyl)-1-Butanone	NNK	TRC	100.0%	64091-91-4	$C_{10}H_{13}N_3O_2$	207.23	dialkyl
4-(Methylnitrosamino)-1-(3-Pyridyl)-1-Butanol	NNAL	TRC	95.0%	76014-81-8	$C_{10}H_{15}N_3O_2$	209.25	dialkyl
N-Nitrosodicyclohexylamine	NDChA	TRC	98.0%	947-92-2	$C_{12}H_{22}N_2O$	210.32	dicyclic
N-Nitrosomethylphenylamine	NMPhA	TRC	95.0%	614-00-6	$C_7H_8N_2O$	136.15	alkylaryl
N-Nitrosopiperazine	NPPZ	TRC	98.0%	5632-47-3	$C_4H_9N_3O$	115.14	heterocyclic
N-Nitrosoatrazine	NATR	TRC	98.0%	56525-09-8	$C_8H_{13}ClN_6O$	244.68	alkylheteroaryl
N-Nitrososimazine	NSIM	TRC	99.5%	6494-81-1	$C_7H_{11}ClN_6O$	230.66	alkylheteroaryl
1-Nitroso-4-Phenylpiperazine	NPhPPZ	TRC	98.0%	14340-33-1	$C_{10}H_{13}N3O$	191.23	heterocyclic
N-Nitroso-*tert*-Butylphenylamine	NTBPhA	Sigma-Aldrich	97.0%	24642-84-0	$C_{10}H_{14}N_2O$	178.23	alkylaryl
N-Nitrosofenfluramine	NFFA	TRC	97.0%	19023-40-6	$C_{12}H_{15}F_3N_2O$	260.26	dialkyl
N-Nitrosomethylamylamine	NMAA	TRC	97.0%	13256-07-0	$C_6H_{14}N_2O$	130.19	dialkyl
N-Nitrosodiisopropylamine	NDIPA	TRC	97.0%	601-77-4	$C_6H_{14}N_2O$	130.19	dialkyl
N-Nitrosodiamylamine	NDAA	TRC	97.0%	13256-06-9	$C_{10}H_{22}N_2O$	186.30	dialkyl
N-Nitrosodiisobutylamine	NDIBA	TRC	97.0%	997-95-5	$C_8H_{18}N_2O$	158.24	dialkyl
1-Nitroso-1,2,3,4-Tetrahydroquinoline	NTHQ	Enamine	95.0%	5825-44-5	$C_9H_{10}N_2O$	162.19	cyclic
N-Nitrosomethylbutylamine	NMBA	Enamine	95.0%	7068-83-9	$C_5H_{12}N_2O$	116.16	dialkyl
N-Nitrosoethylbenzylamine	NEBzA	Enamine	95.0%	20689-96-7	$C_9H_{12}N_2O$	164.21	alkylaryl
N-Nitrosomethylisopropylamine	NMIPA	Enamine	95.0%	30533-08-5	$C_4H_{10}N_2O$	102.14	dialkyl
N-Nitroso-*tert*-Butylmethylamine	NTBMA	Enamine	95.0%	2504-18-9	$C_5H_{12}N_2O$	116.16	dialkyl
N-Nitrosoethylpropylamine	NEPA	Enamine	95.0%	25413-61-0	$C_5H_{12}N_2O$	116.16	dialkyl

Table S1. List of *N*-nitrosamine reference standards and isotope-labeled internal standards (continued)							
Compound	Acronym	Supplier	Purity	CAS	Molecular Formula	Molecular Weight	Category
N-Nitrosomethylisobutylamine	NMIBA	Enamine	95.0%	34419-76-6	$C_5H_{12}N_2O$	116.16	dialkyl
N-Nitrosopipecolic Acid	NPIC	Enamine	95.0%	4515-18-8	$C_6H_{10}N_2O_3$	158.16	cyclic
N-Nitroso-2-Pyrrolidinmethanol	NPYRM	Enamine	95.0%	68292-94-4	$C_5H_{10}N_2O_2$	130.15	cyclic
N-Nitroso-4-Methylaminopyridine	NMAPY	Enamine	95.0%	16219-99-1	$C_6H_7N_3O$	137.14	alkylheteroaryl
N-Nitrosoethylphenylamine	NEPhA	Enamine	95.0%	612-64-6	$C_8H_{10}N_2O$	150.18	alkylaryl
N-Nitrosomethylcyclohexylamine	NMChA	Enamine	95.0%	5432-28-0	$C_7H_{14}N_2O$	142.20	alkylcyclic
N-Nitrosoindoline	NIND	Enamine	95.0%	7633-57-0	$C_8H_8N_2O$	148.16	cyclic
N-Nitroso-*tert*-Butylethylamine	NTBEA	Enamine	95.0%	3398-69-4	$C_6H_{14}N_2O$	130.19	dialkyl
N-Nitrosomethyl-*N,N*-Dimethylethylamine	NMDEA	Enamine	95.0%	23834-30-2	$C_5H_{13}N_3O$	131.18	dialkyl
1-Nitroso-4-Methylpiperidine	NMPIP	Enamine	95.0%	15104-03-7	$C_6H_{12}N_2O$	128.17	cyclic
N-Nitrosodimethyl-d_6-Amine	NDMA-d_6	C/D/N	98% D	17829-05-9	$C_2D_6N_2O$	80.12	ILIS
N-Nitrosodiethyl-d_{10}-Amine	NDEA-d_{10}	C/D/N	99% D	1219794-54-3	$C_4D_{10}N_2O$	112.20	ILIS
N-Nitrosodi-*n*-Propyl-d_{14}-Amine	NDPA-d_{14}	C/D/N	98% D	93951-96-3	$C_6D_{14}N_2O$	144.27	ILIS
N-Nitrosopyrrolidine-d_8	NPYR-d_8	C/D/N	98% D	1219802-09-1	$C_4D_8N_2O$	108.17	ILIS
N-Nitrosomorpholine-d_8	NMOR-d_8	C/D/N	98% D	1219805-76-1	$C_4D_8N_2O_2$	124.17	ILIS
N-Nitrosodiphenyl-2,2',4,4',6,6'-d_6-Amine	NDPhA-d_6	C/D/N	98% D	93951-95-2	$C_{12}H_4D_6N_2O$	204.26	ILIS
N-Nitrosobis(2-Hydroxyethyl)-d_8-Amine	NDELA-d_8	C/D/N	98% D	1173019-53-8	$C_4H_2D_8N_2O$	142.18	ILIS
N-Methyl-d_3-*N*-nitrosoglycine	NSAR-d_3	C/D/N	99% D	1189871-94-0	$C_3H_3D_3N_2O_3$	121.11	ILIS

TRC = Toronto Research Chemicals. C/D/N = C/D/N Isotopes. ILIS = Isotope-labeled internal standard.

2. WWTP sampling information

Table S2. Summary of WWTP sampling events and locations

Facility ID	Service Population	Average Design Hydraulic Flow (MGD)
WWTP A	242377	84.2
WWTP B	30613	9.0
WWTP C	25965	7.0
WWTP D	16168	3.0
WWTP E	50727	10.0
WWTP F	39352	6.5
WWTP G	385	0.18
WWTP H	1924	0.75
**Sample ID**	**Season**	**Sampling Location**
WWTP A-1703-S1	Spring	Plant influent
WWTP A-1703-S2		Primary clarifier effluent
WWTP A-1703-S3		Aeration tank effluent
WWTP A-1703-S4		Secondary clarifier effluent
WWTP A-1703-S5		Biological aerated filter effluent
WWTP A-1703-S6		High-rate flocculated settling tank effluent
WWTP A-1703-S7		Low-pressure UV effluent
WWTP A-1703-S8		Returned activated sludge (RAS)
WWTP A-1709-W1-S1	Fall (Event #1)	Plant influent
WWTP A-1709-W1-S2		Primary clarifier effluent
WWTP A-1709-W1-S3		Aeration tank effluent
WWTP A-1709-W1-S4		Secondary clarifier effluent
WWTP A-1709-W1-S5		Biological aerated filter effluent
WWTP A-1709-W1-S6		High-rate flocculated settling tank effluent
WWTP A-1709-W1-S7		Low-pressure UV effluent
WWTP A-1709-W1-S8		Returned activated sludge (RAS)
WWTP A-1709-W2-S1	Fall (Event #2)	Plant influent
WWTP A-1709-W2-S2		Aeration tank effluent
WWTP A-1709-W2-S3		Biological aerated filter effluent
WWTP A-1709-W2-S4		High-rate flocculated settling tank effluent
WWTP A-1709-W2-S5		Low-pressure UV effluent
WWTP A-1709-W2-S6		Returned activated sludge (RAS)
WWTP A-1709-W3-S1	Fall (Event #3)	Plant influent
WWTP A-1709-W3-S2		Aeration tank effluent
WWTP A-1709-W3-S3		Biological aerated filter effluent
WWTP A-1709-W3-S4		High-rate flocculated settling tank effluent
WWTP A-1709-W3-S5		Low-pressure UV effluent
WWTP A-1709-W3-S6		Returned activated sludge (RAS)
WWTP A-1710-W4-S1	Fall (Event #4)	Plant influent
WWTP A-1710-W4-S2		Aeration tank effluent
WWTP A-1710-W4-S3		Biological aerated filter effluent
WWTP A-1710-W4-S4		High-rate flocculated settling tank effluent
WWTP A-1710-W4-S5		Low-pressure UV effluent
WWTP A-1710-W4-S6		Returned activated sludge (RAS)
WWTP A-1710-W5-S1	Fall (Event #5)	Plant influent
WWTP A-1710-W5-S2		Aeration tank effluent
WWTP A-1710-W5-S3		Biological aerated filter effluent
WWTP A-1710-W5-S4		High-rate flocculated settling tank effluent
WWTP A-1710-W5-S5		Low-pressure UV effluent
WWTP A-1710-W5-S6		Returned activated sludge (RAS)

**Table S2.** Summary of WWTP sampling events (continued)		
Sample ID	Season	Sampling Location
WWTP B-1703-S1	Spring	Plant influent
WWTP B-1703-S2		Primary clarifier effluent
WWTP B-1703-S3		1st Stage pure oxygen aeration tank effluent
WWTP B-1703-S4		1st Stage secondary clarifier effluent
WWTP B-1703-S5		2nd Stage aeration tank effluent
WWTP B-1703-S6		2nd Stage secondary clarifier effluent
WWTP B-1703-S7		Chlorine contact tank effluent
WWTP B-1703-S8		Returned activated sludge (RAS)
WWTP B-1710-S1	Fall	Plant influent
WWTP B-1710-S2		1st Stage pure oxygen aeration tank effluent
WWTP B-1710-S3		2nd Stage aeration tank effluent
WWTP B-1710-S4		Chlorine contact tank effluent
WWTP B-1710-S5		Returned activated sludge (RAS)
WWTP C-1704-S1	Spring	Plant influent
WWTP C-1704-S2		Primary clarifier effluent
WWTP C-1704-S3		Pure oxygen aeration tank effluent
WWTP C-1704-S4		Secondary clarifier effluent
WWTP C-1704-S5		Lagoon effluent
WWTP C-1704-S6		Chlorine contact tank effluent
WWTP C-1704-S7		Returned activated sludge (RAS)
WWTP C-1709-S1	Fall	Plant influent
WWTP C-1709-S2		Pure oxygen aeration tank effluent
WWTP C-1709-S3		Lagoon effluent
WWTP C-1709-S4		Chlorine contact tank effluent
WWTP C-1709-S5		Returned activated sludge (RAS)
WWTP D-1703-S1	Spring	Plant influent
WWTP D-1703-S2		1st Stage extended aeration tank effluent
WWTP D-1703-S3		2nd Stage aeration tank effluent
WWTP D-1703-S4		Secondary clarifier effluent
WWTP D-1703-S5		Chlorine contact tank effluent
WWTP D-1703-S6		Returned activated sludge (RAS)
WWTP D-1709-S1	Fall	Plant influent
WWTP D-1709-S2		1st Stage extended aeration tank effluent
WWTP D-1709-S3		2nd Stage aeration tank effluent
WWTP D-1709-S4		Chlorine contact tank effluent
WWTP D-1709-S5		Returned activated sludge (RAS)
WWTP E-1704-S1	Spring	Plant influent
WWTP E-1704-S2		Extended aeration tank effluent
WWTP E-1704-S3		Secondary clarifier effluent
WWTP E-1704-S4		Chlorine contact tank effluent
WWTP E-1704-S5		Returned activated sludge (RAS)
WWTP E-1709-S1	Fall	Plant influent
WWTP E-1709-S2		Extended aeration tank effluent
WWTP E-1709-S3		Chlorine contact tank effluent
WWTP E-1709-S4		Returned activated sludge (RAS)

**Table S2.** Summary of WWTP sampling events (continued)		
Sample ID	Season	Sampling Location
WWTP F-1703-S1	Spring	Plant influent
WWTP F-1703-S2		Primary clarifier effluent
WWTP F-1703-S3		Biological aerated filter C cell effluent
WWTP F-1703-S4		Biological aerated filter N cell effluent
WWTP F-1703-S5		Cloth media disc filter effluent
WWTP F-1703-S6		Low-pressure UV effluent
WWTP F-1703-S7		Post-aeration tank effluent
WWTP F-1710-S1	Fall	Plant influent
WWTP F-1710-S2		Biological aerated filter C cell effluent
WWTP F-1710-S3		Biological aerated filter N cell effluent
WWTP F-1710-S4		Low-pressure UV effluent
WWTP G-1708-S1	Summer	Plant influent
WWTP G-1708-S2		Aeration tank effluent
WWTP G-1708-S3		Biological sand filter effluent
WWTP G-1708-S4		Low-pressure UV effluent
WWTP H-1708-S1	Summer	Plant influent
WWTP H-1708-S2		Trickling filter effluent
WWTP H-1708-S3		Rotating biological contactor effluent
WWTP H-1708-S4		Low-pressure UV effluent

3. Physicochemical and optical properties of wastewater samples

General wastewater quality parameters (e.g., BOD_5, total suspended solids, total Kjeldahl nitrogen, ammonia nitrogen, and total phosphorus) were provided by the WWTPs. Small aliquots of filtered wastewater samples (~5 mL) were diluted to an absorbance at 254 nm of <0.6 for optical characterization at Syracuse University. Fluorescence excitation-emission matrices (EEMs) and UV-visible absorbance spectra for each sample were measured in duplicate using a Horiba Scientific Aqualog spectrofluorometer. Parallel factor analysis (PARAFAC) was performed to deconvolute EEMs using the *drEEM* toolbox (v. 0.6.5)[2] in *MATLAB R2019a*. Four PARAFAC models (3-6 components) were evaluated using non-negativity constraints on all modes with 50 iterations and a convergence criterion of 10^{-10} to locate the minimum sum of squared errors.[3] Prior to PARAFAC modeling, raw EEMs were trimmed to the excitation wavelength range of 240 to 450 nm and the emission wavelength range of 248.242 to 598.548 nm, smoothed following the removal of primary and secondary Rayleigh and Raman scatter, and normalized to minimize the concentration-dependent co-linearity.[2] Upon examination of the core consistencies, sum of squared errors, and $S_4C_6T_3$ split-half validations (**Figure S1**),[2] a 4-component model was identified as the most appropriate model that explained 99.3% of the measured spectral variation across reverse-normalized EEMs (**Figures S2-S3**). Following the model validation, the true scores were converted to the maximum fluorescence intensities in water Raman unit (R.U.),[2] and the excitation and emission wavelengths of each component (**Figure S4**) were queried through the *OpenFluor* database[4] using a Tucker's congruence coefficient criterion of 0.95. Four PARAFAC components were operationally defined as component 1 (C1), component 2 (C2), component 3 (C3), and component 4 (C4), respectively. C2 and C3 had the highest number of matches in the *OpenFluor* database at the time of access (August 2024), followed by C1 and C4, respectively. C1 is a humic-like component (low aromaticity, low molecular weight),[5] C2 is a microbial humic-like component (aliphatic, low molecular weight),[5, 6] C3 is a tryptophan-like component (aliphatic, low molecular weight),[5-7] and C4 is a terrestrial humic-like component (high aromaticity, high molecular weight).[5] Selected physicochemical and optical properties of wastewater samples are summarized in **Table S3**.

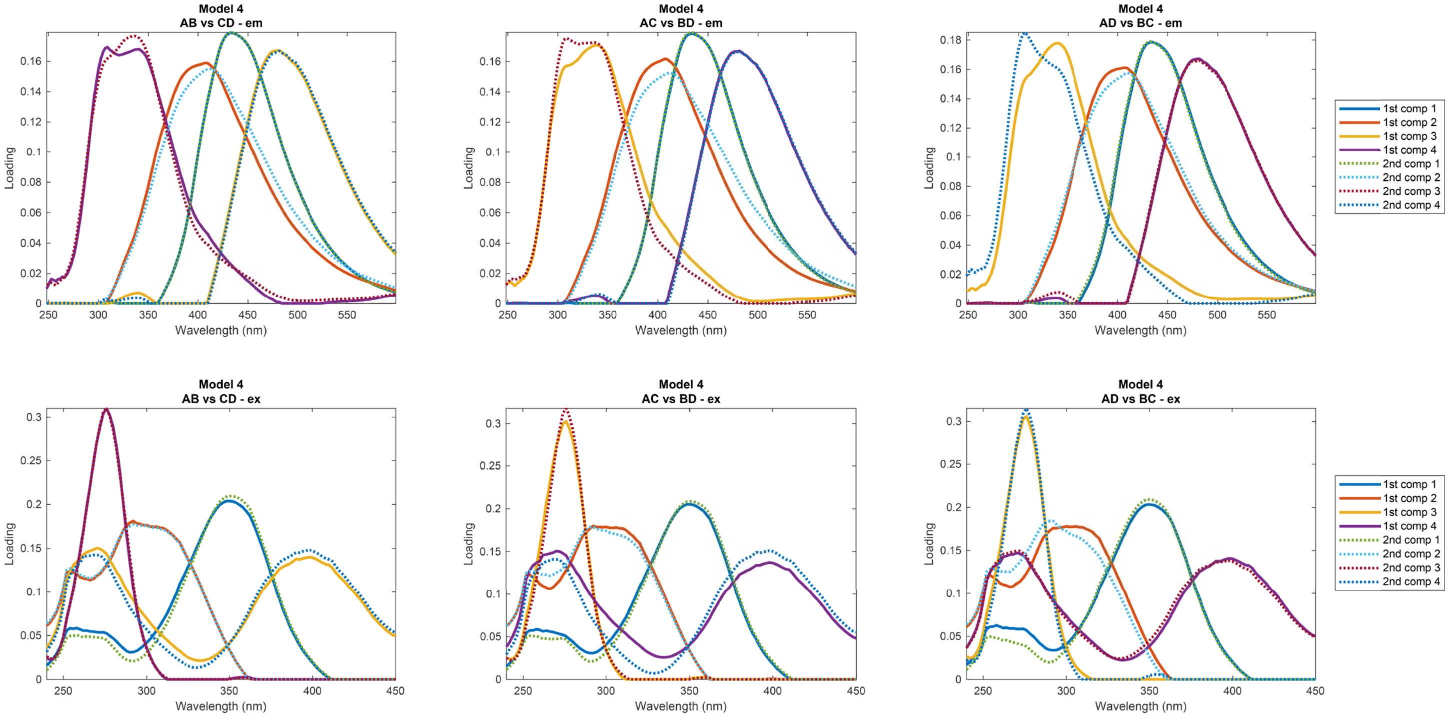

Figure S1. Split-half comparisons of the excitation and emission spectra from the 4-component PARAFAC model.

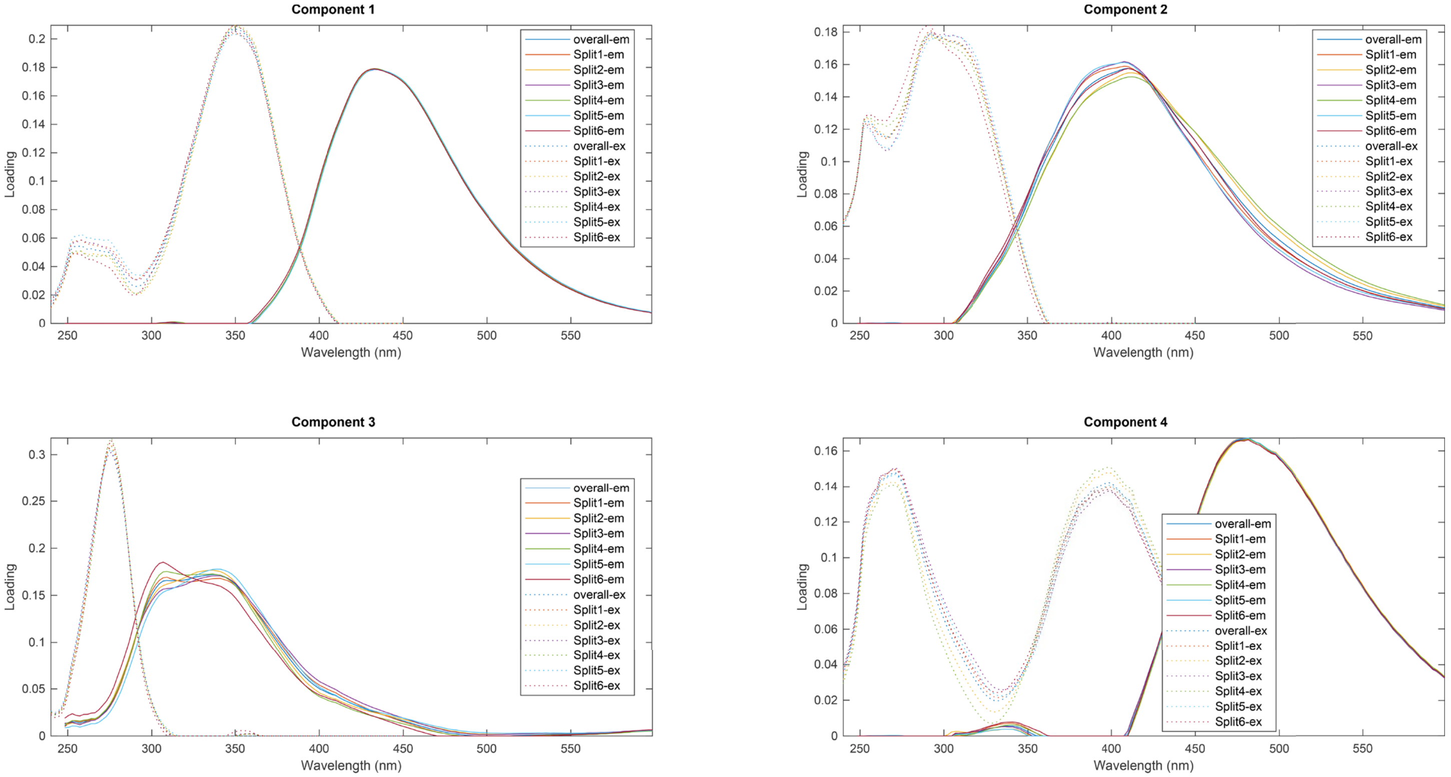

Figure S2. Overlaid spectral loadings of the 4-component PARAFAC model versus the overall model.

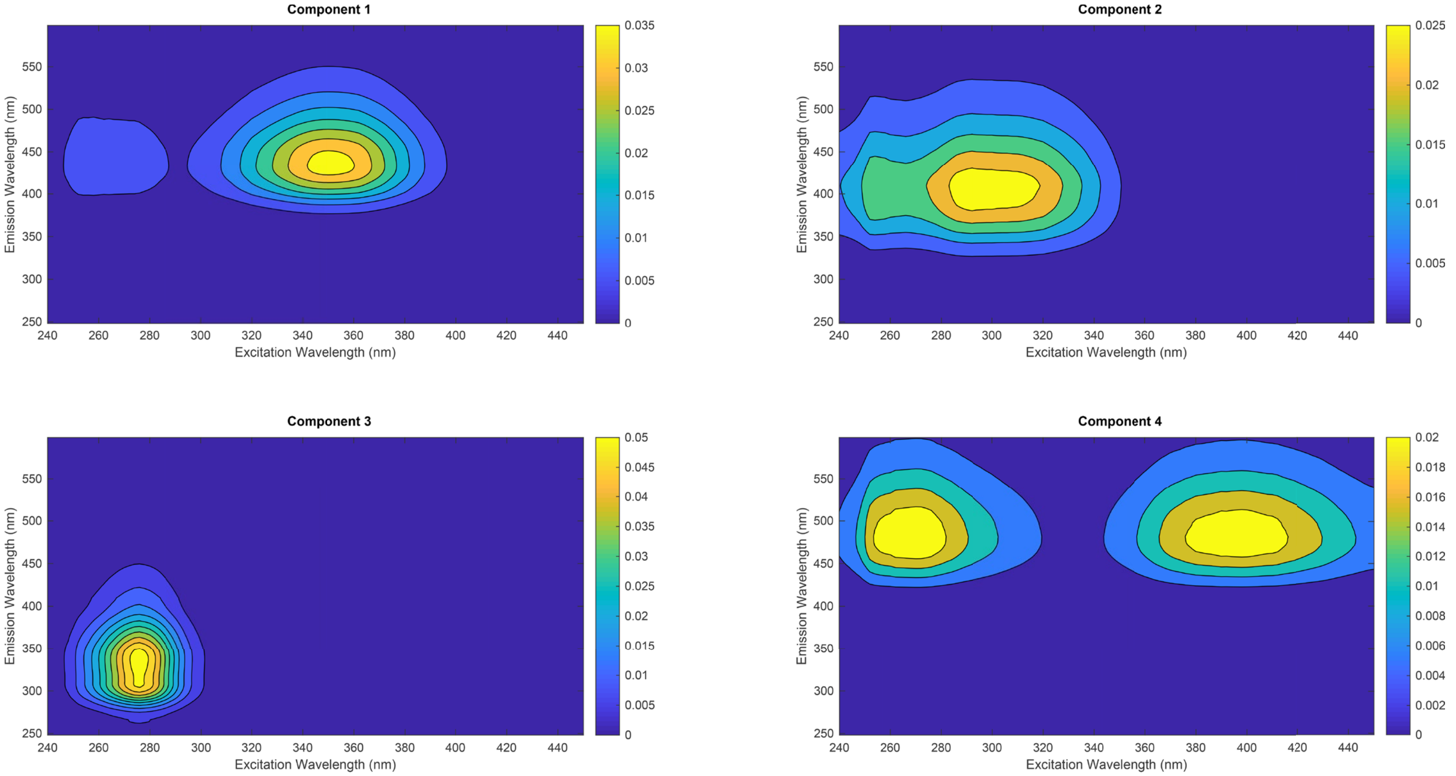

Figure S3. Contour plots of 4 fluorescent components validated for the 4-component PARAFAC model.

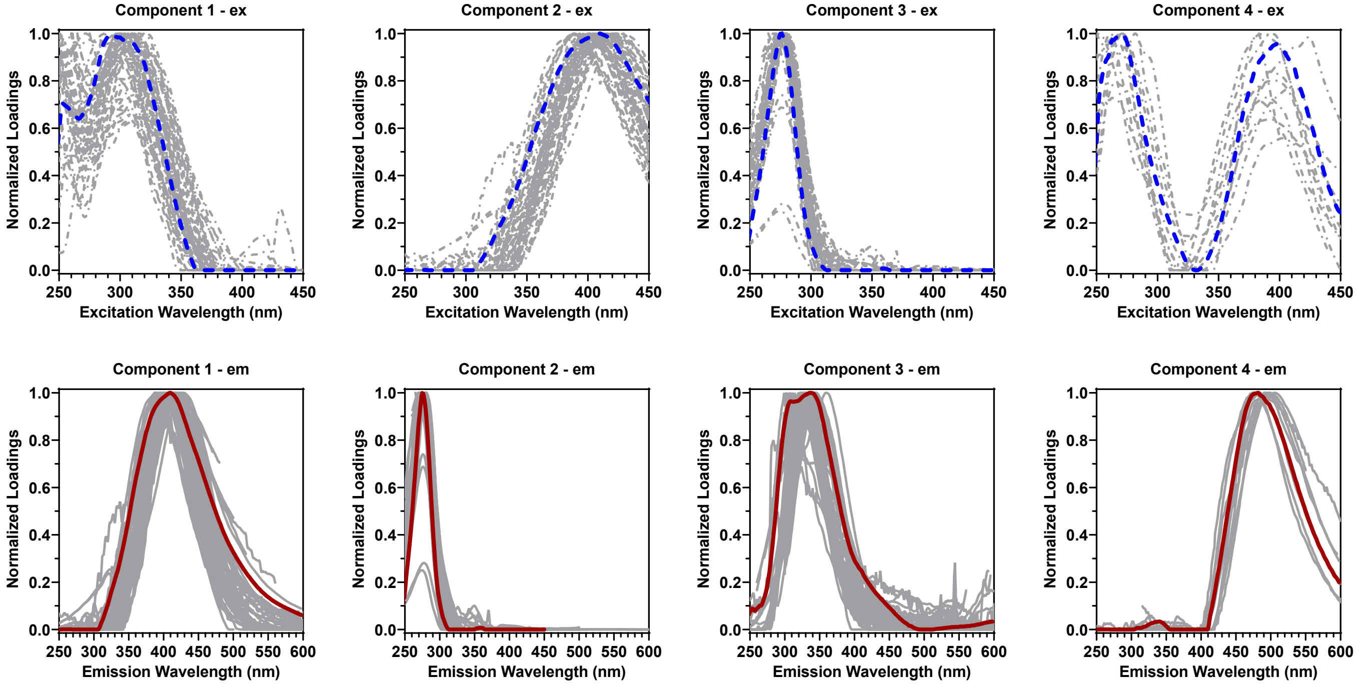

Figure S4. Spectral comparisons of the 4-component PARAFAC model with those published in the *OpenFluor* database.

**Table S3.** Physicochemical and optical properties of wastewater samples											
Sample ID	BOD_5 (mg/L)	TSS (mg/L)	NH_3-N (mg/L)	TKN (mg/L)	TP (mg/L)	C1 (R.U.)	C2 (R.U.)	C3 (R.U.)	C4 (R.U.)	Peak B (R.U.)	Peak T (R.U.)
WWTP A-1703-S1	46	167	10.0	12.2	2.3	3.32	3.08	2.23	1.66	1.89	3.10
WWTP A-1703-S2	38	127	8.1	10.1	1.8	4.30	3.09	1.28	1.56	1.76	2.17
WWTP A-1703-S3	-	-	-	-	-	5.55	4.49	3.31	2.17	2.91	4.30
WWTP A-1703-S4	-	12	6.2	9.1	1.5	4.07	3.65	1.71	3.01	2.75	2.54
WWTP A-1703-S5	-	5	0.5	2.4	0.2	1.33	1.10	0.90	0.49	1.37	1.76
WWTP A-1703-S6	-	-	-	-	-	1.40	1.27	0.29	0.61	1.12	1.31
WWTP A-1703-S7	3	5	0.5	1.8	0.1	1.45	1.21	0.94	0.55	1.09	1.56
WWTP A-1703-S8	-	-	-	-	-	11.22	9.20	6.72	4.68	5.27	8.01
WWTP A-1709-W1-S1	180	399	12.1	15.5	3.1	4.88	4.03	1.51	3.47	1.22	2.30
WWTP A-1709-W1-S2	48	42	-	-	2.1	5.38	4.20	2.39	2.21	1.14	1.70
WWTP A-1709-W1-S3	-	-	-	-	-	7.36	5.89	3.76	3.20	1.83	3.18
WWTP A-1709-W1-S4	-	17	8.2	12.8	1.7	6.56	4.59	2.57	3.53	1.74	3.07
WWTP A-1709-W1-S5	-	10	0.5	3.7	0.3	3.08	2.13	1.04	1.26	0.97	1.85
WWTP A-1709-W1-S6	-	-	-	-	-	2.49	1.66	0.88	0.89	0.83	1.52
WWTP A-1709-W1-S7	3	5	0.4	3.0	0.1	1.94	1.33	0.95	0.72	0.71	1.16
WWTP A-1709-W1-S8	-	-	-	-	-	13.24	10.67	4.98	6.85	3.97	7.67
WWTP A-1709-W2-S1	110	292	12.5	14.9	2.6	6.18	3.89	1.71	3.37	1.65	2.47
WWTP A-1709-W2-S2	-	-	-	-	-	7.74	5.71	4.45	3.11	2.36	3.02
WWTP A-1709-W2-S3	-	13	0.5	2.4	0.4	4.93	2.98	1.99	1.70	1.32	1.65
WWTP A-1709-W2-S4	-	-	-	-	-	3.56	2.32	0.94	1.27	1.07	1.84
WWTP A-1709-W2-S5	3	5	0.4	1.8	0.1	2.51	1.61	0.82	0.82	0.92	1.40
WWTP A-1709-W2-S6	-	-	-	-	-	14.33	10.90	7.44	5.82	5.73	7.47
WWTP A-1709-W3-S1	160	350	12.6	15.7	2.8	5.16	3.02	2.09	2.45	1.95	2.97
WWTP A-1709-W3-S2	-	-	-	-	-	8.00	5.84	3.07	3.31	2.74	3.68
WWTP A-1709-W3-S3	-	7	0.5	3.1	0.3	4.40	2.84	1.91	1.51	1.59	2.02
WWTP A-1709-W3-S4	-	-	-	-	-	3.27	2.17	0.97	1.11	1.31	2.24
WWTP A-1709-W3-S5	3	5	0.5	1.4	0.1	2.26	1.50	0.88	0.70	1.12	1.80
WWTP A-1709-W3-S6	-	-	-	-	-	16.13	11.82	6.50	6.37	5.95	8.25
WWTP A-1710-W4-S1	130	297	12.1	14.2	2.7	3.48	3.13	1.33	1.53	1.21	2.13
WWTP A-1710-W4-S2	-	-	-	-	-	8.84	5.68	3.84	3.17	1.74	2.87
WWTP A-1710-W4-S3	-	14	0.5	2.1	0.3	5.46	3.92	1.60	2.02	0.84	1.13
WWTP A-1710-W4-S4	-	-	-	-	-	4.13	3.45	0.79	1.70	0.70	1.52
WWTP A-1710-W4-S5	3	5	0.4	1.5	0.1	2.76	2.19	0.68	0.94	0.58	1.08
WWTP A-1710-W4-S6	-	-	-	-	-	21.81	16.63	6.42	9.22	4.09	6.28
WWTP A-1710-W5-S1	72	255	11.7	13.2	2.3	4.32	3.60	1.89	1.58	1.64	2.81
WWTP A-1710-W5-S2	-	-	-	-	-	8.60	6.17	2.77	3.17	2.43	3.75
WWTP A-1710-W5-S3	-	5	0.4	1.6	0.2	4.34	3.23	1.63	1.56	1.31	2.14
WWTP A-1710-W5-S4	-	-	-	-	-	3.16	2.56	0.81	1.23	1.09	2.48
WWTP A-1710-W5-S5	3	5	0.3	1.1	0.1	2.27	1.73	0.68	0.75	0.88	1.73

Table S3. Physicochemical and optical properties of wastewater samples (continued)											
Sample ID	BOD_5 (mg/L)	TSS (mg/L)	NH_3-N (mg/L)	TKN (mg/L)	TP (mg/L)	C1 (R.U.)	C2 (R.U.)	C3 (R.U.)	C4 (R.U.)	Peak B (R.U.)	Peak T (R.U.)
WWTP A-1710-W5-S6	-	-	-	-	-	18.92	14.72	7.16	7.50	5.02	7.67
WWTP B-1703-S1	70	94	12.9	15.3	2.7	2.31	1.66	2.28	0.56	1.96	2.53
WWTP B-1703-S2	-	-	-	-	-	1.53	0.94	1.41	0.30	1.84	2.46
WWTP B-1703-S3	-	-	-	-	-	4.24	2.22	2.36	1.10	3.28	4.14
WWTP B-1703-S4	-	-	-	-	-	2.73	0.98	0.77	2.08	3.11	3.69
WWTP B-1703-S5	-	-	-	-	-	2.38	0.93	0.54	0.51	1.78	1.78
WWTP B-1703-S6	-	-	0.5	2.3	-	1.96	0.77	0.41	0.38	1.68	1.99
WWTP B-1703-S7	4	5	0.4	2.3	0.1	2.52	0.87	0.44	0.42	1.64	2.02
WWTP B-1703-S8	-	-	-	-	-	6.84	5.57	4.11	2.73	4.17	5.11
WWTP B-1710-S1	93	137	16.4	18.5	3.2	6.65	3.38	2.04	1.86	1.63	3.13
WWTP B-1710-S2	-	-	-	-	-	6.72	5.78	2.46	3.83	2.89	4.96
WWTP B-1710-S3	-	-	0.5	2.1	-	7.23	4.35	1.17	2.93	1.52	3.14
WWTP B-1710-S4	3	6	0.4	1.0	0.1	8.66	4.71	1.00	2.87	1.26	2.15
WWTP B-1710-S5	-	-	-	-	-	10.02	7.83	3.40	4.82	3.87	4.66
WWTP C-1704-S1	170	220	18.2	28.5	4.9	5.04	3.01	5.11	2.09	4.13	7.09
WWTP C-1704-S2	105	160	-	-	1.6	5.86	3.78	5.88	1.95	4.00	5.91
WWTP C-1704-S3	-	-	-	-	-	6.44	4.26	8.84	2.77	5.97	8.62
WWTP C-1704-S4	3	5	-	-	-	5.95	3.65	7.65	2.22	5.83	8.83
WWTP C-1704-S5	-	-	0.5	13.9	-	3.00	2.68	7.31	1.46	5.43	8.08
WWTP C-1704-S6	3	5	0.4	13.5	0.5	3.00	2.66	6.89	1.38	5.36	7.64
WWTP C-1704-S7	-	-	-	-	-	7.64	6.08	9.75	2.79	8.66	12.05
WWTP C-1709-S1	230	435	19.7	29.4	5.5	7.08	5.22	7.05	3.34	4.96	8.43
WWTP C-1709-S2	-	-	-	-	-	9.20	7.32	8.33	3.98	7.30	9.02
WWTP C-1709-S3	-	-	0.6	15.2	-	6.83	4.28	7.87	2.69	6.82	9.70
WWTP C-1709-S4	3	5	0.3	11.1	0.4	6.59	6.26	6.74	4.07	5.88	8.32
WWTP C-1709-S5	-	-	-	-	-	10.93	8.18	11.22	5.04	10.02	13.72
WWTP D-1703-S1	120	158	12.4	16.5	2.9	4.78	2.99	3.20	1.59	3.26	4.85
WWTP D-1703-S2	-	-	-	-	-	7.27	5.20	4.86	2.87	3.81	4.08
WWTP D-1703-S3	-	-	-	-	-	6.45	4.23	3.10	2.41	3.25	3.21
WWTP D-1703-S4	-	-	0.5	4.1	-	5.25	2.72	1.55	1.35	2.99	4.10
WWTP D-1703-S5	16	7	0.4	4.0	0.2	5.19	2.63	1.50	1.30	2.93	3.89
WWTP D-1703-S6	-	-	-	-	-	8.87	7.29	6.60	4.18	6.18	7.56
WWTP D-1709-S1	140	183	21.8	26.1	5.1	7.72	4.20	2.06	2.43	1.70	3.25
WWTP D-1709-S2	-	-	-	-	-	9.03	5.33	4.47	2.77	2.64	5.02
WWTP D-1709-S3	-	-	0.5	6.5	-	7.21	4.64	2.82	2.45	1.88	3.60
WWTP D-1709-S4	5	5	0.3	4.5	0.2	7.21	4.56	1.41	2.60	1.39	2.43
WWTP D-1709-S5	-	-	-	-	-	10.37	8.62	6.91	5.13	4.28	7.96
WWTP E-1704-S1	81	122	10.3	14.8	2.9	3.46	1.67	1.64	0.79	2.11	2.24
WWTP E-1704-S2	-	-	-	-	-	5.88	2.45	4.20	2.89	3.05	3.99

Table S3. Physicochemical and optical properties of wastewater samples (continued)

Sample ID	BOD_5 (mg/L)	TSS (mg/L)	NH_3-N (mg/L)	TKN (mg/L)	TP (mg/L)	C1 (R.U.)	C2 (R.U.)	C3 (R.U.)	C4 (R.U.)	Peak B (R.U.)	Peak T (R.U.)
WWTP E-1704-S3	-	-	0.4	7.4	-	4.40	3.08	2.32	2.44	2.91	3.03
WWTP E-1704-S4	7	10	0.4	7.2	0.1	3.65	3.03	2.36	1.66	2.85	3.26
WWTP E-1704-S5	-	-	-	-	-	7.29	6.52	6.71	4.12	4.85	5.63
WWTP E-1709-S1	91	113	23.8	29.1	5.1	8.12	2.79	1.85	1.56	1.98	2.67
WWTP E-1709-S2	-	-	0.5	9.5	-	9.64	4.42	4.73	2.59	2.83	5.34
WWTP E-1709-S3	3	5	0.3	6.7	0.2	7.48	6.08	2.70	3.60	2.45	3.81
WWTP E-1709-S4	-	-	-	-	-	12.37	8.32	5.95	5.97	4.31	7.91
WWTP F-1703-S1	75	115	13.0	16.9	3.2	3.90	3.48	3.41	1.17	3.78	4.85
WWTP F-1703-S2	-	-	-	-	-	2.97	1.75	1.89	0.72	3.46	4.40
WWTP F-1703-S3	-	-	-	-	-	1.45	0.76	0.52	0.39	2.04	3.37
WWTP F-1703-S4	-	-	0.3	1.4	-	1.02	0.51	0.22	0.26	1.25	2.05
WWTP F-1703-S5	-	-	-	-	-	1.01	0.54	0.24	0.28	1.17	2.05
WWTP F-1703-S6	3	5	0.3	1.3	0.1	1.09	0.58	0.25	0.29	1.15	1.97
WWTP F-1703-S7	-	-	-	-	-	1.04	0.53	0.25	0.27	1.11	2.03
WWTP F-1710-S1	160	207	25.3	31.3	6.0	7.88	5.30	4.88	2.19	3.74	6.81
WWTP F-1710-S2	-	-	11.8	17.2	2.1	4.89	2.78	1.39	1.34	1.25	2.36
WWTP F-1710-S3	-	-	0.4	4.8	-	3.47	1.90	0.55	1.05	0.83	2.00
WWTP F-1710-S4	3	5	0.3	2.4	0.1	1.44	1.05	0.41	0.55	0.71	1.33
WWTP G-1708-S1	127	179	13.4	23.7	4.1	4.38	2.67	5.25	1.19	4.00	5.86
WWTP G-1708-S2	-	-	-	-	-	5.91	2.75	7.70	2.41	5.70	8.17
WWTP G-1708-S3	-	-	0.5	10.2	-	3.35	2.21	4.64	1.80	3.59	6.28
WWTP G-1708-S4	4	5	0.3	4.8	0.4	2.69	2.09	3.07	1.05	2.37	4.67
WWTP H-1708-S1	180	286	17.4	29.8	5.3	4.39	2.82	7.21	2.52	6.19	7.87
WWTP H-1708-S2	-	-	-	-	-	3.29	1.72	5.42	1.24	5.05	6.91
WWTP H-1708-S3	-	-	0.4	7.8	-	2.85	1.71	4.54	1.02	4.34	6.00
WWTP H-1708-S4	3	5	0.3	6.7	0.3	1.51	1.33	4.19	0.42	3.78	6.24

BOD_5 = 5-day biochemical oxygen demand; TSS = total suspended solids; NH_3-N = ammonia nitrogen; TKN = total Kjeldahl nitrogen; TP = total phosphorus; C1 = the maximum fluorescence intensity of the humic-like PARAFAC component; C2 = the maximum fluorescence intensity of the microbial humic-like PARAFAC component; C3 = the maximum fluorescence intensity of the tryptophan-like PARAFAC component; C4 = the maximum fluorescence intensity of the terrestrial humic-like PARAFAC component; Peak B = the abundance of the tyrosine-like organic matter fraction; Peak T = the abundance of the tryptophan-like organic matter fraction.

4. LC-HRMS instrument settings and method parameters

Table S4. LC-HRMS instrument settings

LC TriPlus RSH Autosampler and Liquid Handling System		
Injection Volume (μL)	20	
Bottom Sense Sample Vial	False	
Pre-Clean with Solvent 1	3	
Pre-Clean with Solvent 2	3	
Pre-Clean with Sample	0	
Sample Aspirate Flow Rate (μL/s)	10	
Filling Strokes	2	
Pullup Delay (ms)	500	
Inject Sample Flow Rate (μL/s)	5	
Air Volume (μL)	0	
Post-Clean with Solvent 1	3	
Post-Clean with Solvent 2	3	
Clean Valve with Solvent 1 (μL)	100	
Clean Valve with Solvent 2 (μL)	100	
Sample Vial Depth (mm)	30.0	
Height from Bottom of Sample Vial (mm)	1	
Clean Valve Flow Rate (μL/s)	100	
Wash Volume (%)	70	
Look Ahead	False	
Get Ready Delay (s)	5.0	
Injection Tool	LS1	
Wait For Sample Temperature	False	
sampleTemperature (°C)	4.0	
standbyTemperature (°C)	4.0	
Look Ahead Delay (min)	0.0	
Vanquish Horizon UHPLC System		
Flow Rate: 0.2 mL/min<br>Time (min)	Mobile Phase A (H_2O + 0.1% FA)	Mobile Phase B (MeOH + 0.1% FA)
0.0	95%	95%
4.0	95%	95%
7.5	90%	10%
9.5	80%	20%
14.0	20%	80%
15.0	10%	9%
20.0	10%	90%
20.5	95%	5%
26.5	95%	5%
Orbitrap Exploris 240 MS System		
Application Mode: Small Molecule<br>Method Duration (min): 26.5	Positive H-ESI	Negative H-ESI
	Ion Source	
Spray Voltage (V) | Static	3,400	2,000
Sheath Gas (Arb) | Static	30	30
Auxiliary Gas (Arb) | Static	5	5
Sweep Gas (Arb) | Static	0	0
Ion Transfer Tube Temperature (°C)	320	320
Vaporizer Temperature (°C)	275	275

**Table S4.** LC-HRMS instrument settings (continued)		
LC TriPlus RSH Autosampler and Liquid Handling System		
	MS Global Settings	
Infusion Mode	Liquid Chromatography	Liquid Chromatography
Expected LC Peak Width (s)	3	3
Mild Trapping	False	False
Default Charge State	1	1
Enable Xcalibur AcquireX Method Modifications	False	False
Internal Mass Calibration	EASY-IC^{TM}	EASY-IC^{TM}
Mode	Run Start	Run Start
	Divert Valve A	
Time (min)	Position	Position
0	1 – 6	1 – 6
0.5	1 – 2	1 – 2
30	1 – 6	1 – 6
	Full Scan	
Start Time (min)	0.5	0.5
End Time (min)	26.5	26.5
Orbitrap Resolution FWHM at m/z 200	60,000	60,000
Scan Range (m/z)	50 – 1000	50 – 1000
RF Lens (%)	70	70
AGC Target	Standard	Standard
Maximum Injection Time Mode	Auto	Auto
Microscans	1	1
Data Type	Profile	Profile
Polarity	Positive	Negative
Source Fragmentation	Disabled	Disabled
	Targeted Mass	
Mass List Type	m/z	m/z
Time Mode	Retention Time Window	Retention Time Window
Include Intensity Threshold	False	False
Mass Tolerance (ppm)	5	5
Set Collision Energy per Compound	False	False
Perform Dependent Scan on Most Intense Ion if No Targets are Found	False	False
	Data Dependent ddMS^2 Scan	
Data Dependent Mode	Number of Scans	Number of Scans
Number of Dependent Scans	10	10
Multiplex Ions	False	False
Isolation Window (m/z)	2	2
Isolation Offset	Off	Off
Collision Energy Type	Normalized	Normalized
HCD Collision Energies (%)	15, 30, 45, 60	15, 30, 45, 60
Orbitrap Resolution FWHM at m/z 200	15,000	15,000
Scan Range Mode	Auto	Auto
AGC Target	Standard	Standard
Maximum Injection Time Mode	Auto	Auto
Microscans	1	1
Data Type	Profile	Profile

**Table S5.** LC-HRMS method parameters for *N*-nitrosamines and isotope-labeled internal standards							
Compound	Acronym	Adduct	Scan Mode	Quantifier *m/z*	Qualifier *m/z*	Retention Time (min)	Category
N-Nitrosodimethylamine	NDMA	[M+H]+	Full	75.0555	58.0525	3.43	dialkyl
N-Nitrosomethylethylamine	NMEA	[M+H]+	Full	89.0711	61.0397	6.18	dialkyl
N-Nitrosodiethylamine	NDEA	[M+H]+	Full	103.0867	75.0554	12.32	dialkyl
N-Nitrosodipropylamine	NDPA	[M+H]+	Full	131.1179	89.0710	16.04	dialkyl
N-Nitrosodibutylamine	NDBA	[M+H]+	Full	159.1492	103.0867	17.20	dialkyl
N-Nitrosopyrrolidine	NPYR	[M+H]+	Full	101.0710	55.0543	7.52	cyclic
N-Nitrosopiperidine	NPIP	[M+H]+	Full	115.0869	69.0699	13.87	cyclic
N-Nitrosomorpholine	NMOR	[M+H]+	Full	117.0659	87.0679	5.79	heterocyclic
N-Nitrosodiphenylamine	NDPhA	[M+H]+	Full	199.0866	169.0885	17.72	diaryl
N-Nitrosodiethanolamine	NDELA	[M+H]+	Full	135.0764	84.9597	2.61	dialkyl
N-Nitrososarcosine	NSAR	[M+H]+	Full	119.0451	44.0495	3.04	dialkyl
N-Nitrosoproline	NPRO	[M+H]+	Full	145.0608	70.0652	5.43	cyclic
N-Nitrosopipecolic Acid	NPIC	[M+H]+	Full	159.0764	51.9400	13.66	cyclic
N-Nitrosohydroxyproline	NHPRO	[M+H]+	Full	161.0557	68.0496	2.38	cyclic
N-Nitrosothiazolidine-4-Carboxylic Acid	NTCA	[M+H]+	Full	163.0173	133.9660	6.52	heterocyclic
N-Nitroso-2-Methylthiazolidine 4-Carboxylic Acid	NMTCA	[M+H]+	Full	177.0328	147.9656	13.27	heterocyclic
N-Nitrosonornicotine	NNN	[M+H]+	Full	178.0975	148.0996	5.74	cyclic
N-Nitrosoanatabine	NAT	[M+H]+	Full	190.0975	160.0995	11.46	cyclic
N-Nitrosoanabasine	NAB	[M+H]+	Full	192.1131	162.1153	11.93	cyclic
4-(Methylnitrosamino)-1-(3-Pyridyl)-1-Butanone	NNK	[M+H]+	Full	208.1081	122.0601	14.28	dialkyl
4-(Methylnitrosamino)-1-(3-Pyridyl)-1-Butanol	NNAL	[M+H]+	Full	210.1237	180.1259	4.90	dialkyl
N-Nitrosomethylisopropylamine	NMIPA	[M+H]+	Full	103.0867	61.0397	11.60	dialkyl
N-Nitrosoethylpropylamine	NEPA	[M+H]+	Full	117.1023	116.0706	17.93	dialkyl
N-Nitrosomethylbutylamine	NMBA	[M+H]+	Full	117.1023	61.0397	15.65	dialkyl
N-Nitrosomethylisobutylamine	NMIBA	[M+H]+	Full	117.1023	75.0555	14.98	dialkyl
N-Nitroso-*tert*-Butylmethylamine	NTBMA	[M+H]+	Full	117.1023	57.0698	14.42	dialkyl
N-Nitrosomethylamylamine	NMAA	[M+H]+	Full	131.1184	71.0855	16.32	dialkyl
N-Nitrosodiisopropylamine	NDIPA	[M+H]+	Full	131.1184	89.0709	15.64	dialkyl
N-Nitroso-*tert*-Butylethylamine	NTBEA	[M+H]+	Full	131.1179	75.0555	15.98	dialkyl
N-Nitrosomethyl-*N,N*-Dimethylethylamine	NMDEA	[M+H]+	Full	132.1131	58.0653	2.08	dialkyl
N-Nitrosodiisobutylamine	NDIBA	[M+H]+	Full	159.1497	57.0700	17.14	dialkyl
N-Nitrosodiamylamine	NDAA	[M+H]+	Full	187.1810	71.0857	17.93	dialkyl
N-Nitrosoethylbenzylamine	NEBzA	[M+H]+	Full	165.1023	91.0544	16.63	dialkyl
N-Nitrosodibenzylamine	NDBzA	[M+H]+	Full	227.1179	91.0544	17.93	dialkyl
1-Nitroso-4-Methylpiperidine	NMPIP	[M+H]+	Full	129.1023	69.0700	15.64	cyclic
N-Nitroso-2-Pyrrolidinmethanol	NPYRM	[M+H]+	Full	138.0662	108.0685	5.80	cyclic
N-Nitrosopiperazine	NPPZ	[M+H]+	Full	116.0819	86.0840	1.96	heterocyclic
1-Nitroso-4-Phenylpiperazine	NPhPPZ	[M+H]+	Full	192.1131	162.1155	15.64	heterocyclic
N-Nitrosoindoline	NIND	[M+H]+	Full	149.0708	91.0545	16.48	cyclic alkylaryl

**Table S5.** LC-HRMS method parameters for *N*-nitrosamines and isotope-labeled internal standards (continued)							
Compound	Acronym	Adduct	Scan Mode	Quantifier *m/z*	Qualifier *m/z*	Retention Time (min)	Category
1-Nitroso-1,2,3,4-Tetrahydroquinoline	NTHQ	[M+H]+	Full	163.0866	105.0575	17.20	cyclic alkylaryl
N-Nitrosomethylcyclohexylamine	NMChA	[M+H]+	Full	143.1179	61.0398	16.30	alkylcycloalkyl
N-Nitrosodicyclohexylamine	NDChA	[M+H]+	Full	211.1805	83.0857	18.17	dicycloalkyl
N-Nitrosomethylphenylamine	NMPhA	[M+H]+	Full	137.0709	107.0732	16.16	alkylaryl
N-Nitrosoethylphenylamine	NEPhA	[M+H]+	Full	151.0866	105.0450	16.69	alkylaryl
N-Nitroso-*tert*-Butylphenylamine	NTBPhA	[M+H]+	Full	179.1184	93.0575	16.68	alkylaryl
N-Nitroso-4-Methylaminopyridine	NMAPY	[M+H]+	Full	138.0662	108.0685	4.75	alkylheteroaryl
N-Nitrososimazine	NSIM	[M+H]+	Full	231.0756	186.0545	17.23	alkylheteroaryl
N-Nitrosoatrazine	NATR	[M+H]+	Full	245.0912	215.0939	17.58	alkylheteroaryl
N-Nitrosoglyphosate	NGLP	[M+H]+	Full	199.0115	169.0890	1.34	dialkyl
N-Nitrosobetahistine	NBTH	[M+H]+	Full	166.0975	93.0575	3.86	dialkyl
N-Nitrosoephedrine	NEPD	[M+H]+	Full	195.1128	61.0398	15.71	dialkyl
N-Nitrosofenfluramine	NFFA	[M+H]+	Full	261.1214	159.0420	17.40	dialkyl
N-Nitrosonortriptyline	NNTP	[M+H]+	Full	293.1648	233.1333	18.79	dialkyl
N-Nitrosodesipramine	NDSP	[M+H]+	Full	296.1757	266.1784	18.89	dialkyl
N-Nitrosofluoxetine	NFXT	[M+H]+	Full	339.1315	177.1027	18.26	dialkyl
N-Nitrosopropranolol	NPPN	[M+H]+	Full	289.1547	259.1571	18.12	dialkyl
N-Nitrosoatenolol	NATN	[M+H]+	Full	296.1605	266.1793	15.33	dialkyl
N-Nitrosometoprolol	NMTP	[M+H]+	Full	297.1809	223.1571	16.85	dialkyl
N-Nitrososotalol	NSOT	[M+H]+	Full	302.1169	197.0510	15.41	dialkyl
N-Nitrosonadolol	NNAD	[M+H]+	Full	339.1915	309.1946	16.34	dialkyl
N-Nitrosonebivolol	NNBV	[M+H]+	Full	435.1726	405.1761	18.50	dialkyl
N-Nitrosodesloratadine	NDLT	[M+H]+	Full	340.1211	310.1241	17.94	cyclic
N-Nitrosonorfloxacin	NNFX	[M+H]+	Full	349.1307	319.1340	18.55	heterocyclic
N-Nitrosociprofloxacin	NCFX	[M+H]+	Full	361.1307	245.0783	19.20	heterocyclic
N-Nitrosodiclofenac	NDCF	[M-H]-	Full	322.9998	282.9832	17.26	diaryl
N-Nitrosoiminostilbene	NISB	[M+H]+	Full	223.0866	193.0892	17.69	cyclic diaryl
N-Nitrosoiminodibenzyl	NIDB	[M+H]+	Full	225.1022	195.1046	17.96	cyclic diaryl
N-Nitrosodimethyl-d_6-Amine	NDMA-d_6	[M+H]+	Full	81.0932	64.0904	3.20	ILIS
N-Nitrosodiethyl-d_{10}-Amine	NDEA-d_{10}	[M+H]+	Full	113.1494	81.0932	12.00	ILIS
N-Nitrosodi-*n*-Propyl-d_{14}-Amine	NDPA-d_{14}	[M+H]+	Full	145.2058	97.1213	15.80	ILIS
N-Nitrosopyrrolidine-d_8	NPYR-d_8	[M+H]+	Full	109.1212	62.0830	6.80	ILIS
N-Nitrosomorpholine-d_8	NMOR-d_8	[M+H]+	Full	125.1161	95.1184	5.50	ILIS
N-Nitrosodiphenyl-2,2',4,4',6,6'-d_6-Amine	NDPhA-d_6	[M+H]+	Full	205.1239	175.1267	17.70	ILIS
N-Nitrosobis(2-Hydroxyethyl)-d_8-Amine	NDELA-d_8	[M+H]+	Full	143.1266	113.1285	2.40	ILIS
N-Methyl-d_3-*N*-Nitrosoglycine	NSAR-d_3	[M+H]+	Full	122.0640	104.9636	2.90	ILIS
ILIS = Isotope-labeled internal standard.							

5. Nontarget screening workflow settings

**Table S6.** *Compound Discoverer 3.3* workflow node settings	
Select Spectra	
1. Spectrum Properties Filter	
Lower RT Limit	1.0
Upper RT Limit	20.5
2. Scan Event Filters	
Polarity Mode	Any
Align Retention Times	
1. General Settings	
Alignment Model	Adaptive curve
Maximum Shift [min]	2
Mass Tolerance	5 ppm
Detection Compounds	
1. General Settings	
Mass Tolerance	5 ppm
Min. Peak Intensity	50000
Use Most Intense Isotope Only	True
2. Peak Detection	
Chromatographic S/N Threshold	1.5
Remove Baseline	False
3. Isotope Pattern Detection	
Group Isotopes for	Br; Cl
4. Compound Detection	
Ions	[M+H]+1; [M+Na]+1; [M-H]-1
Generate Expected Compounds	
1. Compound Selection	
Compounds	Precursors
2. Dealkylation	
Apply Dealkylation	False
Apply Dearylation	False
Max. # Steps	1
Min. Mass [Da]	200
3. Transformations	
Phase I	
Phase II	
Others	Nitrosation (H → N O)
Max. # Phase II	1
Max. # All Steps	3
4. Ionization	
Ions	[M+H]+1; [M-H]-1
Find Expected Compounds	
1. General Settings	
Mass Tolerance	5 ppm
Intensity Tolerance [%]	30
Intensity Threshold [%]	0.1
Min. # Isotopes	2
Use Most Intense Isotope Only	True
Min. Peak Intensity	50000
Average Peak Width [min]	0
2. Peak Detection	
Chromatographic S/N Threshold	1.5
Remove Baseline	True

**Table S6.** *Compound Discoverer 3.3* workflow node settings (continued)	
Merge Features	
1. Peak Consolidation	
Mass Tolerance	5 ppm
RT Tolerance [min]	0.1
Group Compounds	
1. General Settings	
Mass Tolerance	5 ppm
RT Tolerance [min]	0.1
Align Peaks	False
Preferred Ions	[M+H]+1; [M+Na]+1; [M-H]-1
Area Integration	Most Common Ion
2. Peak Rating Contributions	
Area Contribution	3
CV Contribution	10
FWHM to Base Contribution	5
Jaggedness Contribution	5
Modality Contribution	5
Zig-Zag Index Contribution	5
3. Peak Rating Filter	
Peak Rating Threshold	4
Number of Files	2
Group Expected Compounds	
1. General Settings	
RT Tolerance [min]	0.2
Align Peaks	False
Preferred Ions	[M+H]+1; [M-H]-1
Area Integration	Most Common Ion
2. Peak Rating Contributions	
Area Contribution	3
CV Contribution	10
FWHM to Base Contribution	5
Jaggedness Contribution	5
Modality Contribution	5
Zig-Zag Index Contribution	5
3. Peak Rating Filter	
Peak Rating Threshold	4
Number of Files	2
FISh Scoring	
1. General Settings	
Annotate Full Tree	True
Match Transformations	True
S/N Threshold	3
High Acc. Mass Tolerance	5 ppm
Low Acc. Mass Tolerance	0.5 Da
2. Fragment Predicting Settings	
Use General Rules	True
Use Libraries	True
Max. Depth	5
Automatic Cleavage	True
Min. Fragment m/z	50
Fill Gaps	
1. General Settings	
Mass Tolerance	5 ppm
S/N Threshold	1.5

**Table S6.** *Compound Discoverer 3.3* workflow node settings (continued)	
Apply SERRF QC Correction	
1. General Settings	
Min. QC Coverage [%]	50
Max. QC Area RSD [%]	30
Max. Corrected QC Area RSD [%]	25
# Batches	2
Interpolate Gap-filled QC Areas	True
Mark Background Compounds	
1. General Settings	
Max. Sample/Blank	5
Max. Blank/Sample	0
Hide Background	True
Normalize Areas	
1. General Settings	
Normalization Type	Constant Median
Exclude Blanks	True
Predict Compositions	
1. Prediction Settings	
Mass Tolerance	5 ppm
Min. Element Counts	C2 H6 N2 O
Max. Element Counts	C50 H100 Br Cl3 F3 N10 O15 P S
Min. RDBE	0
Max. RDBE	40
Min. H/C	0.1
Max. H/C	3.5
Max. # Candidates	10
2. Pattern Matching	
Intensity Tolerance [%]	30
Intensity Threshold [%]	0.1
S/N Threshold	3
Use Dynamic Recalibration	True
3. Fragments Matching	
Use Fragments Matching	True
Mass Tolerance	10 ppm
S/N Threshold	3
Search mzVault	
1. Search Settings	
mzVault Library	\MassBank.db
Compound Classes	All
Match Ion Activation Type	False
Match Ion Activation Energy	Match with Tolerance
Ion Activation Energy Tolerance	20
Match Ionization Method	False
Apply Intensity Threshold	True
Precursor Mass Tolerance	5 ppm
Match Analyzer Type	False
Search Algorithm	HighChem HighRes
Match Factor Threshold	80
RT Tolerance [min]	2
Use Retention Time	False
Search mzCloud	
1. General Settings	
Compound Classes	All
Library	Autoprocessed; Reference
Search MSn Tree	False

**Table S6.** *Compound Discoverer 3.3* workflow node settings (continued)	
Search mzCloud (continued)	
2. DDA Search	
Identity Search	HighChem HighRes
Match Ion Activation Type	False
Match Ion Activation Energy	Match with Tolerance
Ion Activation Energy Tolerance	20
Apply Intensity Threshold	True
Similarity Search	None
Match Factor Threshold	80
3. DIA Search	
Use DIA Scans for Search	False
Max. Isolation Width [Da]	500
Match Ion Activation Type	False
Match Ion Activation Energy	Any
Ion Activation Energy Tolerance	100
Apply Intensity Threshold	True
Match Factor Threshold	80
Assign Compound Annotations	
1. General Settings	
Mass Tolerance	5 ppm
2. Data Sources	
Data Source #1	mzCloud Search
Data Source #2	mzVault Search
Data Source #3	MassList Search
Data Source #4	ChemSpider Search
Data Source #5	Predicted Compositions
3. Scoring Rules	
Use mzLogic	True
Use Spectral Distance	True
SFit Threshold	90
SFit Range	20
4. Reprocessing	
Clear Names	False
Search Mass Lists	
1. Search Settings	
Mass Lists	\Import from csv\N-Nitroso_compound_database (Table S8)
Use Retention Time	True
RT Tolerance [min]	2
Mass Tolerance	5 ppm
Search ChemSpider	
1. Search Settings	
Database(s)	CAS Common Chemistry; DrugBank; EAWAG Biocatalysis/Biodegradation Database; ECHA; EPA DSSTox; Journal of Heterocyclic Chemistry; Lhasa Limited; Sigma-Aldrich
Search Mode	By Formula or Mass
Mass Tolerance	5 ppm
Max. # of Results per Compound	20
Max. # of Predicted Compositions to be Searched per Compound	3

**Table S6.** *Compound Discoverer 3.3* workflow node settings (continued)	
Calculate Mass Defect	
1. Mass Defect	
Fractional Mass	False
Standard Mass Defect	False
Relative Mass Defect	False
Kendrick Mass Defect	True
2. Kendrick Formula	
Formula 1	N O
Formula 2	C2 F4
Formula 3	C2 F3 O
Formula 4	C2 H4
Formula 5	C3 H6
Apply Spectral Distance	
1. Pattern Matching	
Mass Tolerance	5 ppm
Intensity Tolerance [%]	30
Intensity Threshold [%]	0.1
S/N Threshold	3
Use Dynamic Recalibration	True
Apply mzLogic	
1. Search Settings	
Max. # Compounds	0
Max. # mzCloud Similarity Results to consider per Compound	10
Match Factor Threshold	80
Search Neutral Losses	
1. General Settings	
Neutral Losses	NO (N O, 30.00)
High Acc. Mass Tolerance	10 ppm
Low Acc. Mass Tolerance	0.5 Da
S/N Threshold	3
Use DIA Scans for Search	False
Differential Analysis	
1. General Settings	
Log10 Transform Values	True
2. Peak Rating Contributions	
Update Peak Rating	True
Area Contribution	3
CV Contribution	10
FWHM to Base Contribution	5
Jaggedness Contribution	5
Modality Contribution	5
Zig-Zag Index Contribution	5
Descriptive Statistics	
Scripting Node	
1. Executable and Parameters	
Path to Executable	C:\Program Files\R\R-4.3.1\bin\Rscript.exe
Command Line Arguments	C:\Rscripts\Nitrosamines_NitrogenOxygen.R %NODEARGS%
Requested Tables and Columns	Compounds: Formula, Calc. MW, m/z; Predicted Compositions: Formula; ChemSpider Results: Formula; Mass List Search Results: Formula
Use R-Friendly Columns	False
Archive Datafiles	False

Table S6. *Compound Discoverer 3.3* workflow node settings (continued)

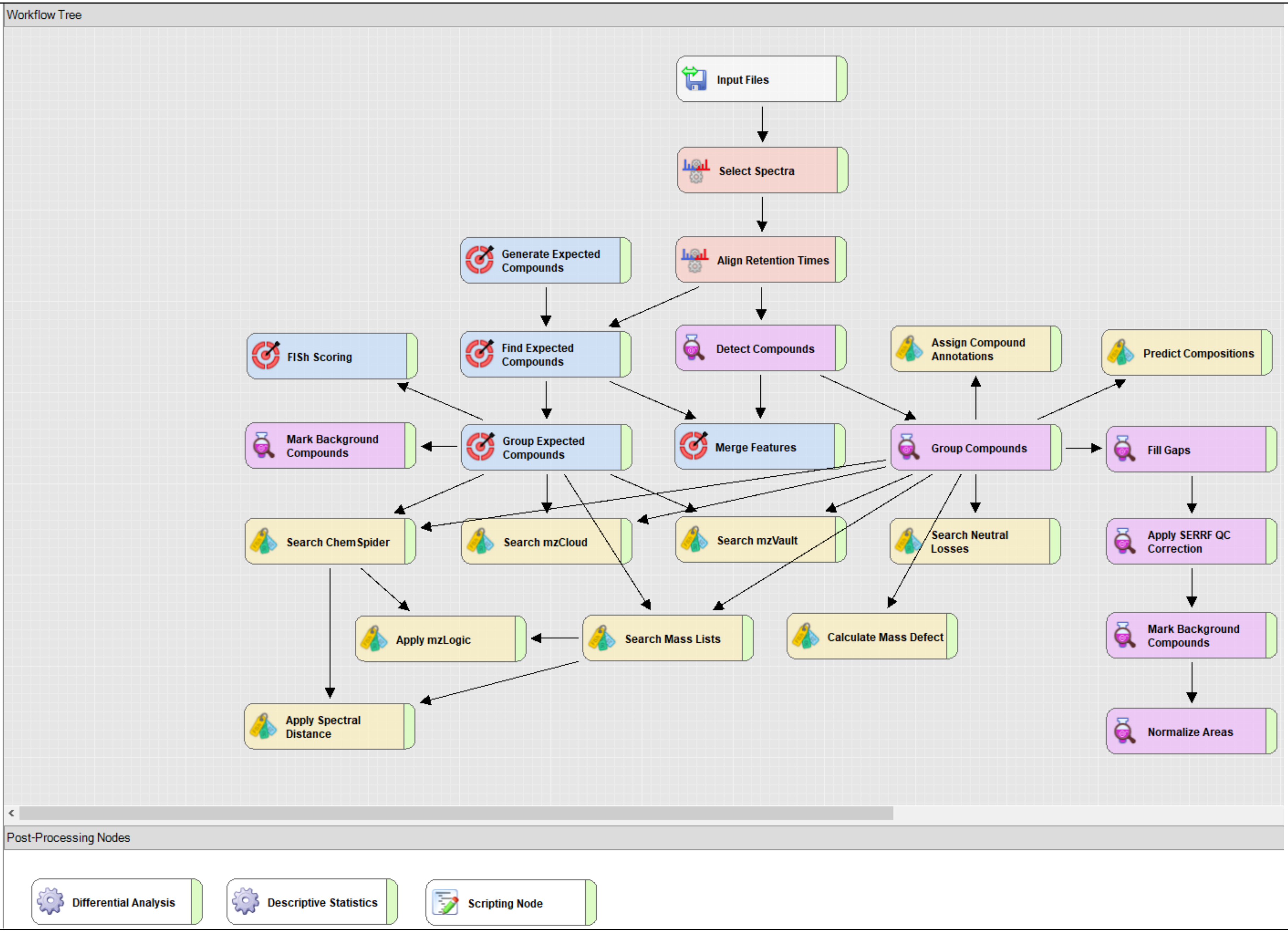

**Table S7.** *N*-Nitroso compound database imported into the *Search Mass Lists* node				
N-Nitroso Compound Name	PubChem CID	CAS	Molecular Formula	SMILES
N-Nitrosodimethylamine (NDMA)	6124	62-75-9	C2H6N2O	CN(C)N=O
N-Nitrosoazetidine	27135	15216-10-1	C3H6N2O	C1CN(C1)N=O
N-Nitrosomethylethylamine (NMEA)	25418	10595-95-6	C3H8N2O	CCN(C)N=O
N-Nitrosopyrrolidine (NPYR)	13591	930-55-2	C4H8N2O	C1CCN(C1)N=O
N-Methyl-*N*-nitrosocyclopropanamine	20196151	1936348-61-6	C4H8N2O	CN(C1CC1)N=O
N-Nitroso-1,3-oxazolidine	114957	39884-52-1	C3H6N2O2	C1COCN1N=O
1-Nitrosoazetidin-3-ol	85943953	664985-71-1	C3H6N2O2	C1C(CN1N=O)O
N-Nitrosodiethylamine (NDEA)	5921	55-18-5	C4H10N2O	CCN(CC)N=O
N-Nitrosomethylisopropylamine	92271	30533-08-5	C4H10N2O	CC(C)N(C)N=O
N-Nitrosomethylpropylamine	13545	924-46-9	C4H10N2O	CCCN(C)N=O
(2-Aminoethyl)(methyl)nitrosoamine	87375711	2680529-64-8	C3H9N3O	CN(CCN)N=O
N-Nitrosomethyl-(2-hydroxyethyl)amine	33646	26921-68-6	C3H8N2O2	CN(CCO)N=O
1-Nitroso-1,2,3,6-tetrahydropyridine	41470	55556-92-8	C5H8N2O	C1CN(CC=C1)N=O
3-Nitroso-3-azabicyclo[3.1.0]hexane	74025066	159683-62-2	C5H8N2O	C1C2C1CN(C2)N=O
3-[Methyl(nitroso)amino]propanenitrile	62163	60153-49-3	C4H7N3O	CN(CCC#N)N=O
N-Nitrosopiperidine (NPIP)	7526	100-75-4	C5H10N2O	C1CCN(CC1)N=O
N-Methyl-*N*-nitrosocyclobutanamine	155977985	2624140-70-9	C5H10N2O	CN(C1CCC1)N=O
N-Nitrosopiperazine	21845	5632-47-3	C4H9N3O	C1CN(CCN1)N=O
N-Nitrosomorpholine (NMOR)	6046	59-89-2	C4H8N2O2	C1COCCN1N=O
N-Nitroso-3-hydroxypyrrolidine	3034827	56222-35-6	C4H8N2O2	C1CN(CC1O)N=O
N-Nitrosomethyl(2-oxopropyl)amine	104910	55984-51-5	C4H8N2O2	CC(=O)CN(C)N=O
3-Nitrosotetrahydro-1,3-oxazine	93185	35627-29-3	C4H8N2O2	C1CN(COC1)N=O
N-Nitrosomethylbutylamine	23471	7068-83-9	C5H12N2O	CCCCN(C)N=O
N-Nitroso-*tert*-butylmethylamine	75630	2504-18-9	C5H12N2O	CC(C)(C)N(C)N=O
N-Nitrosoethylisopropylamine	27824	16339-04-1	C5H12N2O	CCN(C(C)C)N=O
N-Nitrosomethylisobutylamine	118679	34419-76-6	C5H12N2O	CC(C)CN(C)N=O
N-Nitrosoethylpropylamine	93087	25413-61-0	C5H12N2O	CCCN(CC)N=O
Methyl({2-[methyl(nitroso)amino]ethyl})amine	55285153	2375271-12-6	C4H11N3O	CNCCN(C)N=O
(2-Aminoethyl)(ethyl)nitrosoamine	165940518	2680539-27-7	C4H11N3O	CCN(CCN)N=O
N-Nitrososarcosine (NSAR)	25811	13256-22-9	C3H6N2O3	CN(CC(=O)O)N=O
(3*R*)-3-Fluoro-1-nitrosopyrrolidine	155943374	2613300-07-3	C4H7FN2O	C1CN(CC1F)N=O
(2-Methoxyethyl)(methyl)nitrosoamine	130736834	124485-85-4	C4H10N2O2	CN(CCOC)N=O
N-Nitrosoethylethanolamine	25742	13147-25-6	C4H10N2O2	CCN(CCO)N=O
2-[Methyl(nitroso)amino]propan-1-ol	87375177	121861-55-0	C4H10N2O2	CC(CO)N(C)N=O
3-[Methyl(nitroso)amino]propan-1-ol	125833	70415-59-7	C4H10N2O2	CN(CCCO)N=O
N-Nitrosomethyl-2-chloroethylamine	27829	16339-16-5	C3H7ClN2O	CN(CCCl)N=O
3,3-Difluoro-1-nitrosoazetidine	165905195	2688115-44-6	C3H4F2N2O	C1C(CN1N=O)(F)F
N-Nitrosodi(cyanomethyl)amine	27830	16339-18-7	C4H4N4O	C(C#N)N(CC#N)N=O
N-Nitroso-4-piperidone	41469	55556-91-7	C5H8N2O2	C1CN(CCC1=O)N=O
N-Nitrosohexamethyleneimine	13613	932-83-2	C6H12N2O	C1CCCN(CC1)N=O
1-Nitroso-4-methylpiperidine	27026	15104-03-7	C6H12N2O	CC1CCN(CC1)N=O

**Table S7.** *N*-Nitroso compound database imported into the *Search Mass Lists* node (continued)				
N-Nitroso Compound Name	PubChem CID	CAS	Molecular Formula	SMILES
N-Nitroso-*N*-(propan-2-yl)cyclopropanamine	144089489	2792201-70-6	C6H12N2O	CC(C)N(C1CC1)N=O
(2*S*)-2-Methyl-1-nitrosopiperidine	93193	36702-44-0	C6H12N2O	CC1CCCCN1N=O
2,5-Dimethyl-*N*-nitrosopyrrolidine	41467	55556-86-0	C6H12N2O	CC1CCC(N1N=O)C
4-Nitrosopiperazin-2-one	53769220	18907-82-9	C4H7N3O2	C1CN(CC(=O)N1)N=O
1-Methyl-4-nitrosopiperazine	27826	16339-07-4	C5H11N3O	CN1CCN(CC1)N=O
1-Nitrosoazetidine-3-carboxylic Acid	20203577	102624-97-5	C4H6N2O3	C1C(CN1N=O)C(=O)O
(1-Nitrosopyrrolidin-3-yl)methanol	75481122	1803598-92-6	C5H10N2O2	C1CN(CC1CO)N=O
3-Methoxy-1-nitrosopyrrolidine	12630787	61467-70-7	C5H10N2O2	COC1CCN(C1)N=O
1-Nitroso-4-piperidinol	41471	55556-93-9	C5H10N2O2	C1CN(CCC1O)N=O
N-Nitroso-2-pyrrolidinmethanol	45092519	68292-94-4	C5H10N2O2	C1CC(N(C1)N=O)CO
3-Methyl-4-nitrosomorpholine	92394	94123-29-2	C5H10N2O2	CC1COCCN1N=O
Nitroso-2-methylmorpholine	21116826	92071-38-0	C5H10N2O2	CC1CN(CCO1)N=O
N-Nitroso-3-hydroxypiperidine	41466	55556-85-9	C5H10N2O2	C1CC(CN(C1)N=O)O
N-Nitrosodipropylamine (NDPA)	12130	621-64-7	C6H14N2O	CCCN(CCC)N=O
N-Nitrosodiisopropylamine	11758	601-77-4	C6H14N2O	CC(C)N(C(C)C)N=O
N-Nitrosomethylamylamine	25805	13256-07-0	C6H14N2O	CCCCCN(C)N=O
N-Nitrosoethylbutylamine	20680	4549-44-4	C6H14N2O	CCCCN(CC)N=O
N-Nitroso-*tert*-butylethylamine	18835	3398-69-4	C6H14N2O	CCN(C(C)(C)C)N=O
N-Nitrosomethyl-*N,N*-dimethylethylamine	146842	23834-30-2	C5H13N3O	CN(C)CCN(C)N=O
N-Nitrosothiomorpholine	33535	26541-51-5	C4H8N2OS	C1CSCCN1N=O
N-Nitrosodiethanolamine (NDELA)	14223	1116-54-7	C4H10N2O3	C(CO)N(CCO)N=O
3,3-Difluoro-1-nitrosopyrrolidine	155973620	2648966-19-0	C4H6F2N2O	C1CN(CC1(F)F)N=O
N-Nitrosomethylphenylamine	11957	614-00-6	C7H8N2O	CN(C1=CC=CC=C1)N=O
N-Nitroso-4-methylaminopyridine	146271	16219-99-1	C6H7N3O	CN(C1=CC=NC=C1)N=O
2-Nitrosomethylaminopyridine	146270	16219-98-0	C6H7N3O	CN(C1=CC=CC=N1)N=O
N-Nitroso-*N*-methyl-3-aminopyridine	125780	69658-91-9	C6H7N3O	CN(C1=CN=CC=C1)N=O
(3a*R*,6a*S*)-2-Nitroso-octahydrocyclopenta[*c*]pyrrole	92974306	2445750-74-1	C7H12N2O	C1CC2CN(CC2C1)N=O
6-Nitroso-6-azaspiro[2.5]octane	165940972	2680528-32-7	C7H12N2O	C1CC12CCN(CC2)N=O
N-Nitrosomethylcyclohexylamine	21567	5432-28-0	C7H14N2O	CN(C1CCCCC1)N=O
N-Nitrosoheptamethyleneimine	30360	20917-49-1	C7H14N2O	C1CCCN(CCC1)N=O
4,4-Dimethyl-1-nitrosopiperidine	91618418	32062-81-0	C7H14N2O	CC1(CCN(CC1)N=O)C
2,6-Dimethyl-1-nitrosopiperidine	28717	17721-95-8	C7H14N2O	CC1CCCC(N1N=O)C
4-Nitrosopiperazine-1-carbaldehyde	151560737	2455384-08-2	C5H9N3O2	C1CN(CCN1C=O)N=O
1-Ethyl-4-nitrosopiperazine	12554624	65504-33-8	C6H13N3O	CCN1CCN(CC1)N=O
N-Methyl-*N’*-nitrosohomopiperazine	23461670	2731009-81-5	C6H13N3O	CN1CCCN(CC1)N=O
N-Methyl-*N*-nitroso-1,3,4-thiadiazol-2-amine	12379851	41148-18-9	C3H4N4OS	CN(C1=NN=CS1)N=O
N-Nitrosoproline	24141	7519-36-0	C5H8N2O3	C1CC(N(C1)N=O)C(=O)O
1-Nitrosopyrrolidine-3-carboxylic Acid	155943373	2613385-61-6	C5H8N2O3	C1CN(CC1C(=O)O)N=O
1,4-Dinitrosopiperazine	8819	140-79-4	C4H8N4O2	C1CN(CCN1N=O)N=O
(1-Nitrosopiperidin-4-yl)methanol	150090832	2416858-89-2	C6H12N2O2	C1CN(CCC1CO)N=O
(2*S*,6*R*)-2,6-Dimethyl-4-nitrosomorpholine	3035035	69091-16-3	C6H12N2O2	CC1CN(CC(O1)C)N=O

**Table S7.** *N*-Nitroso compound database imported into the *Search Mass Lists* node (continued)				
N-Nitroso Compound Name	PubChem CID	CAS	Molecular Formula	SMILES
(1-Nitrosopiperidin-2-yl)methanol	85917007	64605-07-8	C6H12N2O2	C1CCN(C(C1)CO)N=O
N-Nitroso-*N*-butyl-*N*-propylamine	32965	25413-64-3	C7H16N2O	CCCCN(CCC)N=O
N-Nitroso-*N*-*n*-butyl-*N*-isopropylamine	21404122	2680532-87-8	C7H16N2O	CCCCN(C(C)C)N=O
[2-(Dimethylamino)ethyl](ethyl)nitrosoamine	156620436	23843-00-7	C6H15N3O	CCN(CCN(C)C)N=O
Dimethyl({3-[methyl(nitroso)amino]propyl})amine	165599024	2731010-51-6	C6H15N3O	CN(C)CCCN(C)N=O
N-Nitroso-*N*-methyl-4-aminobutyric Acid	43659	61445-55-4	C5H10N2O3	CN(CCCC(=O)O)N=O
N-Nitroso-*N*-isopropylglycine	96156	6939-16-8	C5H10N2O3	CC(C)N(CC(=O)O)N=O
2-Methyl-3-(methyl(nitroso)amino)propanoic Acid	165905220	2624123-51-7	C5H10N2O3	CC(CN(C)N=O)C(=O)O
3-(Methyl(nitroso)amino)butanoic Acid	129829161	2624139-24-6	C5H10N2O3	CC(CC(=O)O)N(C)N=O
Methyl({2-[methyl(nitroso)amino]ethyl})nitrosoamine	25806	13256-12-7	C4H10N4O2	CN(CCN(C)N=O)N=O
3-Methyl-2-[methyl(nitroso)amino]butan-1-ol	13227489	1823507-82-9	C6H14N2O2	CC(C)C(CO)N(C)N=O
Methyl-2-hydroxypentylnitrosamine	3015879	36972-72-2	C6H14N2O2	CCCC(CN(C)N=O)O
(2*R*)-3-Methyl-2-[methyl(nitroso)amino]butan-1-ol	165799688	2824987-11-1	C6H14N2O2	CC(C)C(CO)N(C)N=O
(2*S*)-3-Methyl-2-[methyl(nitroso)amino]butan-1-ol	13227490	89876-67-5	C6H14N2O2	CC(C)C(CO)N(C)N=O
N-Nitrosoindoline	24274	7633-57-0	C8H8N2O	C1CN(C2=CC=CC=C21)N=O
2-Nitrosoisoindoline	12710701	59164-27-1	C8H8N2O	C1C2=CC=CC=C2CN1N=O
N-Nitroso-(2-hydroxypropyl)-(2-hydroxyethyl)amine	135654	75896-33-2	C5H12N2O3	CC(CN(CCO)N=O)O
4,4-Difluoro-1-nitrosopiperidine	86627845	935259-98-6	C5H8F2N2O	C1CN(CCC1(F)F)N=O
N-Nitrosoethylphenylamine	11929	612-64-6	C8H10N2O	CCN(C1=CC=CC=C1)N=O
N-Methyl-*n*-benzylnitrosamine	13643	937-40-6	C8H10N2O	CN(CC1=CC=CC=C1)N=O
N-Methyl-*N*-nitroso-*m*-toluidine	146404	17485-25-5	C8H10N2O	CC1=CC(=CC=C1)N(C)N=O
1-Nitroso-1H,2H,3H-pyrrolo[2,3-*b*]pyridine	165599025	2731007-77-3	C7H7N3O	C1CN(C2=C1C=CC=N2)N=O
Methyl(nitroso)[(pyridin-3-yl)methyl]amine	235918	6267-76-1	C7H9N3O	CN(CC1=CN=CC=C1)N=O
2-Fluoro-*N*-methyl-*N*-nitrosoaniline	154389598	1978-27-4	C7H7FN2O	CN(C1=CC=CC=C1F)N=O
N-Nitroso-*N*-methyl-4-fluoroaniline	13641	937-25-7	C7H7FN2O	CN(C1=CC=C(C=C1)F)N=O
2-Nitroso-octahydro-1H-isoindole	74025069	23083-75-2	C8H14N2O	C1CCC2CN(CC2C1)N=O
1-Cyclopropyl-4-nitrosopiperazine	154297848	2703774-33-6	C7H13N3O	C1CC1N2CCN(CC2)N=O
1-Acetyl-4-nitrosopiperazine	4185054	73742-56-0	C6H11N3O2	CC(=O)N1CCN(CC1)N=O
1-Nitroso-4-propan-2-ylpiperazine	4184949	73742-54-8	C7H15N3O	CC(C)N1CCN(CC1)N=O
N-Nitrosopipecolic Acid	20614	4515-18-8	C6H10N2O3	C1CCN(C(C1)C(=O)O)N=O
Methyl 1-Nitrosopyrrolidine-3-carboxylate	155978108	2624139-41-7	C6H10N2O3	COC(=O)C1CCN(C1)N=O
N-Nitrosobis(2-oxopropyl)amine	43371	60599-38-4	C6H10N2O3	CC(=O)CN(CC(=O)C)N=O
N-Nitrosoisonipecotic Acid	80387	6238-69-3	C6H10N2O3	C1CN(CCC1C(=O)O)N=O
N-Nitrosodibutylamine (NDBA)	13542	924-16-3	C8H18N2O	CCCCN(CCCC)N=O
N-Nitrosodiisobutylamine	13829	997-95-5	C8H18N2O	CC(C)CN(CC(C)C)N=O
N-Nitrosomethylheptylamine	27822	16338-99-1	C8H18N2O	CCCCCCCN(C)N=O
Nitrosodi-*sec*-butylamine	21447	5350-17-4	C8H18N2O	CCC(C)N(C(C)CC)N=O
2-(4-Nitroso-piperazin-1-yl)-ethanol	12554626	48121-20-6	C6H13N3O2	C1CN(CCN1CCO)N=O
N-Nitrosohydroxyproline (NHPRO)	61873	30310-80-6	C5H8N2O4	C1C(CN(C1C(=O)O)N=O)O
(2*S*,3*R*)-3-Hydroxy-1-nitrosopyrrolidine-2-carboxylic Acid	45115927	2443-29-0	C5H8N2O4	C1CN(C(C1O)C(=O)O)N=O
3-[Nitroso(propan-2-yl)amino]propanoic Acid	165904472	2624134-23-0	C6H12N2O3	CC(C)N(CCC(=O)O)N=O

**Table S7.** *N*-Nitroso compound database imported into the *Search Mass Lists* node (continued)				
N-Nitroso Compound Name	PubChem CID	CAS	Molecular Formula	SMILES
N-Ethyl-*N*-(3-carboxypropyl)nitrosamine	41224	54897-63-1	C6H12N2O3	CCN(CCCC(=O)O)N=O
N-Nitrosoallyl-2,3-dihydroxypropylamine	137413	88208-16-6	C6H12N2O3	C=CCN(CC(CO)O)N=O
(2-Methoxyethyl)(2-methylpropyl)nitrosoamine	132327833	2126177-84-0	C7H16N2O2	CC(C)CN(CCOC)N=O
Propyl(4-hydroxybutyl)nitrosamine	40184	51938-12-6	C7H16N2O2	CCCN(CCCCO)N=O
N-Nitrosothiazolidine-4-carboxylic Acid (NTCA)	104890	88381-44-6	C4H6N2O3S	C1C(N(CS1)N=O)C(=O)O
N-Nitrosoiminodiacetic Acid	32784	25081-31-6	C4H6N2O5	C(C(=O)O)N(CC(=O)O)N=O
1-Nitroso-1,2,3,4-tetrahydroquinoline	817575	5825-44-5	C9H10N2O	C1CC2=CC=CC=C2N(C1)N=O
2-Nitroso-1,2,3,4-tetrahydroisoquinoline	12276866	17721-96-9	C9H10N2O	C1CN(CC2=CC=CC=C21)N=O
2-Methyl-1-nitroso-2,3-dihydro-1H-indole	12598926	85440-79-5	C9H10N2O	CC1CC2=CC=CC=C2N1N=O
N-Nitrosobis(2-methoxyethyl)amine	49986	67856-65-9	C6H14N2O3	COCCN(CCOC)N=O
N-Nitrosobis(2-hydroxypropyl)amine	40828	53609-64-6	C6H14N2O3	CC(CN(CC(C)O)N=O)O
4-Nitroso-1lambda6-thiomorpholine-1,1-dione	155973465	2245709-19-5	C4H8N2O3S	C1CS(=O)(=O)CCN1N=O
4-Nitroso-3,4-dihydro-2H-benzo[*b*][1,4]oxazine	21859611	63169-10-8	C8H8N2O2	C1COC2=CC=CC=C2N1N=O
N-Nitroso-2,3-dihydroxypropylethanolamine	128977	89911-78-4	C5H12N2O4	C(CO)N(CC(CO)O)N=O
N-Nitrosoethylbenzylamine	30228	20689-96-7	C9H12N2O	CCN(CC1=CC=CC=C1)N=O
N-Nitroso-*N*-methyl-2-phenylethylamine	83271	13256-11-6	C9H12N2O	CN(CCC1=CC=CC=C1)N=O
N-Nitroso-*N*-(propan-2-yl)aniline	12249984	24642-83-9	C9H12N2O	CC(C)N(C1=CC=CC=C1)N=O
N-Methyl-*N*,4-dinitrosoaniline	60991	99-80-9	C7H7N3O2	CN(C1=CC=C(C=C1)N=O)N=O
N-Nitrosobetahistine	165718968	32635-81-7	C8H11N3O	CN(CCC1=CC=CC=N1)N=O
4-Methoxy-*N*-methyl-*N*-nitrosobenzenamine	13657	940-11-4	C8H10N2O2	CN(C1=CC=C(C=C1)OC)N=O
5-Nitroso-6,7-dihydro-4H-thieno[3,2-c]pyridine	155820559	2460755-43-3	C7H8N2OS	C1CN(CC2=C1SC=C2)N=O
N-Nitrosobis(2-chloroethyl)amine	45205	67856-68-2	C4H8Cl2N2O	C(CCl)N(CCCl)N=O
2-Chloro-*N*-methyl-*N*-nitrosoaniline	131854034	4461-96-5	C7H7ClN2O	CN(C1=CC=CC=C1Cl)N=O
N-Nitrosoguvacoline	62103	55557-02-3	C7H10N2O3	COC(=O)C1=CCCN(C1)N=O
N-Nitroso-4-*tert*-butylpiperidine	92327	46061-25-0	C9H18N2O	CC(C)(C)C1CCN(CC1)N=O
1-(2-Methylpropyl)-4-nitrosopiperazine	156620437	743372-50-1	C8H17N3O	CC(C)CN1CCN(CC1)N=O
Methyl (2*S*)-2-Methyl-1-nitrosopyrrolidine-2-carboxylate	NA	1818329-35-9	C7H12N2O3	CC1(C(OC)=O)CCCN1N=O
Methyl 1-Nitrosopiperidine-4-carboxylate	12666814	13458-55-4	C7H12N2O3	COC(=O)C1CCN(CC1)N=O
Methyl 1-Nitrosopiperidine-3-carboxylate	141734752	2680535-88-8	C7H12N2O3	COC(=O)C1CCCN(C1)N=O
N-Nitrosomethyloctylamine	36828	34423-54-6	C9H20N2O	CCCCCCCCN(C)N=O
2-(4-Nitrosopiperazin-1-yl)acetic Acid	165905641	2624119-07-7	C6H11N3O3	C1CN(CCN1CC(=O)O)N=O
1-(2-Methoxyethyl)-4-nitrosopiperazine	165946582	2703780-65-6	C7H15N3O2	COCCN1CCN(CC1)N=O
4-[Nitroso(propan-2-yl)amino]butanoic Acid	165905919	2624122-60-5	C7H14N2O3	CC(C)N(CCCC(=O)O)N=O
3-[(2-Methylpropyl)(nitroso)amino]propanoic Acid	165905925	2624139-69-9	C7H14N2O3	CC(C)CN(CCC(=O)O)N=O
N-Propyl-*N*-(3-carboxypropyl)nitrosamine	41843	56316-37-1	C7H14N2O3	CCCN(CCCC(=O)O)N=O
N-Nitroso-2-methylthiazolidine 4-carboxylic Acid (NMTCA)	115101	103659-08-1	C5H8N2O3S	CC1N(C(CS1)C(=O)O)N=O
1-Nitroso-2-phenylpyrrolidine	117066584	3237-85-2	C10H12N2O	C1CC(N(C1)N=O)C2=CC=CC=C2
N-Benzyl-*N*-nitrosocyclopropanamine	10702425	257946-84-2	C10H12N2O	C1CC1N(CC2=CC=CC=C2)N=O
N-Nitrosonornicotine (NNN)	27919	16543-55-8	C9H11N3O	C1CC(N(C1)N=O)C2=CN=CC=C2
N-Methyl-*N*-nitroso-1H-purin-6-amine	30861	21928-82-5	C6H6N6O	CN(C1=NC=NC2=C1NC=N2)N=O
2-Nitroso-1,2,3,4-tetrahydroisoquinolin-6-ol	165997333	2763758-97-8	C9H10N2O2	C1CN(CC2=C1C=C(C=C2)O)N=O

**Table S7.** *N*-Nitroso compound database imported into the *Search Mass Lists* node (continued)				
N-Nitroso Compound Name	PubChem CID	CAS	Molecular Formula	SMILES
N-Nitroso-*tert*-butylphenylamine	3336608	24642-84-0	C10H14N2O	CC(C)(C)N(C1=CC=CC=C1)N=O
N-Butyl-*N*-nitrosobenzenamine	146990	25413-69-8	C10H14N2O	CCCCN(C1=CC=CC=C1)N=O
N-Nitroso-*N*-phenylglycine	3767411	6415-68-5	C8H8N2O3	C1=CC=C(C=C1)N(CC(=O)O)N=O
N-Nitroso-*N*-(4-methoxybenzyl)methylamine	55202	84174-20-9	C9H12N2O2	CN(CC1=CC=C(C=C1)OC)N=O
N-Methyl-4-nitro-*N*-nitrosobenzenamine	13671	943-41-9	C7H7N3O3	CN(C1=CC=C(C=C1)[N+](=O)[O-])N=O
1-Cyclopropanecarbonyl-4-nitrosopiperazine	165691461	2742659-60-3	C8H13N3O2	C1CC1C(=O)N2CCN(CC2)N=O
1-Cyclopentyl-4-nitrosopiperazine	15895852	61379-66-6	C9H17N3O	C1CCC(C1)N2CCN(CC2)N=O
Ethyl 1-Nitrosopiperidine-4-carboxylate	122432208	160114-74-9	C8H14N2O3	CCOC(=O)C1CCN(CC1)N=O
N-Nitrosodiamylamine	252237	13256-06-9	C10H22N2O	CCCCCN(CCCCC)N=O
N-Methyl-*N*-nitrosoquinolin-8-amine	119086951	15143-01-8	C10H9N3O	CN(C1=CC=CC2=C1N=CC=C2)N=O
N-Nitroso-*N'*-carbethoxypiperazine	25808	13256-15-0	C7H13N3O3	CCOC(=O)N1CCN(CC1)N=O
4-[*tert*-Butyl(nitroso)amino]butanoic Acid	43874	62018-93-3	C8H16N2O3	CC(C)(C)N(CCCC(=O)O)N=O
N-Nitrosoanatabine (NAT)	528366	887407-16-1	C10H11N3O	C1C=CCN(C1C2=CN=CC=C2)N=O
1-Nitroso-4-phenylpiperidine	81163	6652-04-6	C11H14N2O	C1CN(CCC1C2=CC=CC=C2)N=O
N-Nitrosoanabasine (NAB)	14335	1133-64-8	C10H13N3O	C1CCN(C(C1)C2=CN=CC=C2)N=O
1-Nitroso-4-phenylpiperazine	3014828	14340-33-1	C10H13N3O	C1CN(CCN1C2=CC=CC=C2)N=O
[(3*S*)-2-Nitroso-1,2,3,4-tetrahydroisoquinolin-3-yl]methanol	155943658	2613299-79-7	C10H12N2O2	C1C(N(CC2=CC=CC=C21)N=O)CO
[(3*R*)-2-Nitroso-1,2,3,4-tetrahydroisoquinolin-3-yl]methanol	155978169	2624108-82-1	C10H12N2O2	C1C(N(CC2=CC=CC=C21)N=O)CO
1-Nitroso-4-(pyridin-2-yl)piperazine	72735916	872825-79-1	C9H12N4O	C1CN(CCN1C2=CC=CC=N2)N=O
1-(Methylsulfonyl)-4-nitrosopiperazine	54775704	1190890-92-6	C5H11N3O3S	CS(=O)(=O)N1CCN(CC1)N=O
N-{[Methyl(nitroso)amino]methyl}benzamide	108799	59665-02-0	C9H11N3O2	CN(CNC(=O)C1=CC=CC=C1)N=O
2-(4-Nitrosopiperazin-1-yl)pyrimidine	155289978	872826-80-7	C8H11N5O	C1CN(CCN1C2=NC=CC=N2)N=O
N-Nitrosoephedrine	28638	17608-59-2	C10H14N2O2	CC(C(C1=CC=CC=C1)O)N(C)N=O
3-[Benzyl(nitroso)amino]propan-1-ol	130124782	39885-22-8	C10H14N2O2	C1=CC=C(C=C1)CN(CCCO)N=O
N-Nitrosophenylephrine	165729371	78658-64-7	C9H12N2O3	CN(CC(C1=CC(=CC=C1)O)O)N=O
Mononitrosocaffeidine	126865	145438-96-6	C7H11N5O2	CNC(=O)C1=C(N=CN1C)N(C)N=O
1'-Nitroso-1,4'-bipiperidine	155970807	2639422-25-4	C10H19N3O	C1CCN(CC1)C2CCN(CC2)N=O
N-Nitrosoglyphosate	41910	56516-72-4	C3H7N2O6P	C(C(=O)O)N(CP(=O)(O)O)N=O
N-Nitrosodiphenylamine (NDPhA)	6838	86-30-6	C12H10N2O	C1=CC=C(C=C1)N(C2=CC=CC=C2)N=O
3-[Methyl(nitroso)amino]-1-(thiophen-2-yl)propan-1-ol	154577082	2418708-78-6	C8H12N2O2S	CN(CCC(C1=CC=CS1)O)N=O
N-Nitrosorasagiline	155819601	2470278-90-9	C12H12N2O	C#CCN(C1CCC2=CC=CC=C12)N=O
Methyl[(naphthalen-1-yl)methyl]nitrosoamine	10821900	296760-88-8	C12H12N2O	CN(CC1=CC=CC2=CC=CC=C21)N=O
tert-Butyl 1-Nitrosopyrrolidine-3-carboxylate	167734014	2919945-81-4	C9H16N2O3	CC(C)(C)OC(=O)C1CCN(C1)N=O
2,3,5,6-Tetramethyl-1,4-dinitrosopiperazine	148371	63441-59-8	C8H16N4O2	CC1C(N(C(C(N1N=O)C)C)N=O)C
N-Methyl-*N*-nitroso-4-(trifluoromethyl)aniline	23278140	91385-14-7	C8H7F3N2O	CN(C1=CC=C(C=C1)C(F)(F)F)N=O
1-Benzyl-4-nitrosopiperazine	218466	40675-45-4	C11H15N3O	C1CN(CCN1CC2=CC=CC=C2)N=O
1-(2-Methylphenyl)-4-nitrosopiperazine	165739385	2751615-89-9	C11H15N3O	CC1=CC=CC=C1N2CCN(CC2)N=O
1-(4-Methylphenyl)-4-nitrosopiperazine	165946586	2703780-36-1	C11H15N3O	CC1=CC=C(C=C1)N2CCN(CC2)N=O
1-(3-Methylphenyl)-4-nitrosopiperazine	165759459	2758004-65-6	C11H15N3O	CC1=CC(=CC=C1)N2CCN(CC2)N=O
1-Nitroso-4-phenylpiperidin-4-ol	119087581	16533-05-4	C11H14N2O2	C1CN(CCC1(C2=CC=CC=C2)O)N=O
4-(Methylnitrosamino)-1-(3-pyridyl)-1-butanone (NNK)	47289	64091-91-4	C10H13N3O2	CN(CCCC(=O)C1=CN=CC=C1)N=O

**Table S7.** *N*-Nitroso compound database imported into the *Search Mass Lists* node (continued)				
N-Nitroso Compound Name	PubChem CID	CAS	Molecular Formula	SMILES
4-(4-Fluorophenyl)-1-nitrosopiperidine	85949641	6716-99-0	C11H13FN2O	C1CN(CCC1C2=CC=C(C=C2)F)N=O
[1-(2-Methoxyphenyl)propan-2-yl](methyl)nitrosoamine	154382820	2768326-80-1	C11H16N2O2	CC(CC1=CC=CC=C1OC)N(C)N=O
1-(2-Fluorophenyl)-4-nitrosopiperazine	165599027	2731009-71-3	C10H12FN3O	C1CN(CCN1C2=CC=CC=C2F)N=O
4-(Methylnitrosamino)-1-(3-pyridyl)-1-butanol (NNAL)	104856	76014-81-8	C10H15N3O2	CN(CCCC(C1=CN=CC=C1)O)N=O
N-Nitrosobis(2,2,2-trifluoroethyl)amine	12267	625-89-8	C4H4F6N2O	C(C(F)(F)F)N(CC(F)(F)F)N=O
N-Nitrosodicyclohexylamine	13697	947-92-2	C12H22N2O	C1CCC(CC1)N(C2CCCCC2)N=O
N-Nitrosophenylbenzylamine	11935	612-98-6	C13H12N2O	C1=CC=C(C=C1)CN(C2=CC=CC=C2)N=O
1-Methyl-4-(1-nitrosopiperidin-4-yl)piperazine	126705144	2089333-01-5	C10H20N4O	CN1CCN(CC1)C2CCN(CC2)N=O
1-Nitroso-4-(oxolane-2-carbonyl)piperazine	155819615	2470438-57-2	C9H15N3O3	C1CC(OC1)C(=O)N2CCN(CC2)N=O
5-Bromo-*N*-methyl-*N*-nitrosopyrimidin-2-amine	165589847	2731008-49-2	C5H5BrN4O	CN(C1=NC=C(C=N1)Br)N=O
1-Benzoyl-4-nitrosopiperazine	4185056	73742-55-9	C11H13N3O2	C1CN(CCN1C(=O)C2=CC=CC=C2)N=O
7-Nitroso-3-(trifluoromethyl)-5,6,7,8-tetrahydro-[1,2,4]triazolo[4,3-*a*]pyrazine	165984324	1481965-39-2	C6H6F3N5O	C1CN2C(=NN=C2C(F)(F)F)CN1N=O
1-(2-Methoxyphenyl)-4-nitrosopiperazine	154814146	2219339-64-5	C11H15N3O2	COC1=CC=CC=C1N2CCN(CC2)N=O
1-(4-Methoxyphenyl)-4-nitrosopiperazine	12952715	75051-59-1	C11H15N3O2	COC1=CC=C(C=C1)N2CCN(CC2)N=O
1-(3-Methoxyphenyl)-4-nitrosopiperazine	165989139	2763779-41-3	C11H15N3O2	COC1=CC=CC(=C1)N2CCN(CC2)N=O
2-((4-Acetylphenyl)(nitroso)amino)acetic Acid	13123955	84968-84-3	C10H10N2O4	CC(=O)C1=CC=C(C=C1)N(CC(=O)O)N=O
N-Nitrosoiminostilbene	162317	38652-29-8	C14H10N2O	C1=CC=C2C(=C1)C=CC3=CC=CC=C3N2N=O
(1*R*)-8-Chloro-1-methyl-3-nitroso-2,3,4,5-tetrahydro-1H-3-benzazepine	165796483	2518136-84-8	C11H13ClN2O	CC1CN(CCC2=C1C=C(C=C2)Cl)N=O
N-Nitrosoiminodibenzyl	81960	7458-08-4	C14H12N2O	C1CC2=CC=CC=C2N(C3=CC=CC=C31)N=O
1-(3-Chlorophenyl)-4-nitrosopiperazine	155819595	2219339-13-4	C10H12ClN3O	C1CN(CCN1C2=CC(=CC=C2)Cl)N=O
1-(2-Chlorophenyl)-4-nitrosopiperazine	165739386	2221987-86-4	C10H12ClN3O	C1CN(CCN1C2=CC=CC=C2Cl)N=O
N-Nitrosodibenzylamine	21421	5336-53-8	C14H14N2O	C1=CC=C(C=C1)CN(CC2=CC=CC=C2)N=O
2-(4-Nitrosopiperazin-1-yl)-1-(pyrrolidin-1-yl)ethan-1-one	165981586	2870652-88-1	C10H18N4O2	C1CCN(C1)C(=O)CN2CCN(CC2)N=O
N-Nitrosopyrimethanil	14327551	116389-21-0	C12H12N4O	CC1=CC(=NC(=N1)N(C2=CC=CC=C2)N=O)C
5-Bromo-1-nitroso-1H,2H,3H-pyrrolo[2,3-*b*]pyridine	165588258	2731007-22-8	C7H6BrN3O	C1CN(C2=C1C=C(C=N2)Br)N=O
5,7-Dichloro-2-nitroso-3,4-dihydro-1H-isoquinoline	155820612	2460755-61-5	C9H8Cl2N2O	C1CN(CC2=C1C(=CC(=C2)Cl)Cl)N=O
N-Nitrososimazine	5216	6494-81-1	C7H11ClN6O	CCNC1=NC(=NC(=N1)Cl)N(CC)N=O
2-(1-Nitrosopiperidin-4-yl)-1H-1,3-benzodiazole	165690044	2731008-04-9	C12H14N4O	C1CN(CCC1C2=NC3=CC=CC=C3N2)N=O
5-Bromo-2-nitroso-1,2,3,4-tetrahydroisoquinoline	165978890	2758004-13-4	C9H9BrN2O	C1CN(CC2=C1C(=CC=C2)Br)N=O
4-(4-Chlorophenyl)-1-nitrosopiperidin-4-ol	119089663	3192-36-7	C11H13ClN2O2	C1CN(CCC1(C2=CC=C(C=C2)Cl)O)N=O
N-Nitrosoatrazine	108077	56525-09-8	C8H13ClN6O	CCN(C1=NC(=NC(=N1)NC(C)C)Cl)N=O
5,7-Dichloro-2-nitroso-1,2,3,4-tetrahydroisoquinolin-6-ol	165747057	2803861-31-4	C9H8Cl2N2O2	C1CN(CC2=CC(=C(C(=C21)Cl)O)Cl)N=O
1-(1-Benzothiophen-4-yl)-4-nitrosopiperazine	155970798	2639426-95-0	C12H13N3OS	C1CN(CCN1C2=C3C=CSC3=CC=C2)N=O
(2*R*,6*S*)-1-Benzoyl-2,6-dimethyl-4-nitrosopiperazine	121231125	2792161-47-6	C13H17N3O2	CC1CN(CC(N1C(=O)C2=CC=CC=C2)C)N=O
2-(1-Nitrosopiperidin-4-yl)-2-phenylacetic Acid	165905790	2932440-73-6	C13H16N2O3	C1CN(CCC1C(C2=CC=CC=C2)C(=O)O)N=O
6-Fluoro-3-(1-nitrosopiperidin-4-yl)-1,2-benzoxazole	146155478	2416230-38-9	C12H12FN3O2	C1CN(CCC1C2=NOC3=C2C=CC(=C3)F)N=O
1-(2,3-Dichlorophenyl)-4-nitrosopiperazine	165605069	2989508-28-1	C10H11Cl2N3O	C1CN(CCN1C2=C(C(=CC=C2)Cl)Cl)N=O
1-Nitroso-4-[4-(trifluoromethyl)phenyl]piperazine	165845529	2758000-23-4	C11H12F3N3O	C1CN(CCN1C2=CC=C(C=C2)C(F)(F)F)N=O
N-Nitrosofenfluramine	101196224	19023-40-6	C12H15F3N2O	CCN(C(C)CC1=CC(=CC=C1)C(F)(F)F)N=O
N-Nitrosomethylphenidate	41474	55557-03-4	C14H18N2O3	COC(=O)C(C1CCCCN1N=O)C2=CC=CC=C2
Dinitrosoethambutol	121489243	2792161-95-4	C10H22N4O4	CCC(CO)N(CCN(C(CC)CO)N=O)N=O

**Table S7.** *N*-Nitroso compound database imported into the *Search Mass Lists* node (continued)				
N-Nitroso Compound Name	PubChem CID	CAS	Molecular Formula	SMILES
2-({2-[(1-Hydroxybutan-2-yl)(nitroso)amino]ethyl}(nitroso)amino)butan-1-ol	47559	65229-18-7	C10H22N4O4	C(CCO)CN(CCN(CCCCO)N=O)N=O
2-{[2-(4-Nitrosopiperazin-1-yl)pyrimidin-5-yl]oxy}acetic Acid	165908124	2680542-96-3	C10H13N5O4	C1CN(CCN1C2=NC=C(C=N2)OCC(=O)O)N=O
4-(4-Bromophenyl)-1-nitrosopiperidine	149808353	2455384-06-0	C11H13BrN2O	C1CN(CCC1C2=CC=C(C=C2)Br)N=O
2-[*tert*-Butyl(nitroso)amino]-1-(3-chlorophenyl)propan-1-one	165995445	2763780-10-3	C13H17ClN2O2	CC(C(=O)C1=CC(=CC=C1)Cl)N(C(C)(C)C)N=O
N-Nitrosoalbuterol	167719308	2919946-71-5	C13H20N2O4	CC(C)(C)N(CC(C1=CC(=C(C=C1)O)CO)O)N=O
1-(4-Bromophenyl)-4-nitrosopiperazine	155973294	2648941-13-1	C10H12BrN3O	C1CN(CCN1C2=CC=C(C=C2)Br)N=O
N-Nitroso-di-*n*-octylamine	22803	6335-97-3	C16H34N2O	CCCCCCCCN(CCCCCCCC)N=O
1-Diphenylmethyl-4-nitrosopiperazine	164870	1698-25-5	C17H19N3O	C1CN(CCN1C(C2=CC=CC=C2)C3=CC=CC=C3)N=O
N-Nitrosopropranolol	128614	84418-35-9	C16H20N2O3	CC(C)N(CC(COC1=CC=CC2=CC=CC=C21)O)N=O
N-Nitrosonortriptyline	165459181	55855-42-0	C19H20N2O	CN(CCC=C1C2=CC=CC=C2CCC3=CC=CC=C31)N=O
N-Nitrosodesmethylvenlafaxine	165941412	2680662-11-5	C16H24N2O3	CN(CC(C1=CC=C(C=C1)OC)C2(CCCCC2)O)N=O
N-Nitrosoatenolol	154448	134720-04-0	C14H21N3O4	CC(C)N(CC(COC1=CC=C(C=C1)CC(=O)N)O)N=O
N-Nitrosodesipramine	2995	57164-17-7	C18H21N3O	CN(CCCN1C2=CC=CC=C2CCC3=CC=CC=C31)N=O
N-Nitrosometoprolol	154449	134720-05-1	C15H24N2O4	CC(C)N(CC(COC1=CC=C(C=C1)CCOC)O)N=O
N-Nitrososotalol	154451	134720-07-3	C12H19N3O4S	CC(C)N(CC(C1=CC=C(C=C1)NS(=O)(=O)C)O)N=O
Nintedanib 4-nitrophenyl 2-(4-Nitroso)	155820971	2490403-92-2	C13H17N5O4	CN(C1=CC=C(C=C1)[N+](=O)[O-])C(=O)CN2CCN(CC2)N=O
N-Nitrosopendimethalin	50260	68897-50-7	C13H18N4O5	CCC(CC)N(C1=C(C=C(C(=C1[N+](=O)[O-])C)C)[N+](=O)[O-])N=O
N-Nitrosodesisopropyltolterodine	165941884	2680528-95-2	C19H24N2O2	CC1=CC(=C(C=C1)O)C(CCN(C(C)C)N=O)C2=CC=CC=C2
1-[(4-Chlorophenyl)-phenylmethyl]-4-nitrosopiperazine	12434823	2005-04-1	C17H18ClN3O	C1CN(CCN1C(C2=CC=CC=C2)C3=CC=C(C=C3)Cl)N=O
N-Nitrosodiclofenac	9797358	66505-80-4	C14H10Cl2N2O3	C1=CC=C(C(=C1)CC(=O)O)N(C2=C(C=CC=C2Cl)Cl)N=O
N-Nitrosoduloxetine	165934748	2680527-91-5	C18H18N2O2S	CN(CCC(C1=CC=CS1)OC2=CC=CC3=CC=CC=C32)N=O
N-Nitrosovildagliptin	165706756	NA	C17H24N4O3	C1CC(N(C1)C(=O)CN(C23CC4CC(C2)CC(C4)(C3)O)N=O)C#N
N-Nitrosofluoxetine	9840784	150494-06-7	C17H17F3N2O2	CN(CCC(C1=CC=CC=C1)OC2=CC=C(C=C2)C(F)(F)F)N=O
N-Nitrosonadolol	154450	134720-06-2	C17H26N2O5	CC(C)(C)N(CC(COC1=CC=CC2=C1CC(C(C2)O)O)O)N=O
N-Nitrosodesloratadine	71751122	1246819-22-6	C19H18ClN3O	C1CC2=C(C=CC(=C2)Cl)C(=C3CCN(CC3)N=O)C4=C1C=CC=N4
N-Nitrosonorfloxacin	13320456	74011-53-3	C16H17FN4O4	CCN1C=C(C(=O)C2=CC(=C(C=C21)N3CCN(CC3)N=O)F)C(=O)O
N-Nitrosoparoxetine	165599028	2361294-43-9	C19H19FN2O4	C1CN(CC(C1C2=CC=C(C=C2)F)COC3=CC4=C(C=C3)OCO4)N=O
N-Nitrosociprofloxacin	87856327	864443-44-7	C17H17FN4O4	C1CC1N2C=C(C(=O)C3=CC(=C(C=C32)N4CCN(CC4)N=O)F)C(=O)O
N-Nitrosovonoprazan	168447669	NA	C17H15FN4O3S	CN(CC1=CN(C(=C1)C2=CC=CC=C2F)S(=O)(=O)C3=CN=CC=C3)N=O
2-[Ethyl(nitroso)amino]ethyl 3-(Naphthalen-1-yl)-2-[(oxolan-2-yl)methyl]propanoate	165933770	2680540-75-2	C22H28N2O4	CCN(CCOC(=O)C(CC1CCCO1)CC2=CC=CC3=CC=CC=C32)N=O
N-Nitrosobumetanide	155970256	2490432-02-3	C17H19N3O6S	CCCCN(C1=C(C(=CC(=C1)C(=O)O)S(=O)(=O)N)OC2=CC=CC=C2)N=O
4-(4-Fluoro-3-(4-nitrosopiperazine-1-carbonyl)benzyl)phthalazin-1(2H)-one	165711920	2742659-74-9	C20H18FN5O3	C1CN(CCN1C(=O)C2=C(C=CC(=C2)CC3=NNC(=O)C4=CC=CC=C43)F)N=O
N-Nitrosonebivolol	71751137	1391051-68-5	C22H24F2N2O5	C1CC2=C(C=CC(=C2)F)OC1C(CN(CC(C3CCC4=C(O3)C=CC(=C4)F)O)N=O)O
N-Nitrosoflecainide	167735110	2901109-58-6	C17H19F6N3O4	C1CCN(C(C1)CNC(=O)C2=C(C=CC(=C2)OCC(F)(F)F)OCC(F)(F)F)N=O
Di *N*-Nitrosoflecainide	167728410	2901085-00-3	C17H18F6N4O5	C1CCN(C(C1)CN(C(=O)C2=C(C=CC(=C2)OCC(F)(F)F)OCC(F)(F)F)N=O)N=O

6. Calculation of TONO scores for specific *N*-nitrosamines

Table S8. SPE recoveries and conversion efficiencies of specific *N*-nitrosamines and their TONO scores

Compound	Molecular Structure	SPE Recovery	Conversion Efficiency	TONO Score	Category
N-Nitrosodimethylamine	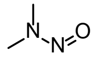	82±6%	100±8%	0.82±0.06	dialkyl (EPA 521)
N-Nitrosomethylethylamine	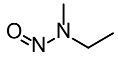	75±7%	99±8%	0.74±0.06	dialkyl (EPA 521)
N-Nitrosodiethylamine	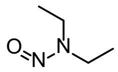	76±4%	96±8%	0.73±0.05	dialkyl (EPA 521)
N-Nitrosodipropylamine	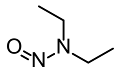	76±5%	95±13%	0.72±0.08	dialkyl (EPA 521)
N-Nitrosodibutylamine	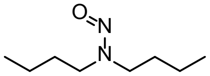	63±5%	98±9%	0.61±0.05	dialkyl (EPA 521)
N-Nitrosopyrrolidine	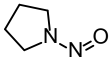	89±7%	97±11%	0.86±0.08	cyclic (EPA 521)
N-Nitrosopiperidine	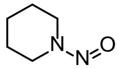	86±9%	93±8%	0.80±0.06	cyclic (EPA 521)
N-Nitrosomorpholine	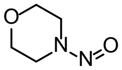	81±10%	94±16%	0.75±0.10	cyclic
N-Nitrosodiphenylamine	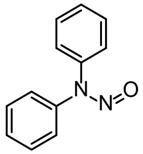	26±1%	83±11%	0.21±0.02	diaryl
N-Nitrosodibenzylamine	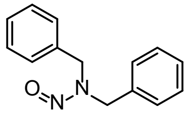	29±6%	81±11%	0.24±0.03	dialkyl
N-Nitrosodiethanolamine	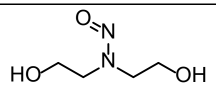	37±5%	78±8%	0.28±0.03	dialkyl
N-Nitrosohydroxyproline	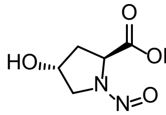	49±4%	68±12%	0.33±0.04	cyclic

**Table S8.** SPE recoveries and conversion efficiencies of specific *N*-nitrosamines and their TONO scores (continued)					
Compound	Molecular Structure	SPE Recovery	Conversion Efficiency	TONO Score	Category
N-Nitrosoanabasine	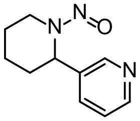	29±4%	90±10%	0.26±0.03	cyclic
N-Nitrosoanatabine	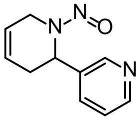	25±3%	90±10%	0.23±0.02	cyclic
N-Nitrosonornicotine	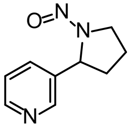	27±2%	91±10%	0.24±0.02	cyclic
4-(Methylnitrosamino)-1-(3-Pyridyl)-1-Butanone	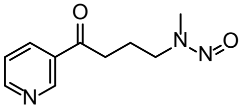	29±2%	92±9%	0.26±0.02	dialkyl
N-Nitrosodicyclohexylamine	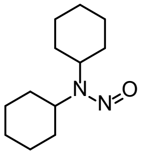	43±7%	91±10%	0.40±0.04	dicycloalkyl
N-Nitrosoatrazine	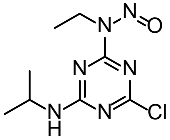	29±3%	72±11%	0.21±0.03	alkylheteroaryl
1-Nitroso-4-Phenylpiperazine	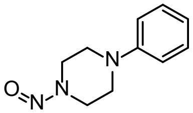	54±5%	77±11%	0.41±0.04	heterocyclic
N-Nitroso-*tert*-Butylphenylamine	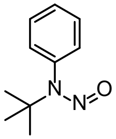	79±2%	79±11%	0.62±0.06	alkylaryl
N-Nitrosofenfluramine	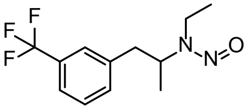	78±7%	89±9%	0.69±0.06	dialkyl
N-Nitrosomethylamylamine	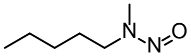	81±5%	93±10%	0.76±0.06	dialkyl

**Table S8.** SPE recoveries and conversion efficiencies of specific *N*-nitrosamines and their TONO scores (continued)					
Compound	Molecular Structure	SPE Recovery	Conversion Efficiency	TONO Score	Category
N-Nitrosodiisopropylamine	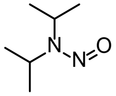	73±7%	76±11%	0.55±0.06	dialkyl
N-Nitrosodiamylamine	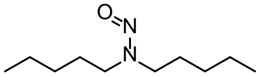	43±8%	98±10%	0.42±0.05	dialkyl
N-Nitrosomethylbutylamine	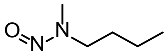	88±6%	92±10%	0.81±0.07	dialkyl
N-Nitrosoethylbenzylamine	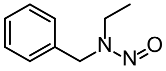	79±6%	83±10%	0.65±0.06	dialkyl
N-Nitrosomethylisopropylamine	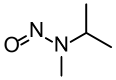	77±5%	90±10%	0.69±0.06	dialkyl
N-Nitroso-*tert*-Butylmethylamine	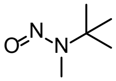	90±5%	83±11%	0.74±0.07	dialkyl
N-Nitrosoethylpropylamine	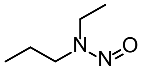	85±8%	92±10%	0.78±0.07	dialkyl
N-Nitrosomethylisobutylamine	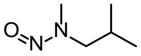	80±8%	86±12%	0.68±0.07	dialkyl
N-Nitroso-2-Pyrrolidinmethanol	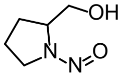	45±5%	93±10%	0.42±0.04	cyclic
N-Nitrosoethylphenylamine	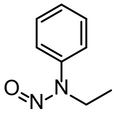	37±4%	86±10%	0.32±0.03	alkylaryl
N-Nitrosomethylcyclohexylamine	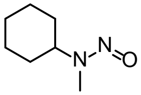	90±7%	88±10%	0.79±0.07	alkylcycloalkyl
N-Nitroso-*tert*-Butylethylamine	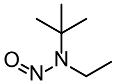	78±5%	81±9%	0.63±0.05	dialkyl
1-Nitroso-4-Methylpiperidine	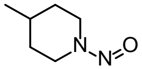	93±3%	97±7%	0.90±0.05	cyclic
N-Nitrosofluoxetine	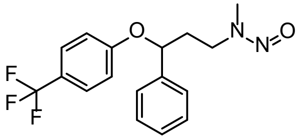	36±6%	94±9%	0.34±0.04	dialkyl

**Table S8.** SPE recoveries and conversion efficiencies of specific *N*-nitrosamines and their TONO scores (continued)					
Compound	Molecular Structure	SPE Recovery	Conversion Efficiency	TONO Score	Category
N-Nitrosodesloratadine	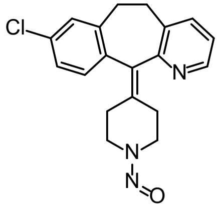	48±12%	96±8%	0.46±0.06	cyclic
N-Nitrosometoprolol	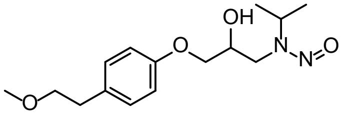	49±7%	89±9%	0.44±0.04	dialkyl
N-Nitrosoephedrine	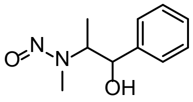	59±11%	91±9%	0.54±0.06	dialkyl
N-Nitrosodesipramine	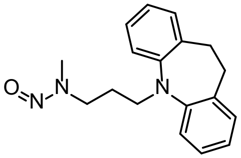	43±5%	90±11%	0.39±0.04	dialkyl
N-Nitrosoiminostilbene	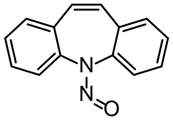	41±5%	84±10%	0.34±0.04	cyclic diaryl

7. Concentration profiles of specific *N*-nitrosamines and TONO across sampling events

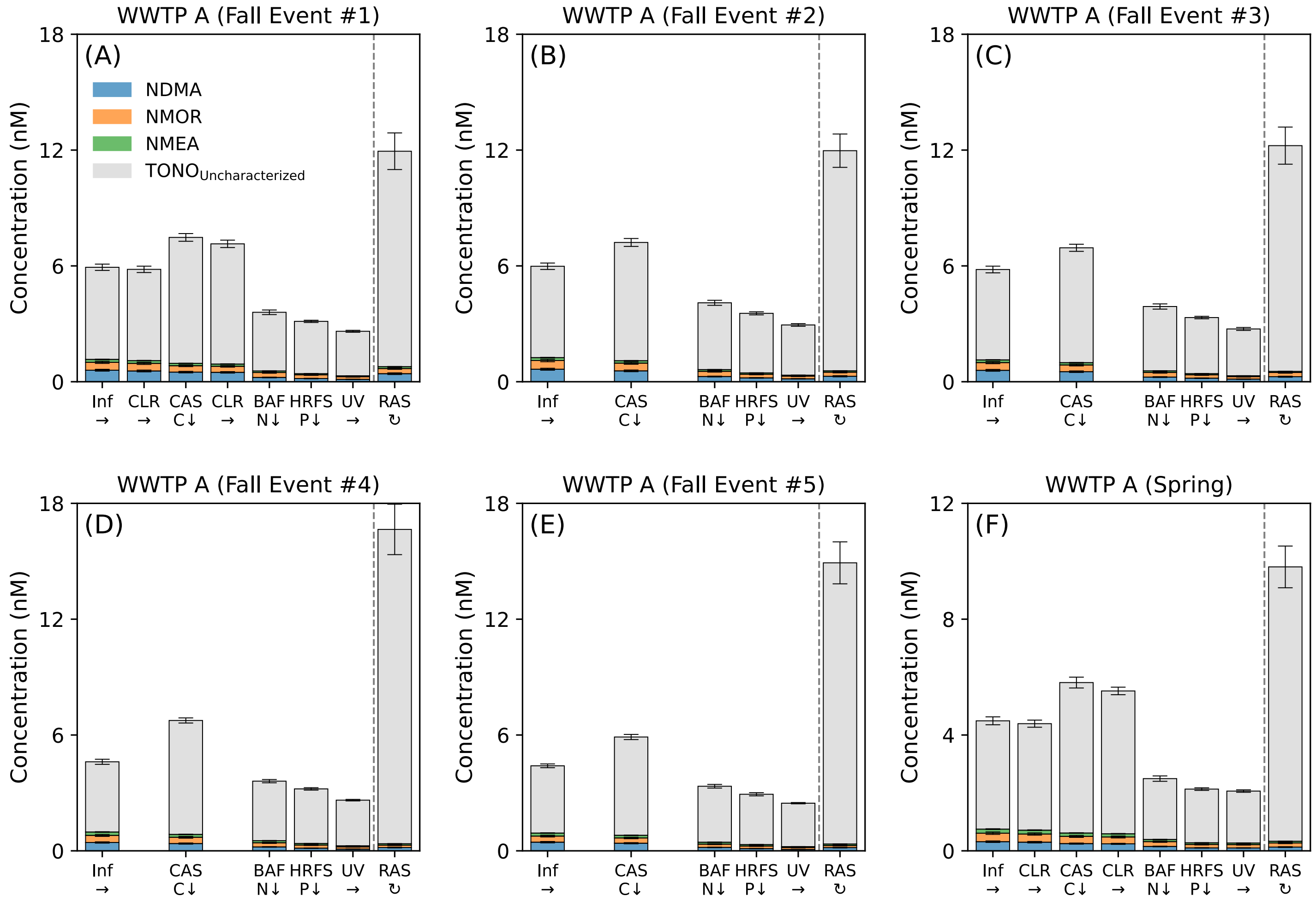

Figure S5. Concentration profiles of specific *N*-nitrosamines and TONO (i.e., the sum of specific and uncharacterized *N*-nitrosamines) for different sampling events at WWTP A (i.e., Fall: **(A)-(E)** and Spring: **(F)**). Inf = plant influent; CLR = primary or secondary clarifier effluent; CAS = conventional activated sludge aeration tank effluent; POAS = pure oxygen activated sludge aeration tank effluent; EAAS = extended aeration activated sludge aeration tank effluent; AER = post-aeration tank effluent; BAF = biological aerated filter effluent; BSF = biological sand filter effluent; TF = trickling filter effluent; RBC = rotating biological contactor effluent; HRFS = high-rate flocculated settling tank effluent; CMDF = cloth media disc filter effluent; LAG = lagoon effluent; UV = post-UV effluent; Cl_2 = post-chlorination effluent; and RAS = returned activated sludge supernatant. "→" indicates the flow direction. "↓" indicates BOD removal, nitrification, or phosphorus precipitation. "↻" indicates sludge recirculation. Error bars represent the standard deviations from duplicate measurements of specific *N*-nitrosamines and TONO. Note the differences in the *y*-axis scales. Note that for each sample, TONO measured by HI_3-CL were partitioned into specific and uncharacterized components according to the equation:

$$\mathrm{TONO} = \mathrm{TONO}_{\mathrm{Specific}} + \mathrm{TONO}_{\mathrm{Uncharacterized}} = \sum_{i=1}^{3}\left(C_{N\text{-Nitrosamine}_i} \times \mathrm{TONO\ Score}_{N\text{-Nitrosamine}_i}\right) + \mathrm{TONO}_{\mathrm{Uncharacterized}}$$

$$= \sum_{i=1}^{3}\left(C_{N\text{-Nitrosamine}_i} \times \mathrm{SPE\ Recovery}_{N\text{-Nitrosamine}_i} \times \mathrm{Conversion\ Efficiency}_{N\text{-Nitrosamine}_i}\right)$$

$$+ \mathrm{TONO}_{\mathrm{Uncharacterized}}$$

where the summed contribution of target *N*-nitrosamines (i.e., NDMA, NMOR, and NMEA) to TONO was calculated by correcting their molar concentrations measured by LC-HRMS with their respective TONO scores.

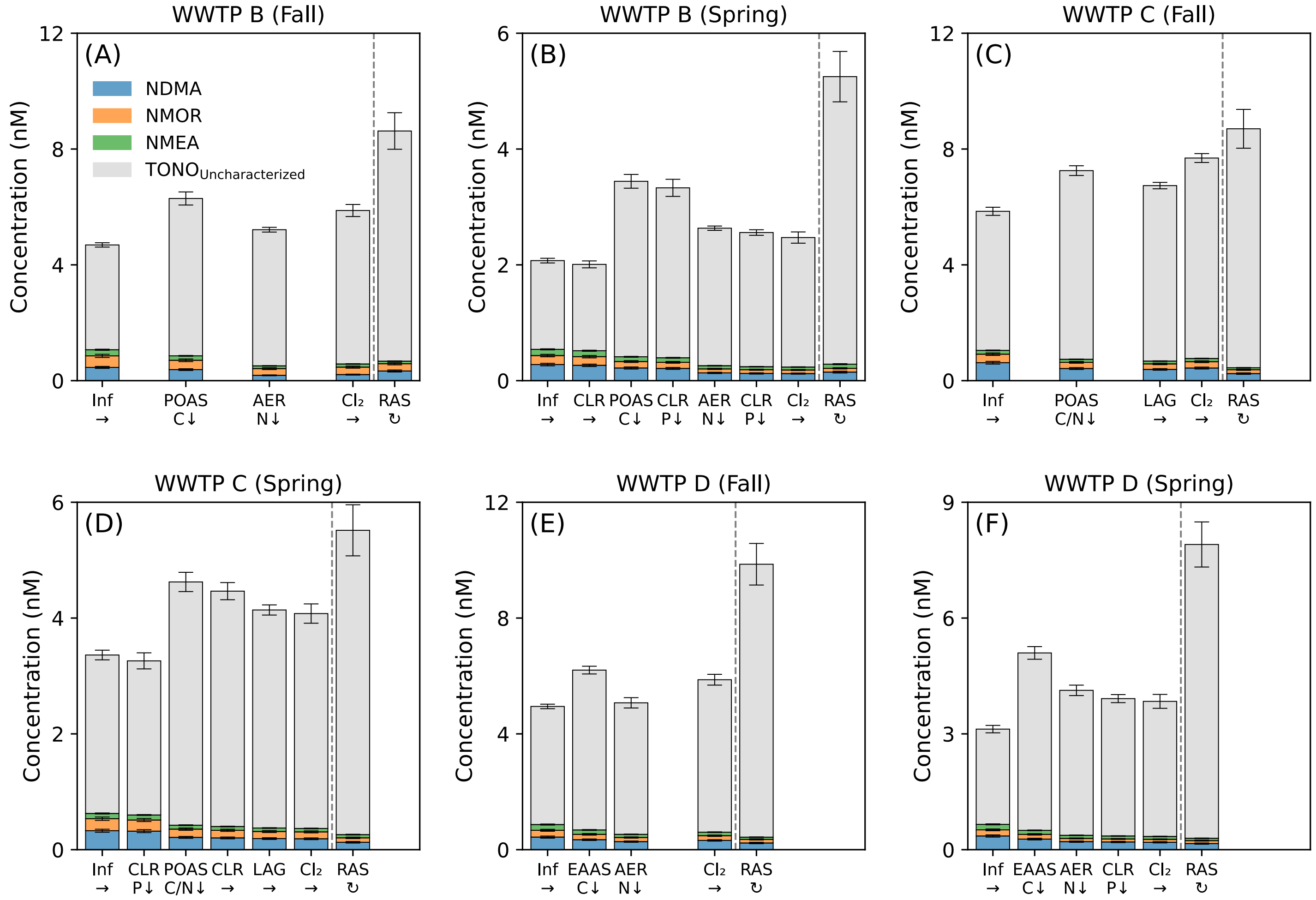

Figure S6. Concentration profiles of specific *N*-nitrosamines and TONO (i.e., the sum of specific and uncharacterized *N*-nitrosamines) for different sampling events at WWTPs B (i.e., Fall: **(A)** and Spring: **(B)**), C (i.e., Fall: **(C)** and Spring: **(D)**), and D (i.e., Fall: **(E)** and Spring: **(F)**). Inf = plant influent; CLR = primary or secondary clarifier effluent; CAS = conventional activated sludge aeration tank effluent; POAS = pure oxygen activated sludge aeration tank effluent; EAAS = extended aeration activated sludge aeration tank effluent; AER = post-aeration tank effluent; BAF = biological aerated filter effluent; BSF = biological sand filter effluent; TF = trickling filter effluent; RBC = rotating biological contactor effluent; HRFS = high-rate flocculated settling tank effluent; CMDF = cloth media disc filter effluent; LAG = lagoon effluent; UV = post-UV effluent; Cl_2 = post-chlorination effluent; and RAS = returned activated sludge supernatant. "→" indicates the flow direction. "↓" indicates BOD removal, nitrification, or phosphorus precipitation. "↻" indicates sludge recirculation. Error bars represent the standard deviations from duplicate measurements of specific *N*-nitrosamines and TONO. Note the differences in the *y*-axis scales. Note that for each sample, TONO measured by HI_3-CL were partitioned into specific and uncharacterized components according to the equation:

$$\mathrm{TONO} = \mathrm{TONO}_{\mathrm{Specific}} + \mathrm{TONO}_{\mathrm{Uncharacterized}} = \sum_{i=1}^{3}\left(C_{N\text{-Nitrosamine}_i} \times \mathrm{TONO\ Score}_{N\text{-Nitrosamine}_i}\right) + \mathrm{TONO}_{\mathrm{Uncharacterized}}$$

$$= \sum_{i=1}^{3}\left(C_{N\text{-Nitrosamine}_i} \times \mathrm{SPE\ Recovery}_{N\text{-Nitrosamine}_i} \times \mathrm{Conversion\ Efficiency}_{N\text{-Nitrosamine}_i}\right)$$

$$+ \mathrm{TONO}_{\mathrm{Uncharacterized}}$$

where the summed contribution of target *N*-nitrosamines (i.e., NDMA, NMOR, and NMEA) to TONO was calculated by correcting their molar concentrations measured by LC-HRMS with their respective TONO scores.

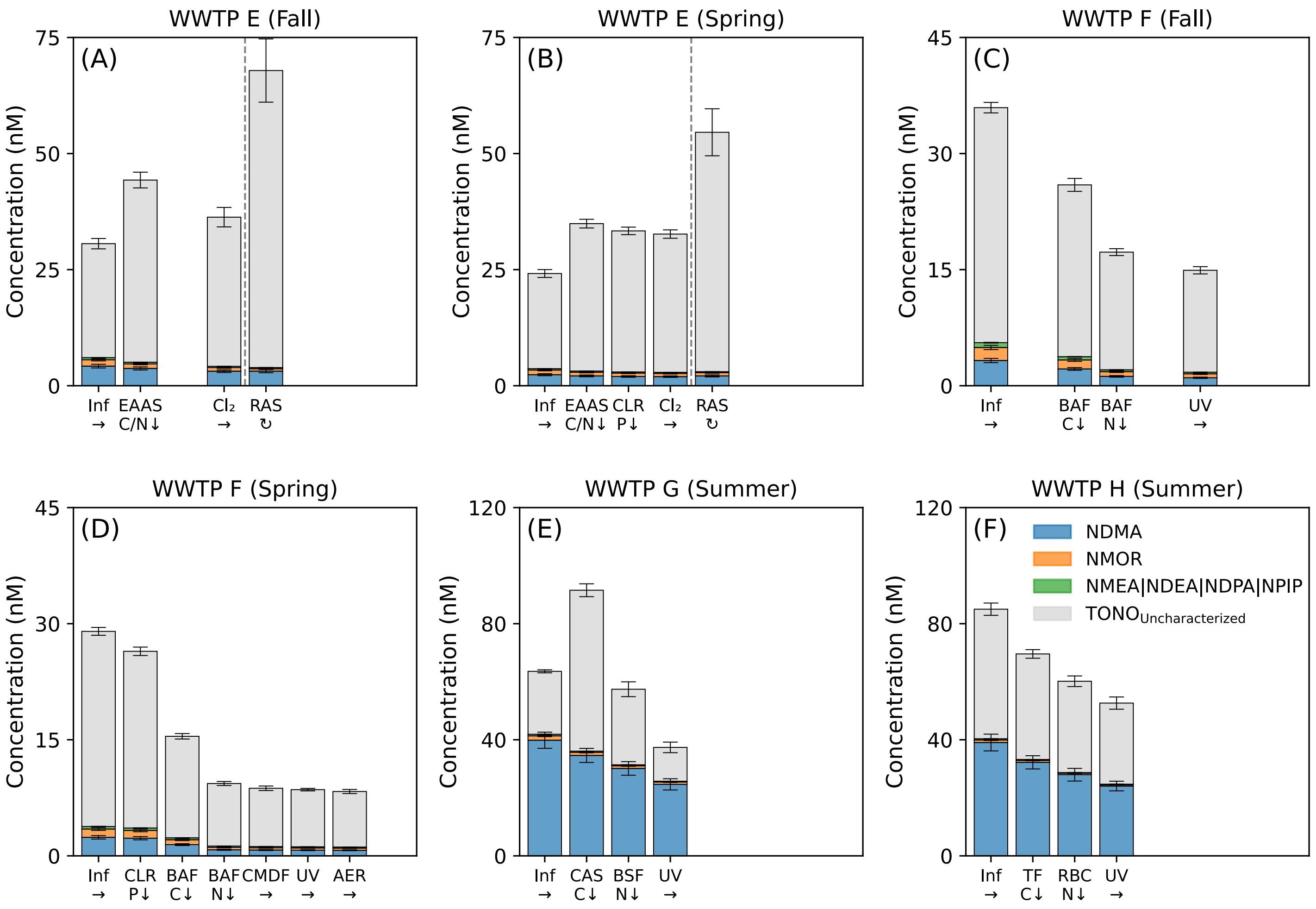

Figure S7. Concentration profiles of specific *N*-nitrosamines and TONO (i.e., the sum of specific and uncharacterized *N*-nitrosamines) for different sampling events at WWTPs E (i.e., Fall: **(A)** and Spring: **(B)**), F (i.e., Fall: **(C)** and Spring: **(D)**), G (i.e., Summer: **(E)**), and H (i.e., Summer: **(F)**). Inf = plant influent; CLR = primary or secondary clarifier effluent; CAS = conventional activated sludge aeration tank effluent; POAS = pure oxygen activated sludge aeration tank effluent; EAAS = extended aeration activated sludge aeration tank effluent; AER = post-aeration tank effluent; BAF = biological aerated filter effluent; BSF = biological sand filter effluent; TF = trickling filter effluent; RBC = rotating biological contactor effluent; HRFS = high-rate flocculated settling tank effluent; CMDF = cloth media disc filter effluent; LAG = lagoon effluent; UV = post-UV effluent; Cl_2 = post-chlorination effluent; and RAS = returned activated sludge supernatant. "→" indicates the flow direction. "↓" indicates BOD removal, nitrification, or phosphorus precipitation. "↻" indicates sludge recirculation. Error bars represent the standard deviations from duplicate measurements of specific *N*-nitrosamines and TONO. Note the differences in the *y*-axis scales. Note that for each sample, TONO measured by HI_3-CL were partitioned into specific and uncharacterized components according to the equation:

$$\text{TONO} = \text{TONO}_{\text{Specific}} + \text{TONO}_{\text{Uncharacterized}} = \sum_{i=1}^{3}\left(C_{N\text{-Nitrosamine}_i} \times \text{TONO Score}_{N\text{-Nitrosamine}_i}\right) + \text{TONO}_{\text{Uncharacterized}}$$

$$= \sum_{i=1}^{3}\left(C_{N\text{-Nitrosamine}_i} \times \text{SPE Recovery}_{N\text{-Nitrosamine}_i} \times \text{Conversion Efficiency}_{N\text{-Nitrosamine}_i}\right) + \text{TONO}_{\text{Uncharacterized}}$$

where the summed contribution of target *N*-nitrosamines (i.e., NDMA, NMOR, and NMEA) to TONO was calculated by correcting their molar concentrations measured by LC-HRMS with their respective TONO scores.

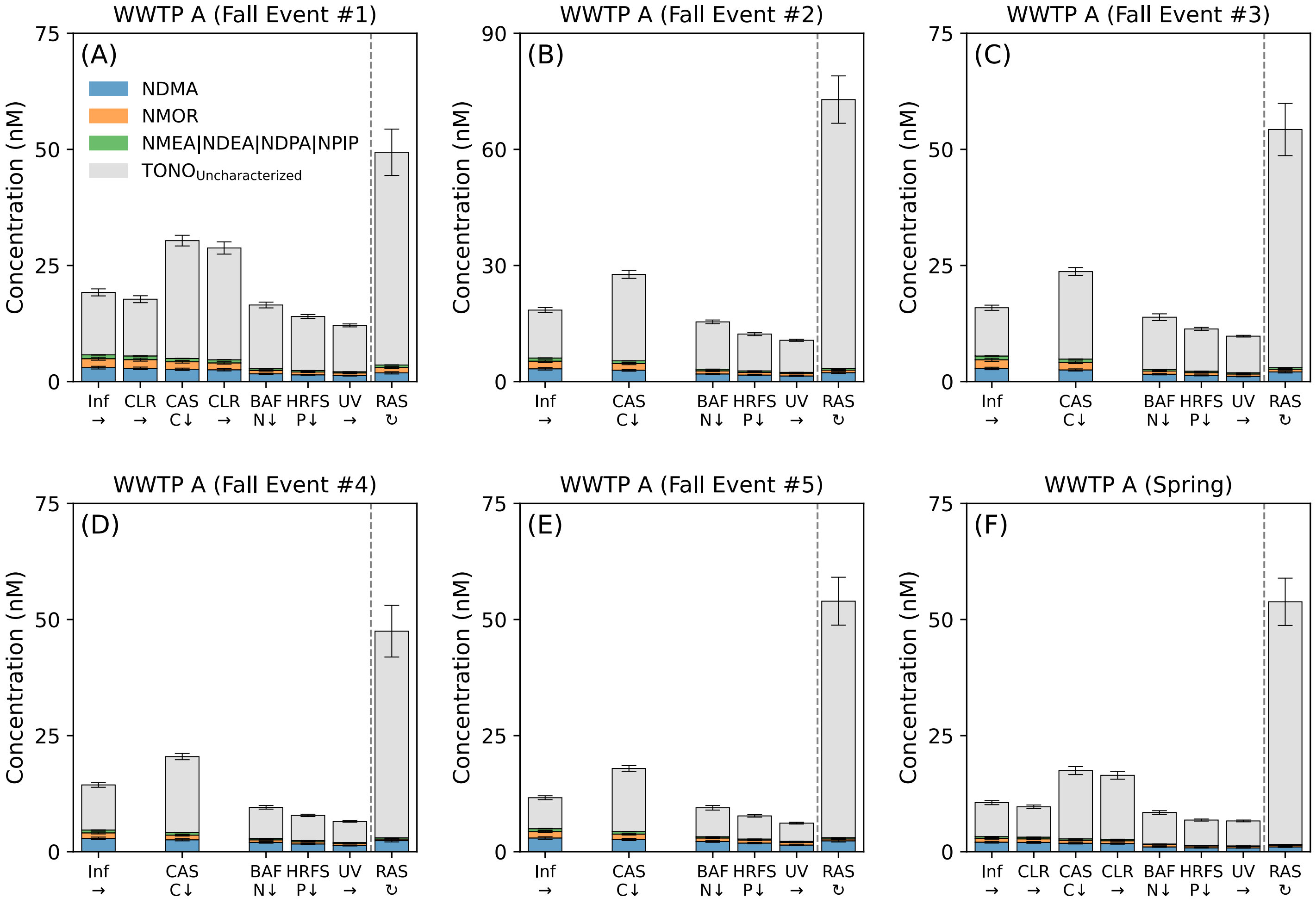

Figure S8. Concentration profiles of precursors to specific *N*-nitrosamines and TONO (i.e., the sum of specific and uncharacterized *N*-nitrosamines) for different sampling events at WWTP A (i.e., Fall: **(A)-(E)** and Spring: **(F)**). Inf = plant influent; CLR = primary or secondary clarifier effluent; CAS = conventional activated sludge aeration tank effluent; POAS = pure oxygen activated sludge aeration tank effluent; EAAS = extended aeration activated sludge aeration tank effluent; AER = post-aeration tank effluent; BAF = biological aerated filter effluent; BSF = biological sand filter effluent; TF = trickling filter effluent; RBC = rotating biological contactor effluent; HRFS = high-rate flocculated settling tank effluent; CMDF = cloth media disc filter effluent; LAG = lagoon effluent; UV = post-UV effluent; Cl_2 = post-chlorination effluent; and RAS = returned activated sludge supernatant. "→" indicates the flow direction. "↓" indicates BOD removal, nitrification, or phosphorus precipitation. "↻" indicates sludge recirculation. Error bars represent the standard deviations from duplicate measurements of precursors to specific *N*-nitrosamines and TONO. Note the differences in the *y*-axis scales. Note that for each sample, TONO measured by HI_3-CL were partitioned into specific and uncharacterized components according to the equation:

$$\text{TONO} = \text{TONO}_{\text{Specific}} + \text{TONO}_{\text{Uncharacterized}} = \sum_{i=1}^{6}\left(C_{N\text{-Nitrosamine}_i} \times \text{TONO Score}_{N\text{-Nitrosamine}_i}\right) + \text{TONO}_{\text{Uncharacterized}}$$

$$= \sum_{i=1}^{6}\left(C_{N\text{-Nitrosamine}_i} \times \text{SPE Recovery}_{N\text{-Nitrosamine}_i} \times \text{Conversion Efficiency}_{N\text{-Nitrosamine}_i}\right) + \text{TONO}_{\text{Uncharacterized}}$$

where the summed contribution of target *N*-nitrosamines (i.e., NDMA, NMOR, NMEA, NDEA, NDPA, and NPIP) to TONO was calculated by correcting their molar concentrations measured by LC-HRMS with their respective TONO scores.

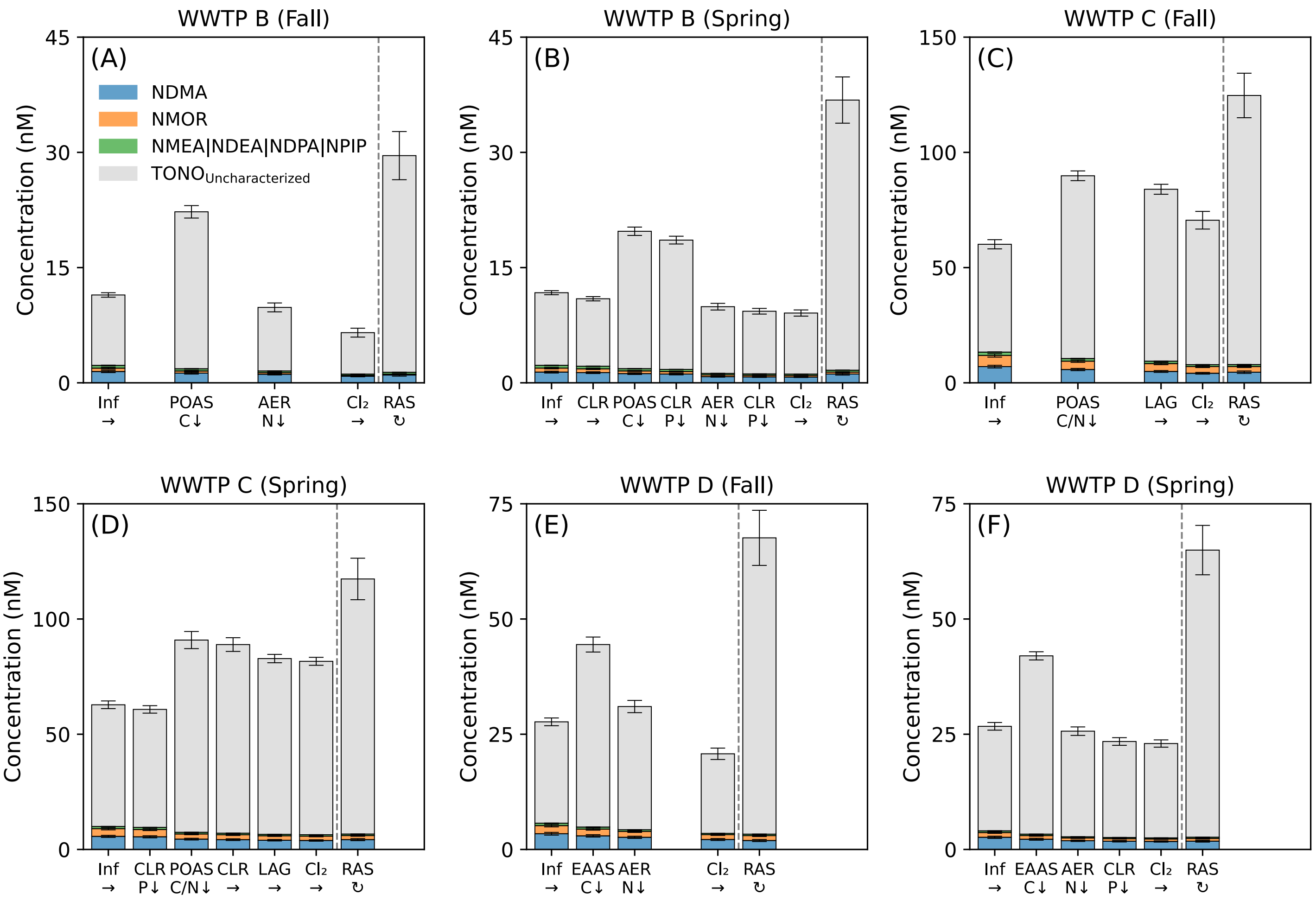

Figure S9. Concentration profiles of precursors to specific *N*-nitrosamines and TONO (i.e., the sum of specific and uncharacterized *N*-nitrosamines) for different sampling events at WWTPs B (i.e., Fall: **(A)** and Spring: **(B)**), C (i.e., Fall: **(C)** and Spring: **(D)**), and D (i.e., Fall: **(E)** and Spring: **(F)**). Inf = plant influent; CLR = primary or secondary clarifier effluent; CAS = conventional activated sludge aeration tank effluent; POAS = pure oxygen activated sludge aeration tank effluent; EAAS = extended aeration activated sludge aeration tank effluent; AER = post-aeration tank effluent; BAF = biological aerated filter effluent; BSF = biological sand filter effluent; TF = trickling filter effluent; RBC = rotating biological contactor effluent; HRFS = high-rate flocculated settling tank effluent; CMDF = cloth media disc filter effluent; LAG = lagoon effluent; UV = post-UV effluent; Cl_2 = post-chlorination effluent; and RAS = returned activated sludge supernatant. "→" indicates the flow direction. "↓" indicates BOD removal, nitrification, or phosphorus precipitation. "↻" indicates sludge recirculation. Error bars represent the standard deviations from duplicate measurements of precursors to specific *N*-nitrosamines and TONO. Note the differences in the *y*-axis scales. Note that for each sample, TONO measured by HI_3-CL were partitioned into specific and uncharacterized components according to the equation:

$$\mathrm{TONO} = \mathrm{TONO}_{\mathrm{Specific}} + \mathrm{TONO}_{\mathrm{Uncharacterized}} = \sum_{i=1}^{6}\left(C_{N\text{-Nitrosamine}_i} \times \mathrm{TONO\ Score}_{N\text{-Nitrosamine}_i}\right) + \mathrm{TONO}_{\mathrm{Uncharacterized}}$$

$$= \sum_{i=1}^{6}\left(C_{N\text{-Nitrosamine}_i} \times \mathrm{SPE\ Recovery}_{N\text{-Nitrosamine}_i} \times \mathrm{Conversion\ Efficiency}_{N\text{-Nitrosamine}_i}\right) + \mathrm{TONO}_{\mathrm{Uncharacterized}}$$

where the summed contribution of target *N*-nitrosamines (i.e., NDMA, NMOR, NMEA, NDEA, NDPA, and NPIP) to TONO was calculated by correcting their molar concentrations measured by LC-HRMS with their respective TONO scores.

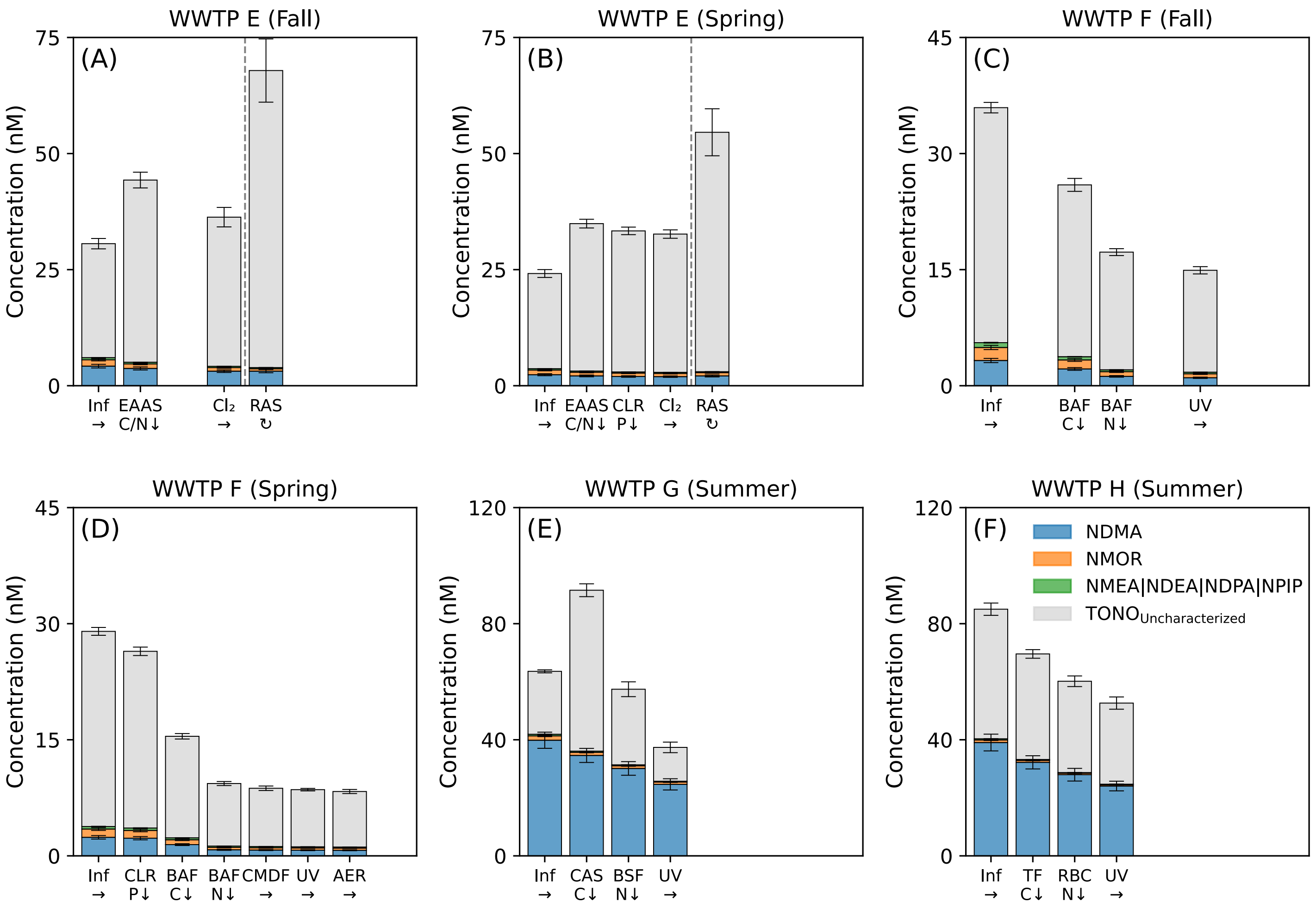

Figure S10. Concentration profiles of precursors to specific *N*-nitrosamines and TONO (i.e., the sum of specific and uncharacterized *N*-nitrosamines) for different sampling events at WWTPs E (i.e., Fall: **(A)** and Spring: **(B)**), F (i.e., Fall: **(C)** and Spring: **(D)**), G (i.e., Summer: **(E)**), and H (i.e., Summer: **(F)**). Inf = plant influent; CLR = primary or secondary clarifier effluent; CAS = conventional activated sludge aeration tank effluent; POAS = pure oxygen activated sludge aeration tank effluent; EAAS = extended aeration activated sludge aeration tank effluent; AER = post-aeration tank effluent; BAF = biological aerated filter effluent; BSF = biological sand filter effluent; TF = trickling filter effluent; RBC = rotating biological contactor effluent; HRFS = high-rate flocculated settling tank effluent; CMDF = cloth media disc filter effluent; LAG = lagoon effluent; UV = post-UV effluent; Cl_2 = post-chlorination effluent; and RAS = returned activated sludge supernatant. "→" indicates the flow direction. "↓" indicates BOD removal, nitrification, or phosphorus precipitation. "↻" indicates sludge recirculation. Error bars represent the standard deviations from duplicate measurements of precursors to specific *N*-nitrosamines and TONO. Note the differences in the *y*-axis scales. Note that for each sample, TONO measured by HI_3-CL were partitioned into specific and uncharacterized components according to the equation:

$$\text{TONO} = \text{TONO}_{\text{Specific}} + \text{TONO}_{\text{Uncharacterized}} = \sum_{i=1}^{6}\left(C_{N\text{-Nitrosamine}_i} \times \text{TONO Score}_{N\text{-Nitrosamine}_i}\right) + \text{TONO}_{\text{Uncharacterized}}$$

$$= \sum_{i=1}^{6}\left(C_{N\text{-Nitrosamine}_i} \times \text{SPE Recovery}_{N\text{-Nitrosamine}_i} \times \text{Conversion Efficiency}_{N\text{-Nitrosamine}_i}\right) + \text{TONO}_{\text{Uncharacterized}}$$

where the summed contribution of target *N*-nitrosamines (i.e., NDMA, NMOR, NMEA, NDEA, NDPA, and NPIP) to TONO was calculated by correcting their molar concentrations measured by LC-HRMS with their respective TONO scores.

8. Covariation of TONO formation potential with organic nitrogen

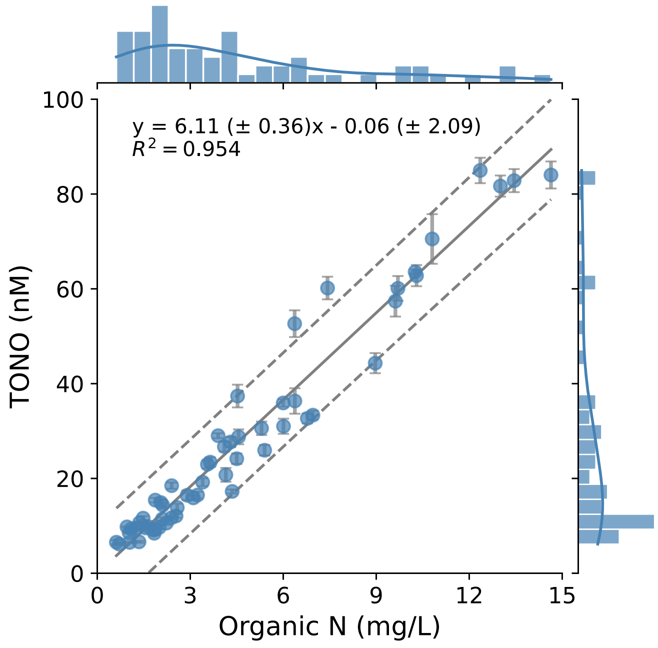

Figure S11. Linear regression between TONO formation potential and organic nitrogen in wastewater samples from WWTPs A-H. The grey solid line represents the linear regression line. The grey dashed lines bracket the 95% confidence interval of the linear regression line. Error bars represent the standard deviations from duplicate measurements; where absent, bars fall within symbols.

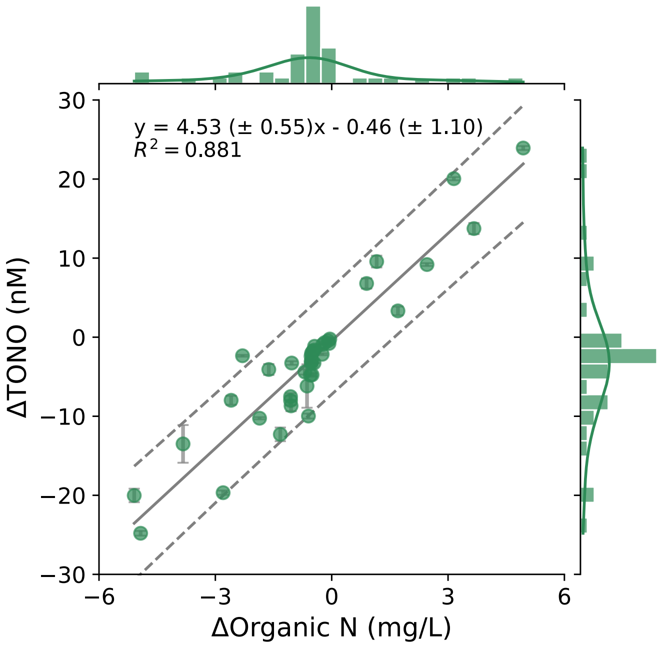

Figure S12. Linear regression between changes in TONO formation potential and variations in organic nitrogen across major treatment steps at WWTPs A-H. The grey solid line represents the linear regression line. The grey dashed lines bracket the 95% confidence interval of the linear regression line. Error bars represent the standard deviations from duplicate measurements; where absent, bars fall within symbols.

9. Concentration ranges of pharmaceuticals and other wastewater-derived substances

Table S9. Concentration ranges of pharmaceuticals and other wastewater-derived substances in selected wastewater samples

Compound Name	Category	WWTP A				WWTP G				WWTP H			
		Min (ng/L)	Mean (ng/L)	Median (ng/L)	Max (ng/L)	Min (ng/L)	Mean (ng/L)	Median (ng/L)	Max (ng/L)	Min (ng/L)	Mean (ng/L)	Median (ng/L)	Max (ng/L)
10-Hydroxy-Amitriptyline	Antidepressant	3	8	5	21	5	11	8	22	21	26	25	32
Amitriptyline	Antidepressant	<19	<19	<19	<19	<19	<19	<19	<19	45	53	52	61
Bupropion	Antidepressant	10	48	55	80	70	123	120	181	182	443	238	817
Citalopram	Antidepressant	17	45	25	106	181	235	221	316	298	539	567	715
Desvenlafaxine	Antidepressant	149	331	334	484	2071	2802	2926	3284	863	1048	1010	1371
Duloxetine	Antidepressant	<7	<7	<7	<7	<7	<7	<7	<7	<7	<7	<7	<7
Fluoxetine	Antidepressant	<5	<5	<5	<5	<5	<5	<5	<5	43	53	53	63
Fluvoxamine	Antidepressant	<27	<27	<27	<27	<27	<27	<27	<27	<27	<27	<27	<27
Norfluoxetine	Antidepressant	<40	<40	<40	<40	<40	<40	<40	<40	41	60	64	78
Norsertraline	Antidepressant	<40	<40	<40	<40	<40	<40	<40	<40	101	113	113	125
Norverapamil	Antidepressant	<4	<4	<4	<4	<4	<4	<4	<4	9	23	28	32
Paroxetine	Antidepressant	<132	<132	<132	<132	<132	<132	<132	<132	<132	<132	<132	<132
Sertraline	Antidepressant	<3	<3	<3	<3	<3	<3	<3	<3	18	64	35	131
Venlafaxine	Antidepressant	86	194	194	294	1306	1709	1770	1993	678	864	777	1155
Chlorpheniramine	Antihistamine	<27	<27	<27	<27	27	40	39	53	31	41	43	48
Diphenhydramine	Antihistamine	98	222	161	504	36	352	75	1222	294	445	455	533
Fexofenadine	Antihistamine	232	846	859	1313	2699	2955	2951	3221	4176	6114	5312	8390
Hydroxyzine	Antihistamine	2	4	4	8	<2	<2	<2	<2	2	8	7	13
Loratadine	Antihistamine	2	3	3	4	<1	<1	<1	<1	3	4	4	4
Promethazine	Antihistamine	<20	<20	<20	<20	<20	<20	<20	<20	52	58	59	62
Abacavir	Antiviral	10	21	23	29	<4	<4	<4	<4	5	9	8	13
Acyclovir	Antiviral	436	2098	1397	4917	672	1642	1143	3608	1149	3858	4585	6585
Lamivudine	Antiviral	83	234	167	477	<3	<3	<3	<3	<3	<3	<3	<3
Nevirapine	Antiviral	<145	<145	<145	<145	<145	<145	<145	<145	<145	<145	<145	<145
Oseltamivir	Antiviral	<3	<3	<3	<3	<3	<3	<3	<3	<3	<3	<3	<3
Penciclovir	Antiviral	63	106	119	153	499	499	499	499	77	123	96	197
Valacyclovir	Antiviral	<33	<33	<33	<33	<33	<33	<33	<33	<33	<33	<33	<33
Atenolol	Beta-Blocker/Heart	153	376	326	715	1018	1801	1086	4013	1034	2383	2907	3536
Clonidine	Beta-Blocker/Heart	<30	<30	<30	<30	<30	<30	<30	<30	<30	<30	<30	<30
Dehydronifedipine	Beta-Blocker/Heart	<15	<15	<15	<15	<15	<15	<15	<15	<15	<15	<15	<15
Desmethyldiltiazem	Beta-Blocker/Heart	<35	<35	<35	<35	28	35	35	43	41	54	54	64
Diltiazem	Beta-Blocker/Heart	20	60	64	103	745	1704	1029	4012	160	256	268	322
Ezetimibe	Beta-Blocker/Heart	<80	<80	<80	<80	<80	<80	<80	<80	205	208	208	211
Fenofibrate	Beta-Blocker/Heart	<7	<7	<7	<7	<7	<7	<7	<7	11	12	12	14
Metoprolol	Beta-Blocker/Heart	141	279	291	398	690	924	888	1231	1055	2057	1990	3229
Nadalol	Beta-Blocker/Heart	11	14	12	21	<10	<10	<10	<10	14	21	22	29
Pentoxifylline	Beta-Blocker/Heart	<5	<5	<5	<5	<5	<5	<5	<5	8	22	22	35
Propranolol	Beta-Blocker/Heart	13	26	20	81	96	127	130	152	55	75	73	101
Verapamil	Beta-Blocker/Heart	<70	<70	<70	<70	<70	<70	<70	<70	<70	<70	<70	<70

Compound Name	Category	WWTP A				WWTP G				WWTP H			
		Min (ng/L)	Mean (ng/L)	Median (ng/L)	Max (ng/L)	Min (ng/L)	Mean (ng/L)	Median (ng/L)	Max (ng/L)	Min (ng/L)	Mean (ng/L)	Median (ng/L)	Max (ng/L)
Cimetidine	Diabetic/Ulcer/Antacid	26	51	40	91	<21	<21	<21	<21	76	92	80	133
Famotidine	Diabetic/Ulcer/Antacid	30	55	47	95	76	140	102	280	332	469	464	622
Glipizide	Diabetic/Ulcer/Antacid	<40	<40	<40	<40	<40	<40	<40	<40	<40	<40	<40	<40
Glyburide	Diabetic/Ulcer/Antacid	<29	<29	<29	<29	<29	<29	<29	<29	<29	<29	<29	<29
Guanylurea	Diabetic/Ulcer/Antacid	411	526	507	686	<400	<400	<400	<400	3794	8804	8804	13814
Metformin	Diabetic/Ulcer/Antacid	3003	17788	18592	41913	1227	23397	1461	89440	8514	59322	83391	97191
Nizatidine	Diabetic/Ulcer/Antacid	<40	<40	<40	<40	<40	<40	<40	<40	<40	<40	<40	<40
(Es)Omeprazole	Diabetic/Ulcer/Antacid	<8	<8	<8	<8	33	33	33	33	<8	<8	<8	<8
Ranitidine	Diabetic/Ulcer/Antacid	139	342	389	528	1056	1856	1587	3193	6702	9936	11149	12186
Sitagliptin	Diabetic/Ulcer/Antacid	103	220	202	331	39	45	45	51	377	628	704	783
Codeine	Opiate	52	64	65	71	308	39359	39359	78410	58	79	82	100
Hydrocodone	Opiate	11	19	18	26	2130	5600	3845	12578	68	77	73	87
Loperamide	Opiate	<40	<40	<40	<40	<40	<40	<40	<40	<40	<40	<40	<40
Methadone	Opiate	13	35	42	67	20079	23363	22682	28006	43	48	45	59
Morphine	Opiate	31	193	199	289	452	42787	795	169107	99	426	428	783
Oxycodone	Opiate	16	32	31	57	1354	9436	3644	29104	110	148	123	201
Propoxyphene	Opiate	<3	<3	<3	<3	41	44	44	47	<3	<3	<3	<3
Tramadol	Opiate	86	204	232	274	190	232	245	250	332	468	498	642
Acetaminophen	Other Pharmaceutical	11	13456	21943	31241	36	83617	60	334314	149	57316	90692	104382
Albuterol	Other Pharmaceutical	3	7	6	11	8	11	9	18	11	19	21	29
Alprazolam	Other Pharmaceutical	<7	<7	<7	<7	<7	<7	<7	<7	<7	<7	<7	<7
Antipyrine	Other Pharmaceutical	<58	<58	<58	<58	<58	<58	<58	<58	<58	<58	<58	<58
Benztropine	Other Pharmaceutical	<22	<22	<22	<22	<22	<22	<22	<22	<22	<22	<22	<22
Betamethasone	Other Pharmaceutical	<57	<57	<57	<57	<57	<57	<57	<57	<57	<57	<57	<57
Carbamazepine	Other Pharmaceutical	45	124	135	170	272	322	322	373	463	552	545	613
Carisoprodol	Other Pharmaceutical	<25	<25	<25	<25	<25	<25	<25	<25	<25	<25	<25	<25
Erythromycin	Other Pharmaceutical	<27	<27	<27	<27	<27	<27	<27	<27	<27	<27	<27	<27
Fadrozole	Other Pharmaceutical	<6	<6	<6	<6	<6	<6	<6	<6	<6	<6	<6	<6
Fluconazole	Other Pharmaceutical	61	132	118	208	54	60	58	69	349	611	562	840
Fluticasone	Other Pharmaceutical	<1	<1	<1	<1	<1	<1	<1	<1	<1	<1	<1	<1
Gabapentin	Other Pharmaceutical	2577	14164	9203	42061	<400	<400	<400	<400	12480	38613	51381	56254
Hexamethylenetetramine	Other Pharmaceutical	43	407	471	837	<40	<40	<40	<40	43	147	147	250
Hydrocortisone	Other Pharmaceutical	96	96	96	96	<73	<73	<73	<73	166	185	183	206
Iminostilbene	Other Pharmaceutical	<73	<73	<73	<73	<73	<73	<73	<73	<73	<73	<73	<73
Ketoconazole	Other Pharmaceutical	<56	<56	<56	<56	<56	<56	<56	<56	<56	<56	<56	<56
Lidocaine	Other Pharmaceutical	75	246	265	353	470	582	584	689	437	882	695	1452
Meprobamate	Other Pharmaceutical	17	27	27	37	<17	<17	<17	<17	102	141	138	199
Metaxalone	Other Pharmaceutical	23	110	117	198	555	656	668	732	<8	<8	<8	<8
Methocarbamol	Other Pharmaceutical	71	148	138	207	557	633	629	716	251	317	299	471
Methotrexate	Other Pharmaceutical	<26	<26	<26	<26	<26	<26	<26	<26	76	81	81	86
Norethindrone	Other Pharmaceutical	<10	<10	<10	<10	<10	<10	<10	<10	<10	<10	<10	<10
Phenazopyridine	Other Pharmaceutical	<4	<4	<4	<4	<4	<4	<4	<4	<4	<4	<4	<4

Table S9. Concentration ranges of pharmaceuticals and other wastewater-derived substances in selected wastewater samples (continued)

**Table S9.** Concentration ranges of pharmaceuticals and other wastewater-derived substances in selected wastewater samples (continued)													
Compound Name	Category	WWTP A				WWTP G				WWTP H			
		Min (ng/L)	Mean (ng/L)	Median (ng/L)	Max (ng/L)	Min (ng/L)	Mean (ng/L)	Median (ng/L)	Max (ng/L)	Min (ng/L)	Mean (ng/L)	Median (ng/L)	Max (ng/L)
Phendimetrazine	Other Pharmaceutical	<16	<16	<16	<16	<16	<16	<16	<16	<16	<16	<16	<16
Phenytoin	Other Pharmaceutical	<94	<94	<94	<94	<94	<94	<94	<94	119	167	159	215
Prednisolone	Other Pharmaceutical	<75	<75	<75	<75	<75	<75	<75	<75	<75	<75	<75	<75
Prednisone	Other Pharmaceutical	<84	<84	<84	<84	<84	<84	<84	<84	<84	<84	<84	<84
Quinine	Other Pharmaceutical	20	29	26	50	25	47	47	69	89	177	141	295
Raloxifene	Other Pharmaceutical	<40	<40	<40	<40	<40	<40	<40	<40	<40	<40	<40	<40
Sulfadimethoxine	Other Pharmaceutical	<33	<33	<33	<33	<33	<33	<33	<33	<33	<33	<33	<33
Sulfamethizole	Other Pharmaceutical	<21	<21	<21	<21	<21	<21	<21	<21	<21	<21	<21	<21
Sulfamethoxazole	Other Pharmaceutical	49	436	485	875	1430	2304	1752	4283	443	682	713	800
Tamoxifen	Other Pharmaceutical	<270	<270	<270	<270	<270	<270	<270	<270	<270	<270	<270	<270
Theophylline	Other Pharmaceutical	330	851	678	1525	1776	1776	1776	1776	283	2778	3450	3929
Thiabendazole	Other Pharmaceutical	6	14	16	21	9	10	10	11	8	14	15	18
Tiotropium	Other Pharmaceutical	<100	<100	<100	<100	<100	<100	<100	<100	<100	<100	<100	<100
Triamterene	Other Pharmaceutical	4	12	14	18	35	62	66	83	27	34	32	44
Trimethoprim	Other Pharmaceutical	97	169	164	294	77	375	144	1135	253	520	491	741
Warfarin	Other Pharmaceutical	3	3	3	3	6	6	6	7	7	8	8	9
Amphetamine	Stimulant/Abuse	18	57	52	104	16	11808	23	47170	6	158	188	249
Dextromethorphan	Stimulant/Abuse	10	24	19	43	14	21	19	30	67	279	76	628
Diazepam	Stimulant/Abuse	<2	<2	<2	<2	2	3	3	3	3	7	6	12
Lorazepam	Stimulant/Abuse	<101	<101	<101	<101	<101	<101	<101	<101	<101	<101	<101	<101
Nordiazepam	Stimulant/Abuse	<10	<10	<10	<10	<10	<10	<10	<10	<10	<10	<10	<10
Oxazepam	Stimulant/Abuse	<113	<113	<113	<113	<113	<113	<113	<113	<113	<113	<113	<113
Pseudo(Ephedrine)	Stimulant/Abuse	17	254	99	753	24	311	62	1095	119	1529	2209	2685
Temazepam	Stimulant/Abuse	<9	<9	<9	<9	5078	7200	7224	9275	11	20	20	30
1,7-Dimethylxanthine	Caffeine/Nicotine	41	2569	2055	6017	<21	<21	<21	<21	606	20585	28343	38749
Caffeine	Caffeine/Nicotine	70	10906	6309	30486	32	43543	85	130512	2196	102497	142202	180608
Cotinine	Caffeine/Nicotine	46	414	270	1099	33	582	57	2183	124	912	1040	1678
Nicotine	Caffeine/Nicotine	43	1246	1325	1831	4382	4382	4382	4382	116	2075	2922	3746
Atrazine	Pesticide	10	24	24	44	<10	<10	<10	<10	17	19	20	22
Methyl-1H-Benzotriazole	Other	549	4301	4064	12048	30787	36283	33501	47341	9291	21457	19452	34853
Piperonyl Butoxide	Other	80	124	128	164	137	137	137	137	<10	<10	<10	<10
Ractopamine	Other	<40	<40	<40	<40	<40	<40	<40	<40	<40	<40	<40	<40

10. Confirmation of nontargeted *N*-nitrosamines by reference standards in wastewater samples

Table S10. Confirmation of *N*-nitrosonornicotine in wastewater by its reference standard

Molecular Structure	Molecular Formula	Wastewater Sample			Reference Standard		
		ΔMass [ppm]	*m/z*	RT [min]	ΔMass [ppm]	*m/z*	RT [min]
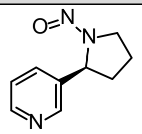	$[C_9H_{11}N_3O+H]+$	1.18	178.0977	5.70	-3.87	178.0968	5.74
Fragment Ion	Formula	ΔMass [ppm]	*m/z*	Fragment Ion	Formula	ΔMass [ppm]	*m/z*
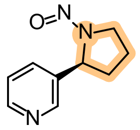	$[C_4H_8N]+$	-3.14	70.0649	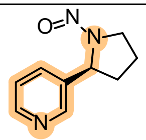	$[C_6H_5N_2]+$	-1.14	105.0446
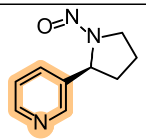	$[C_5H_5N]+$	-3.80	79.0419	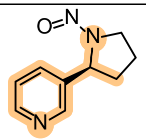	$[C_7H_8N_2]+$	-1.96	120.0686
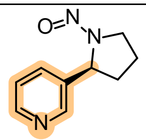	$[C_6H_7N]+$	4.84	93.0583	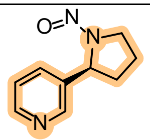	$[C_9H_{12}N_2]+$ (NO Loss)	-4.39	148.0994

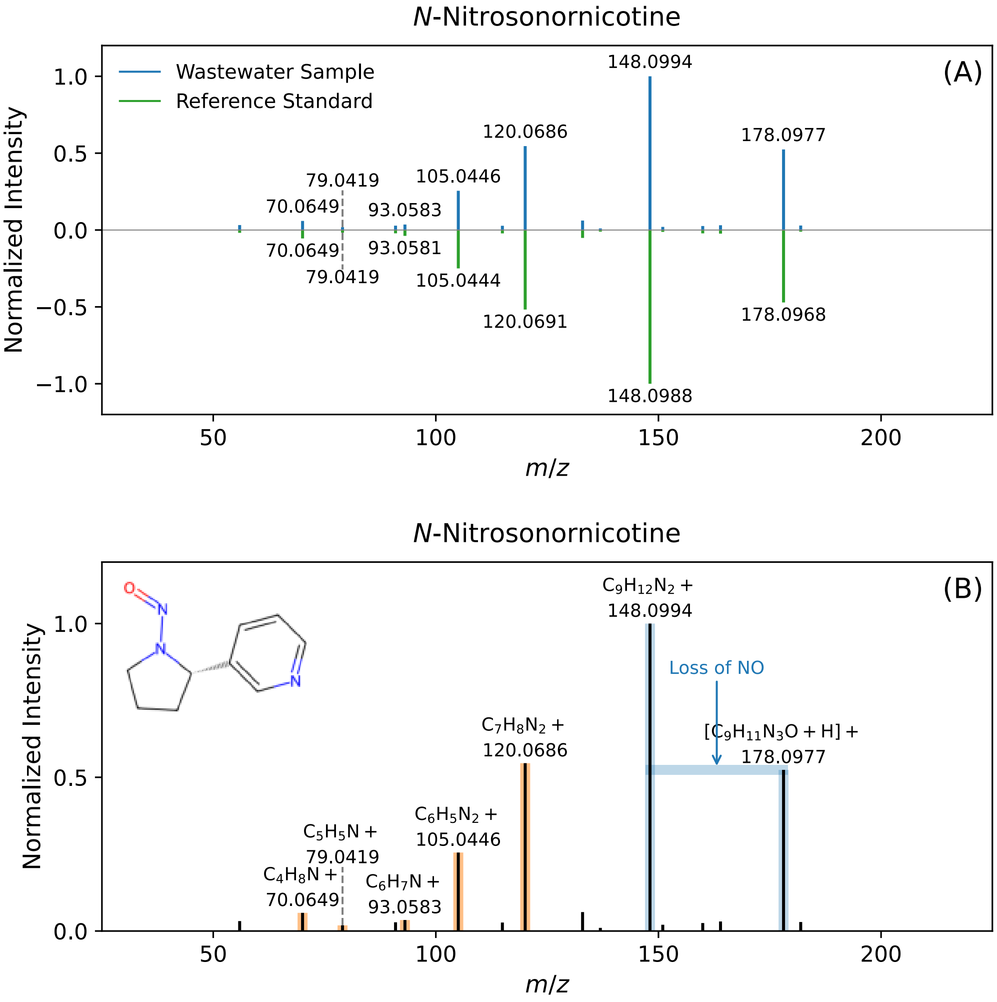

Figure S13. Confirmation of *N*-nitrosonornicotine in wastewater: **(A)** Head-to-tail plots of the dd-MS2 spectra of *N*-nitrosonornicotine acquired from the wastewater sample (top) and its reference standard (bottom). **(B)** Characteristic fragment ions highlighted in the dd-MS2 spectrum of *N*-nitrosonornicotine acquired from the wastewater sample (Table S10).

**Table S11.** Confirmation of 4-(methylnitrosamino)-1-(3-pyridyl)-1-butanone in wastewater by its reference standard							
Molecular Structure	Molecular Formula	Wastewater Sample			Reference Standard		
		ΔMass [ppm]	*m/z*	RT [min]	ΔMass [ppm]	*m/z*	RT [min]
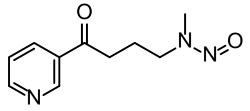	$[C_{10}H_{13}N_3O_2+H]+$	-2.64	208.1075	14.30	-1.68	208.1077	14.28
Fragment Ion	Formula	ΔMass [ppm]	*m/z*	Fragment Ion	Formula	ΔMass [ppm]	*m/z*
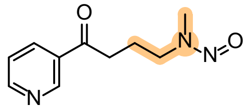	$[C_3H_7N]+$	-4.38	57.0576	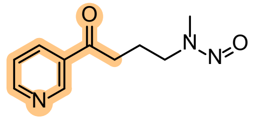	$[C_7H_8NO]+$	-1.15	122.0599
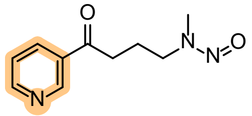	$[C_5H_5N]+$	3.80	79.0425	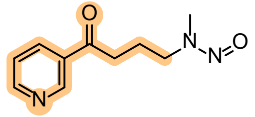	$[C_9H_{10}NO]+$	-1.96	148.0754
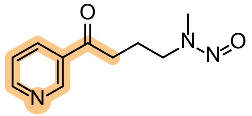	$[C_7H_8N]+$	-3.02	106.0648	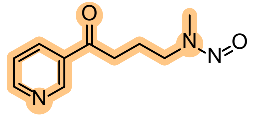	$[C_{10}H_{14}N_2O]+$ (NO Loss)	-4.55	178.1098

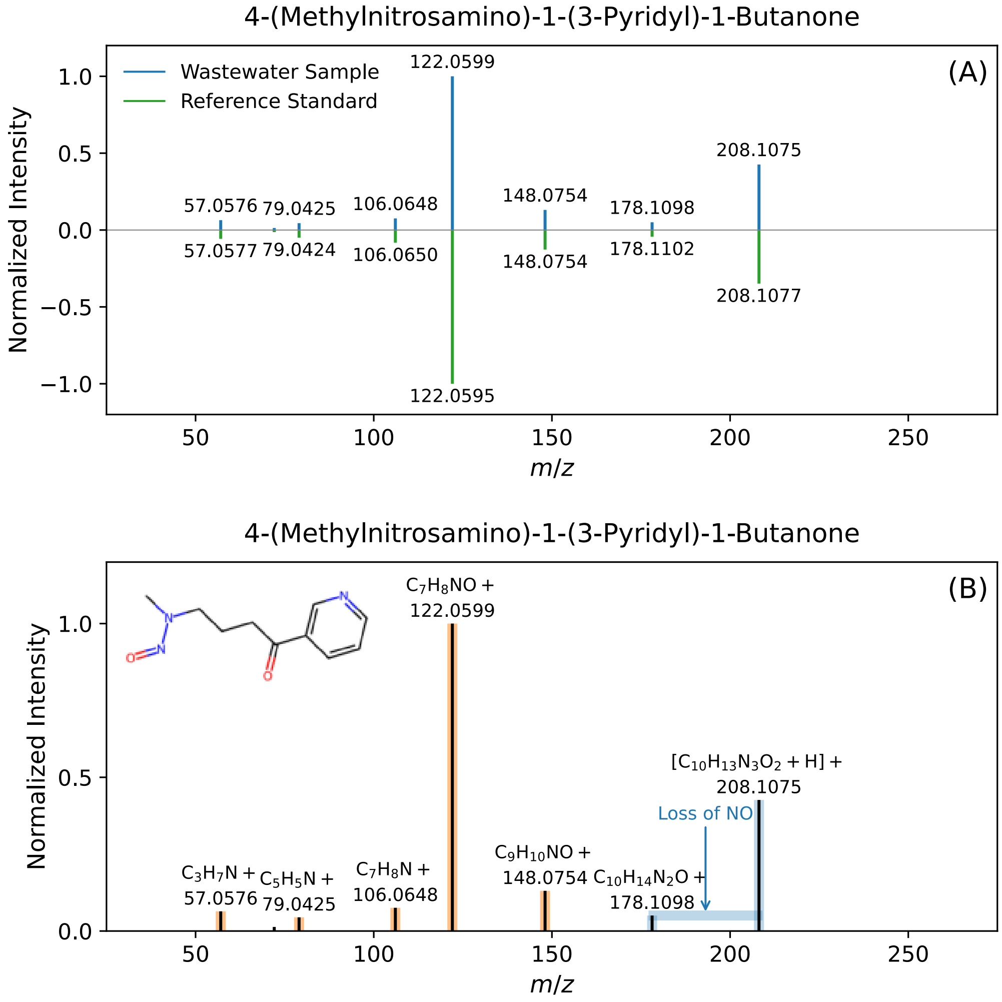

Figure S14. Confirmation of 4-(methylnitrosamino)-1-(3-pyridyl)-1-butanone in wastewater: **(A)** Head-to-tail plots of the dd-MS2 spectra of 4-(methylnitrosamino)-1-(3-pyridyl)-1-butanone acquired from the wastewater sample (top) and its reference standard (bottom). **(B)** Characteristic fragment ions highlighted in the dd-MS2 spectrum of 4-(methylnitrosamino)-1-(3-pyridyl)-1-butanone acquired from the wastewater sample (Table S11).

Table S12. Confirmation of 4-(methylnitrosamino)-1-(3-pyridyl)-1-butanol in wastewater by its reference standard

Molecular Structure	Molecular Formula	Wastewater Sample			Reference Standard		
		ΔMass [ppm]	*m/z*	RT [min]	ΔMass [ppm]	*m/z*	RT [min]
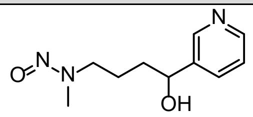	$[C_{10}H_{15}N_3O_2 +H]+$	-0.95	210.1235	5.00	0.00	210.1237	4.90
Fragment Ion	Formula	ΔMass [ppm]	*m/z*	Fragment Ion	Formula	ΔMass [ppm]	*m/z*
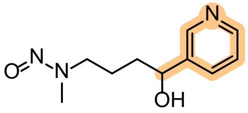	$[C_6H_7N]+$	4.84	93.0583	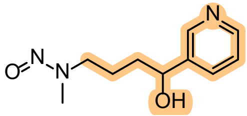	$[C_9H_{11}NO]+$	-0.40	149.0840
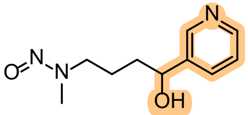	$[C_6H_7NO]+$	4.03	109.0532	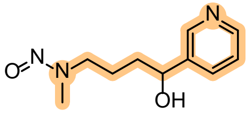	$[C_{10}H_{14}N_2]+$	-1.85	162.1154
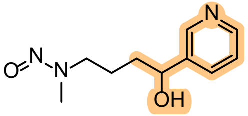	$[C_7H_7NO]+$	2.81	121.0531	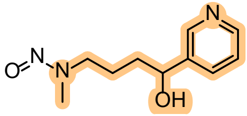	$[C_{10}H_{16}N_2O]+$ (NO Loss)	-0.89	180.1261

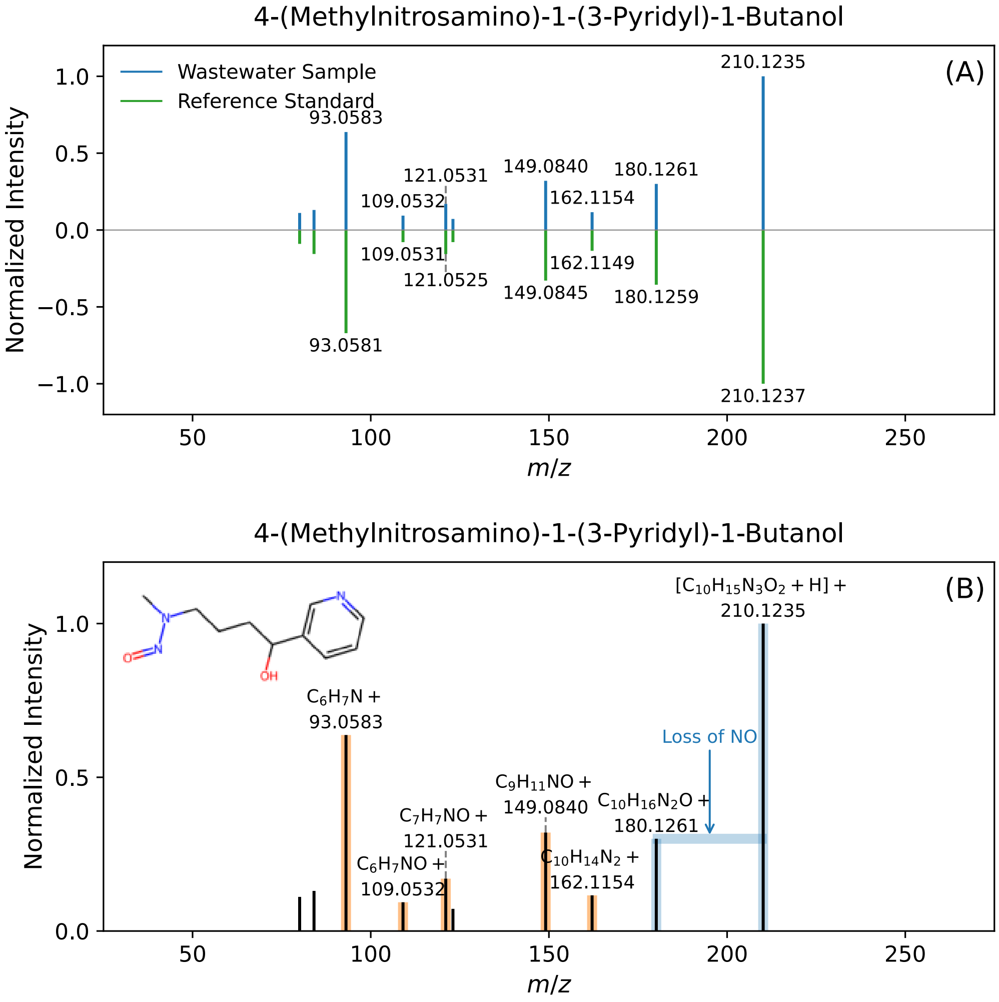

Figure S15. Confirmation of 4-(methylnitrosamino)-1-(3-pyridyl)-1-butanol in wastewater: **(A)** Head-to-tail plots of the dd-MS2 spectra of 4-(methylnitrosamino)-1-(3-pyridyl)-1-butanol acquired from the wastewater sample (top) and its reference standard (bottom). **(B)** Characteristic fragment ions highlighted in the dd-MS2 spectrum of 4-(methylnitrosamino)-1-(3-pyridyl)-1-butanol acquired from the wastewater sample (Table S12).

Table S13. Confirmation of *N*-nitrosopiperazine in wastewater by its reference standard

Molecular Structure	Molecular Formula	Wastewater Sample			Reference Standard		
		ΔMass [ppm]	*m/z*	RT [min]	ΔMass [ppm]	*m/z*	RT [min]
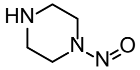	$[C_4H_9N_3O+H]+$	1.38	116.0820	2.00	3.10	116.0822	1.96
Fragment Ion	Formula	ΔMass [ppm]	*m/z*	Fragment Ion	Formula	ΔMass [ppm]	*m/z*
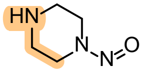	$[C_2H_6N]+$	0.68	44.0495	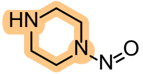	$[C_4H_{10}N_2]+$ (NO Loss)	0.00	86.0844

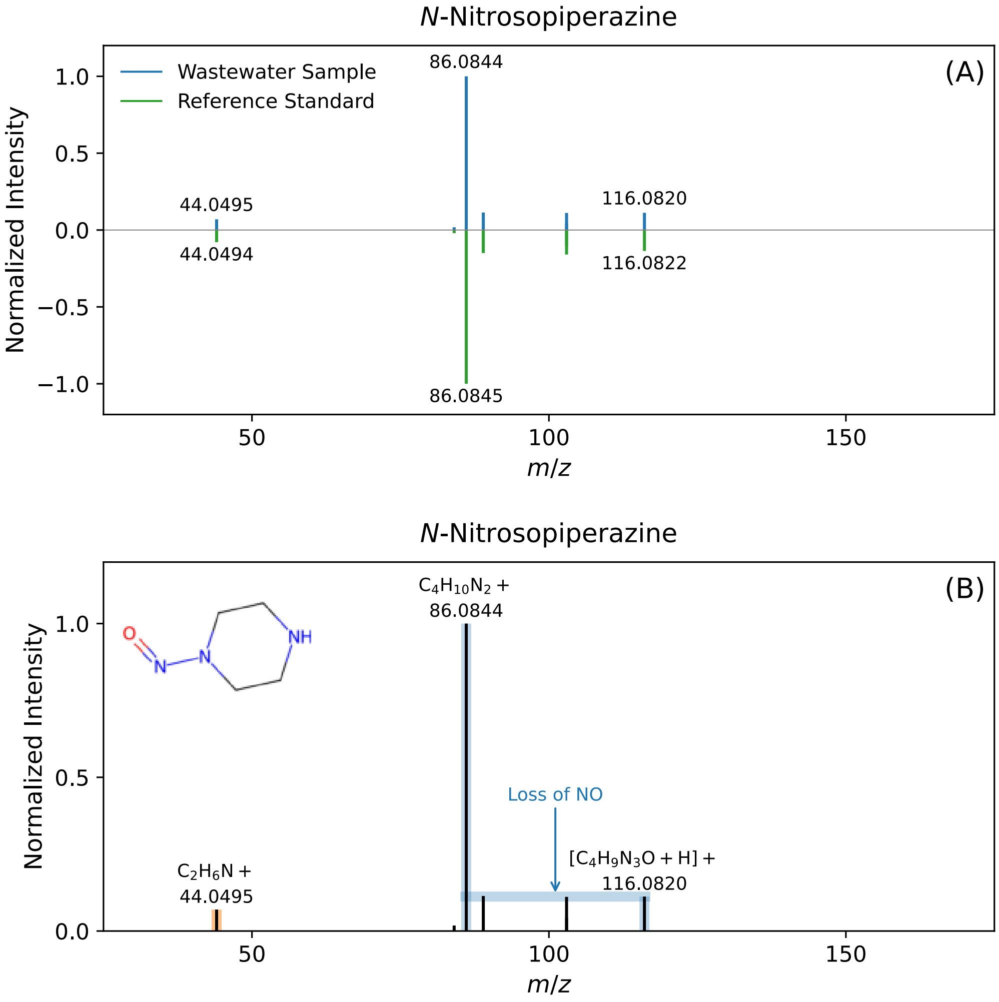

Figure S16. Confirmation of *N*-nitrosopiperazine in wastewater: **(A)** Head-to-tail plots of the dd-MS2 spectra of *N*-nitrosopiperazine acquired from the wastewater sample (top) and its reference standard (bottom). **(B)** Characteristic fragment ions highlighted in the dd-MS2 spectrum of *N*-nitrosopiperazine acquired from the wastewater sample (Table S13).

Table S14. Confirmation of *N*-nitroso-*tert*-butylphenylamine in wastewater by its reference standard

Molecular Structure	Molecular Formula	Wastewater Sample			Reference Standard		
		ΔMass [ppm]	*m/z*	RT [min]	ΔMass [ppm]	*m/z*	RT [min]
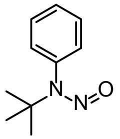	$[C_{10}H_{14}N_2O+H]+$	4.52	179.1187	16.70	4.52	179.1187	16.68
Fragment Ion	Formula	ΔMass [ppm]	*m/z*	Fragment Ion	Formula	ΔMass [ppm]	*m/z*
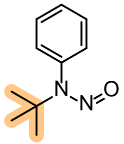	$[C_4H_9]+$	-1.40	57.0698	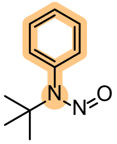	$[C_6H_7N]+$	-4.84	93.0574
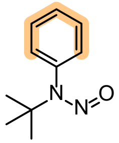	$[C_5H_6]+$	-3.79	66.0467	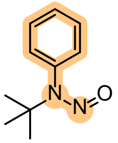	$[C_6H_5N_2]+$	2.67	105.0450
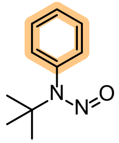	$[C_6H_5]+$	-2.34	77.0384	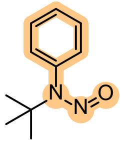	$[C_6H_7N_2O]+$	-0.73	123.0552

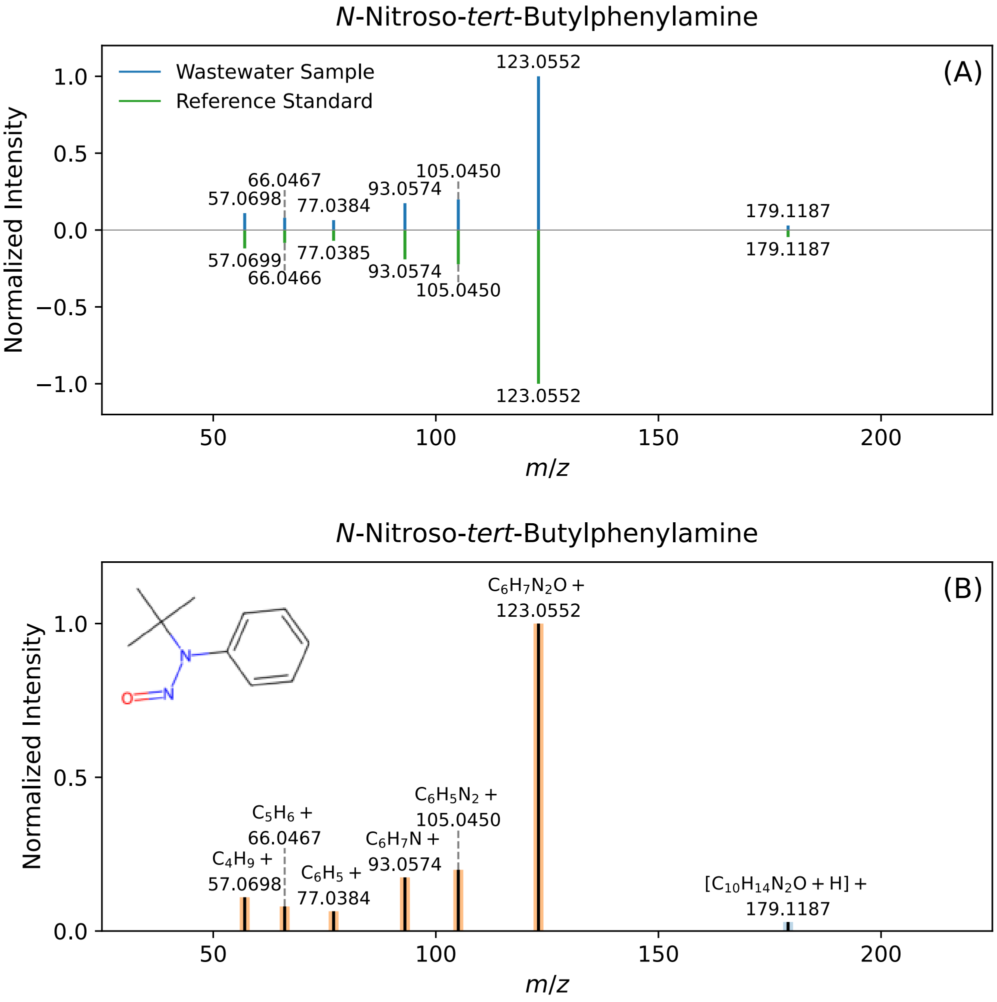

Figure S17. Confirmation of *N*-nitroso-*tert*-butylphenylamine in wastewater: **(A)** Head-to-tail plots of the dd-MS2 spectra of *N*-nitroso-*tert*-butylphenylamine acquired from the wastewater sample (top) and its reference standard (bottom). **(B)** Characteristic fragment ions highlighted in the dd-MS2 spectrum of *N*-nitroso-*tert*-butylphenylamine acquired from the wastewater sample (Table S14).

**Table S15.** Confirmation of *N*-nitroso-2-pyrrolidinmethanol in wastewater by its reference standard							
Molecular Structure	Molecular Formula	Wastewater Sample			Reference Standard		
		ΔMass [ppm]	*m/z*	RT [min]	ΔMass [ppm]	*m/z*	RT [min]
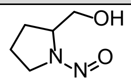	$[C_5H_{10}N_2O_2+H]^+$	-3.81	131.0810	5.75	2.29	131.0818	5.80
Fragment Ion	Formula	ΔMass [ppm]	*m/z*	Fragment Ion	Formula	ΔMass [ppm]	*m/z*
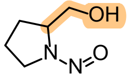	$[C_2H_3O]^+$	-3.25	43.0177	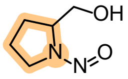	$[C_4H_8N]^+$	-3.14	70.0649
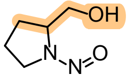	$[C_3H_5O]^+$	-3.33	57.0333	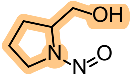	$[C_5H_{10}NO]^+$	-4.90	100.0752
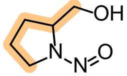	$[C_5H_7]^+$	-4.92	67.0539				

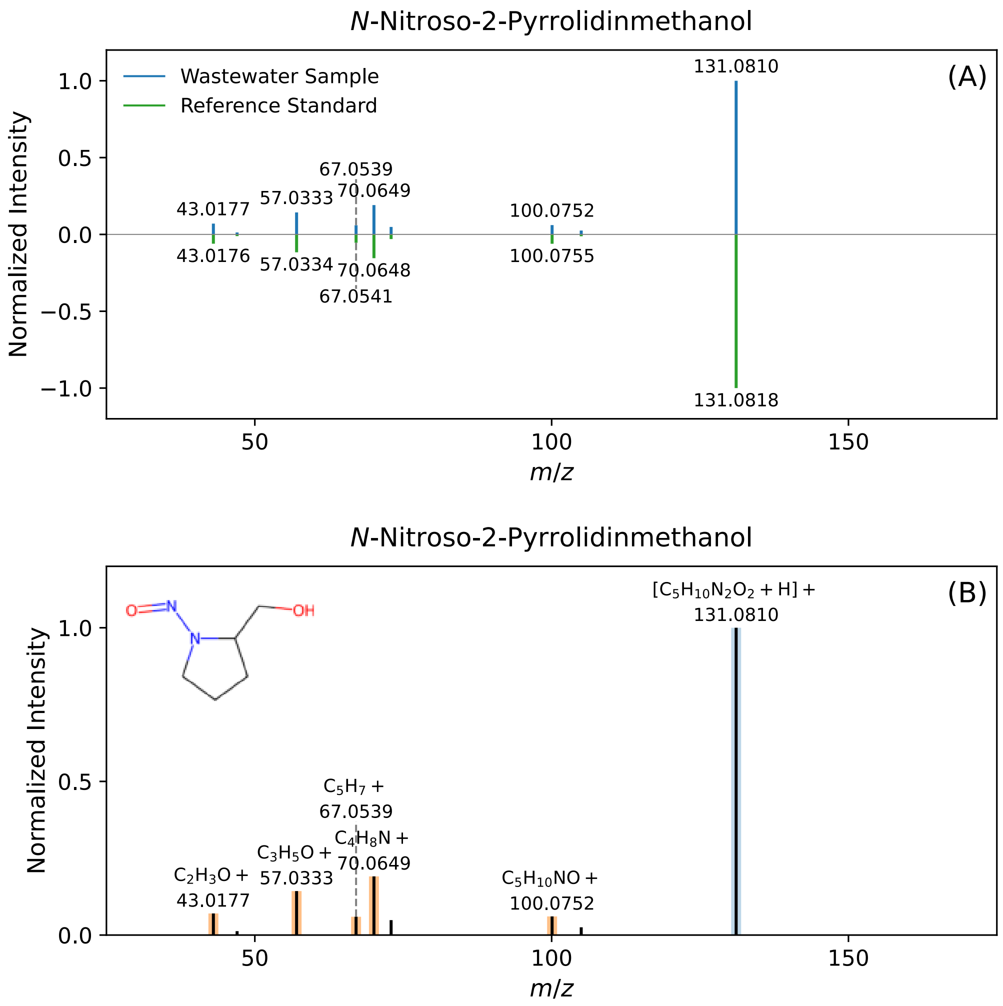

Figure S18. Confirmation of *N*-nitroso-2-pyrrolidinmethanol in wastewater: **(A)** Head-to-tail plots of the dd-MS2 spectra of *N*-nitroso-2-pyrrolidinmethanol acquired from the wastewater sample (top) and its reference standard (bottom). **(B)** Characteristic fragment ions highlighted in the dd-MS2 spectrum of *N*-nitroso-2-pyrrolidinmethanol acquired from the wastewater sample (Table S15).

Table S16. Confirmation of *N*-nitrosodesloratadine in wastewater by its reference standard

Molecular Structure	Molecular Formula	Wastewater Sample			Reference Standard		
		ΔMass [ppm]	*m/z*	RT [min]	ΔMass [ppm]	*m/z*	RT [min]
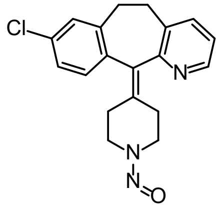	$[C_{19}H_{18}ClN_3O+H]+$	-2.68	340.1202	17.93	1.73	340.1217	17.94
Fragment Ion	Formula	ΔMass [ppm]	*m/z*	Fragment Ion	Formula	ΔMass [ppm]	*m/z*
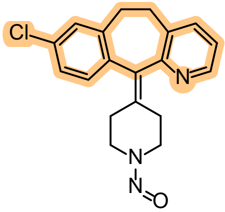	$[C_{14}H_{12}ClN]+$	-3.62	229.0650	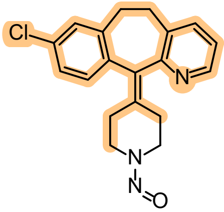	$[C_{18}H_{16}ClN]+$	-0.82	281.0969
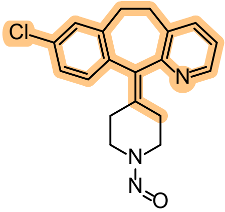	$[C_{16}H_{14}ClN]+$	-4.23	255.0804	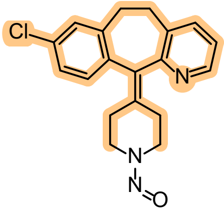	$[C_{19}H_{16}ClN]+$	1.60	293.0976
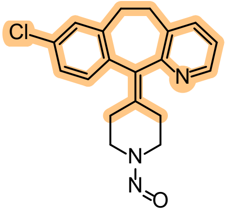	$[C_{17}H_{13}ClN]+$	-0.38	266.0730	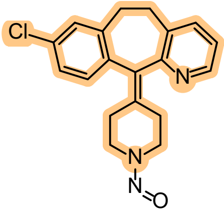	$[C_{19}H_{19}ClN_2]+$ (NO Loss)	-1.55	310.1232

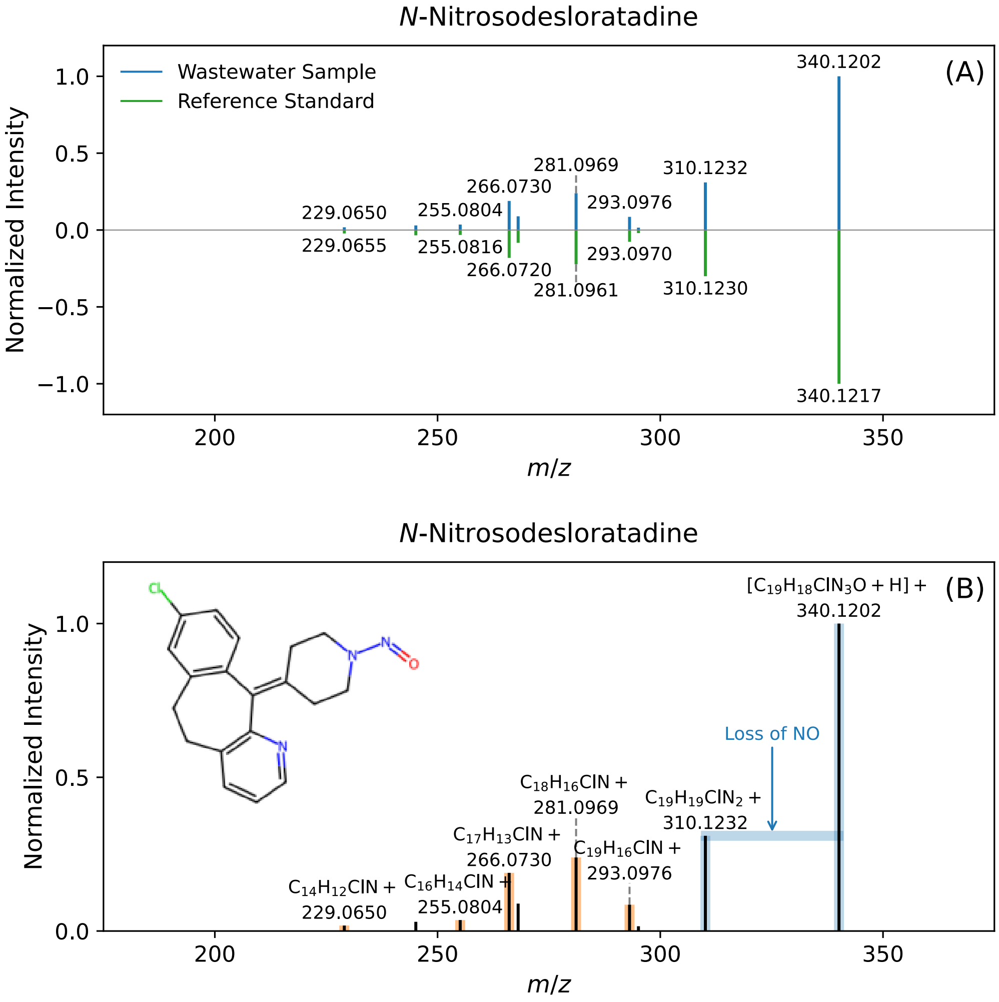

Figure S19. Confirmation of *N*-nitrosodesloratadine in wastewater: **(A)** Head-to-tail plots of the dd-MS2 spectra of *N*-nitrosodesloratadine acquired from the wastewater sample (top) and its reference standard (bottom). **(B)** Characteristic fragment ions highlighted in the dd-MS2 spectrum of *N*-nitrosodesloratadine acquired from the wastewater sample (Table S16).

11. Formation of *N*-nitroso derivatives from pharmaceuticals upon chloramination

Table S17. Confirmation of *N*-nitrosodesloratadine formation by its reference standard and ^{15}N labeling

Molecular Structure	Molecular Formula	Chloraminated Sample			Reference Standard		
		ΔMass [ppm]	*m/z*	RT [min]	ΔMass [ppm]	*m/z*	RT [min]
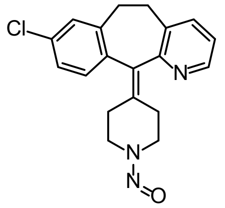	$[C_{19}H_{18}ClN_3O + H]^+$	-2.97	340.1201	18.00	1.73	340.1217	17.94
Fragment Ion	Formula	ΔMass [ppm]	*m/z*	Fragment Ion	Formula	ΔMass [ppm]	*m/z*
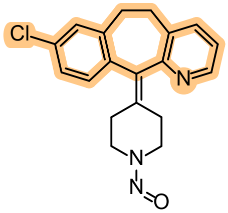	$[C_{14}H_{12}ClN]^+$	0.74	229.0660	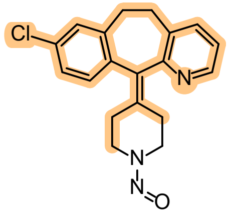	$[C_{18}H_{16}ClN]^+$	2.38	281.0978
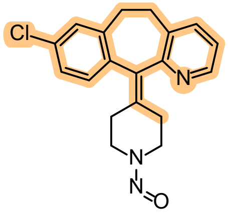	$[C_{16}H_{14}ClN]^+$	2.82	255.0822	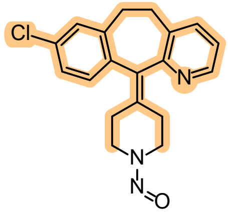	$[C_{19}H_{16}ClN]^+$	1.26	293.0975
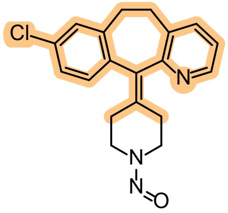	$[C_{17}H_{13}ClN]^+$	4.13	266.0742	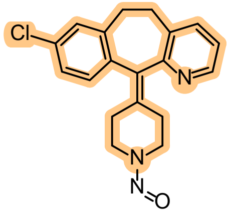	$[C_{19}H_{19}ClN_2]^+$ (NO Loss)	2.64	310.1245

Table S17. Confirmation of *N*-nitrosodesloratadine formation by its reference standard and ^{15}N labeling (continued)

Molecular Structure	Molecular Formula	^{15}N Chloraminated Sample					
		ΔMass [ppm]	*m/z*	RT [min]			
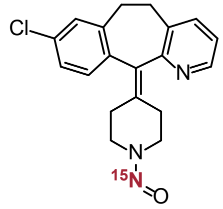	$[C_{19}H_{18}ClN_2{}^{15}NO +H]+$	-1.61	341.1176	18.07			
Fragment Ion	Formula	ΔMass [ppm]	*m/z*	Fragment Ion	Formula	ΔMass [ppm]	*m/z*
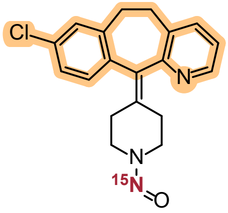	$[C_{14}H_{12}ClN]+$	-3.62	229.0650	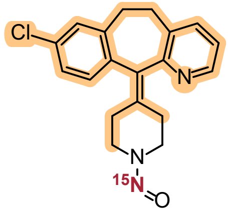	$[C_{18}H_{16}ClN]+$	-0.11	281.0971
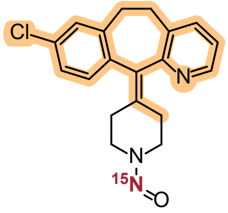	$[C_{16}H_{14}ClN]+$	-3.06	255.0807	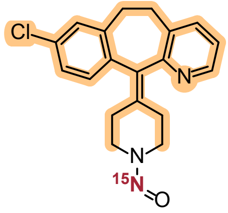	$[C_{19}H_{16}ClN]+$	-2.83	293.0963
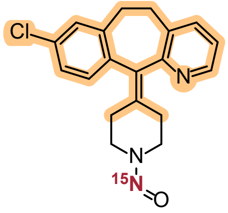	$[C_{17}H_{13}ClN]+$	-0.38	266.0730	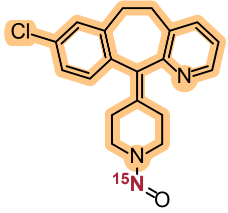	$[C_{19}H_{19}ClN_2]+$ (^{15}NO Loss)	-1.55	310.1232

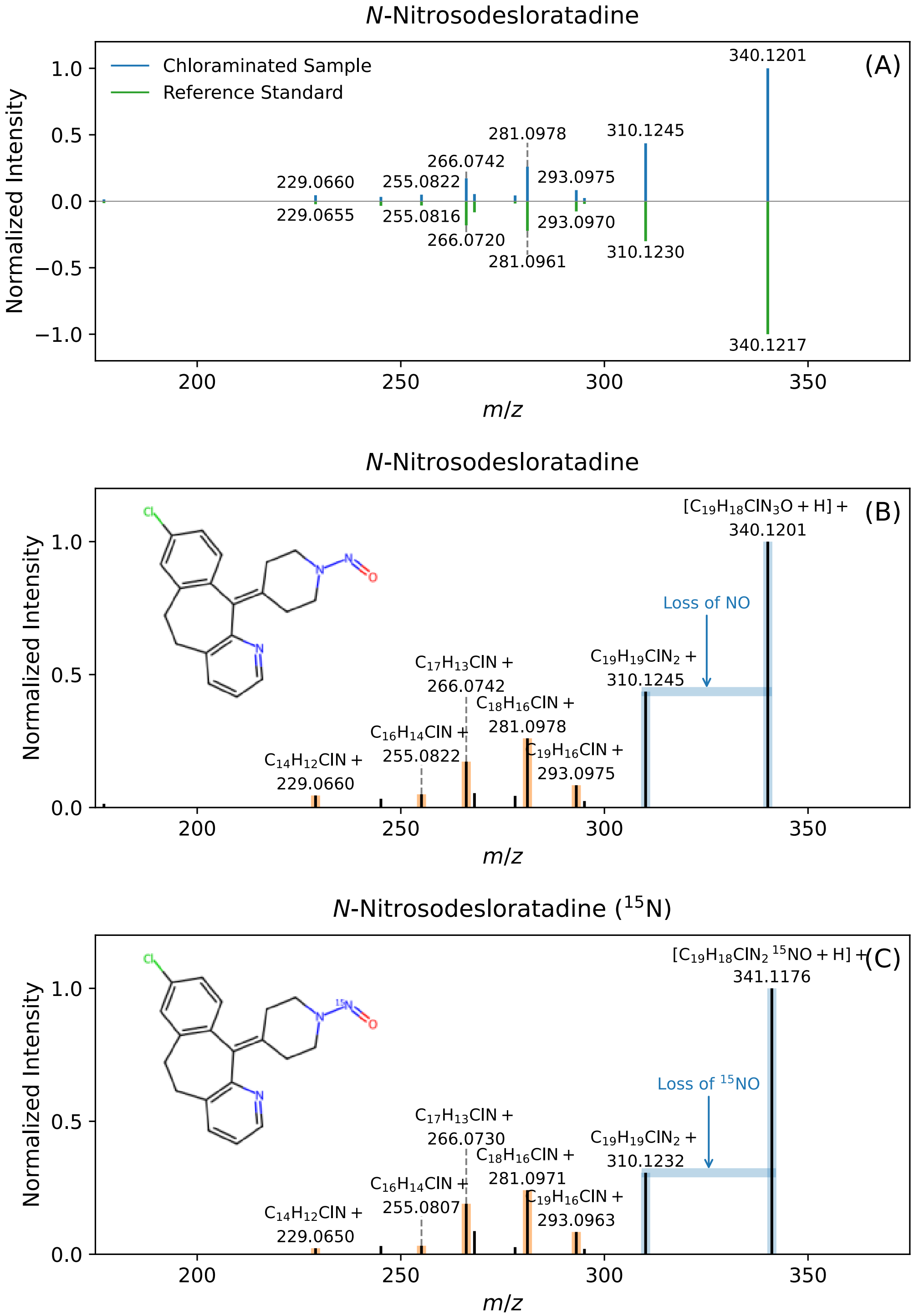

Figure S20. Confirmation of *N*-nitrosodesloratadine formation upon chloramination of desloratadine under formation potential test conditions: **(A)** Head-to-tail plots of the dd-MS2 spectra of *N*-nitrosodesloratadine acquired from the chloraminated sample (top) and its reference standard (bottom). **(B)** Characteristic fragment ions highlighted in the dd-MS2 spectrum of *N*-nitrosodesloratadine acquired from the chloraminated sample (Table S17). **(C)** Characteristic fragment ions highlighted in the dd-MS2 spectrum of *N*-nitrosodesloratadine acquired from the ^{15}N chloraminated sample (Table S17).

Table S18. Confirmation of *N*-nitrosofenfluramine formation by its reference standard and ^{15}N labeling

Molecular Structure	Molecular Formula	Chlorinated Sample			Reference Standard		
		ΔMass [ppm]	*m/z*	RT [min]	ΔMass [ppm]	*m/z*	RT [min]
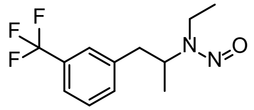	$[C_{12}H_{15}F_3N_2O+H]+$	-1.61	261.1205	17.40	-3.52	261.1200	17.40
Fragment Ion	Formula	ΔMass [ppm]	*m/z*	Fragment Ion	Formula	ΔMass [ppm]	*m/z*
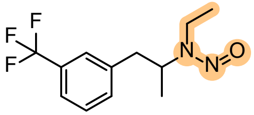	$[C_2H_7N_2O]+$	1.47	75.0554	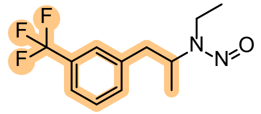	$[C_{10}H_{10}F_3]+$	-1.66	187.0726
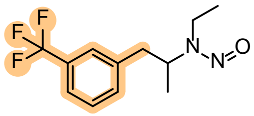	$[C_8H_6F_3]+$	-2.58	159.0412	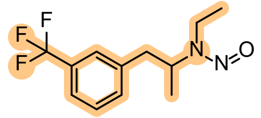	$[C_{12}H_{14}F_2N]+$	-0.86	210.1087
Molecular Structure	Molecular Formula	^{15}N Chlorinated Sample					
		ΔMass [ppm]	*m/z*	RT [min]			
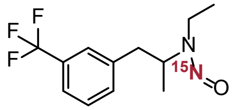	$[C_{12}H_{15}F_3N^{15}NO+H]+$	-1.37	262.1176	17.31			
Fragment Ion	Formula	ΔMass [ppm]	*m/z*	Fragment Ion	Formula	ΔMass [ppm]	*m/z*
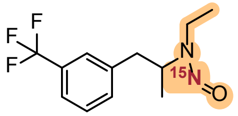	$[C_2H_7N^{15}NO]+$	-1.58	76.0522	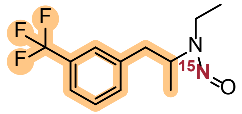	$[C_{10}H_{10}F_3]+$	-1.66	187.0726
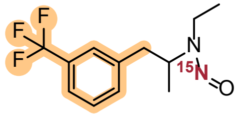	$[C_8H_6F_3]+$	0.57	159.0417	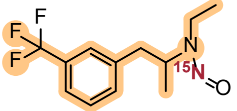	$[C_{12}H_{14}F_2N]+$	0.57	210.1090

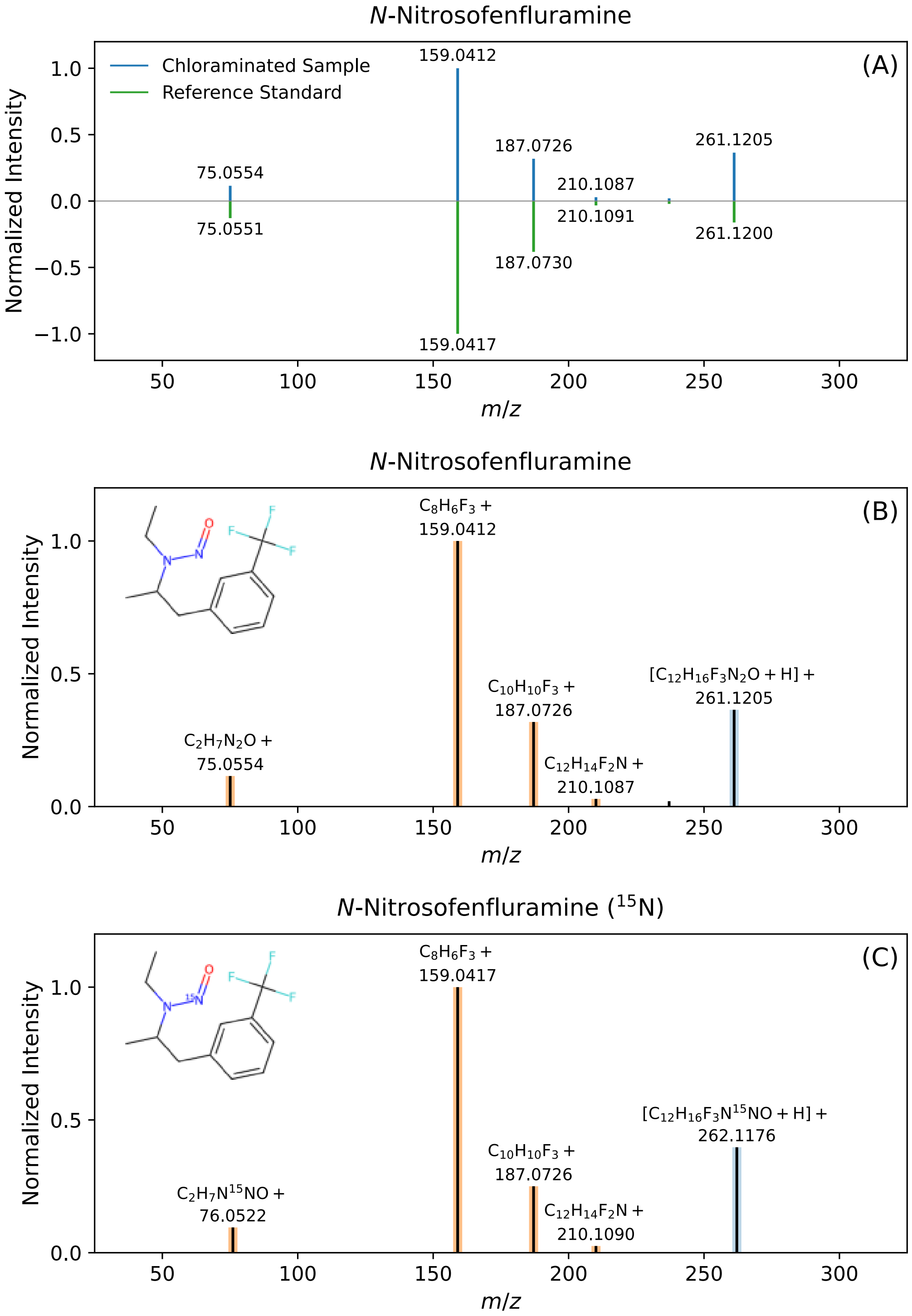

Figure S21. Confirmation of *N*-nitrosofenfluramine formation upon chloramination of fenfluramine under formation potential test conditions: **(A)** Head-to-tail plots of the dd-MS2 spectra of *N*-nitrosofenfluramine acquired from the chloraminated sample (top) and its reference standard (bottom). **(B)** Characteristic fragment ions highlighted in the dd-MS2 spectrum of *N*-nitrosofenfluramine acquired from the chloraminated sample (Table S18). **(C)** Characteristic fragment ions highlighted in the dd-MS2 spectrum of *N*-nitrosofenfluramine acquired from the ^{15}N chloraminated sample (Table S18).

**Table S19.** Confirmation of *N*-nitrosofluoxetine formation by its reference standard and ^{15}N labeling							
Molecular Structure	Molecular Formula	Chloraminated Sample			Reference Standard		
		ΔMass [ppm]	*m/z*	RT [min]	ΔMass [ppm]	*m/z*	RT [min]
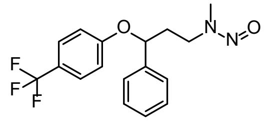	$[C_{17}H_{17}F_3N_2O_2+H]+$	1.74	339.1321	18.30	-3.51	339.1303	18.26
Fragment Ion	Formula	ΔMass [ppm]	*m/z*	Fragment Ion	Formula	ΔMass [ppm]	*m/z*
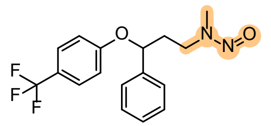	$[C_2H_5N_2O]+$	-4.66	73.0393	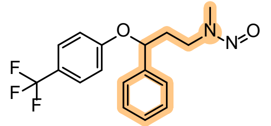	$[C_{10}H_{12}N]+$	-4.24	146.0958
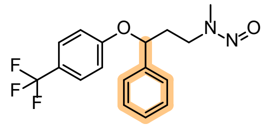	$[C_7H_7]+$	-0.33	91.0542	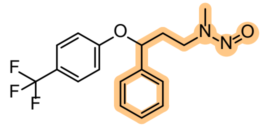	$[C_{10}H_{13}N_2O]+$	3.16	177.1028
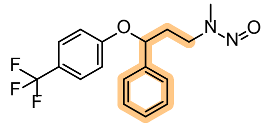	$[C_9H_9]+$	2.73	117.0702				
Molecular Structure	Molecular Formula	^{15}N Chloraminated Sample					
		ΔMass [ppm]	*m/z*	RT [min]			
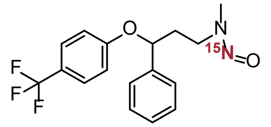	$[C_{17}H_{17}F_3N^{15}NO_2+H]+$	-1.82	340.1279	18.25			
Fragment Ion	Formula	ΔMass [ppm]	*m/z*	Fragment Ion	Formula	ΔMass [ppm]	*m/z*
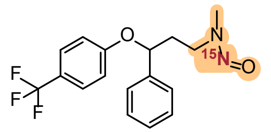	$[C_2H_5N^{15}NO]+$	0.41	74.0367	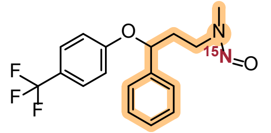	$[C_{10}H_{12}N]+$	-2.19	146.0961
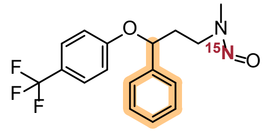	$[C_7H_7]+$	-3.62	91.0539	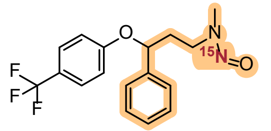	$[C_{10}H_{13}N^{15}NO]+$	0.17	178.0993
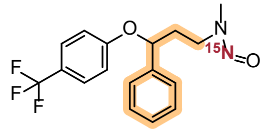	$[C_9H_9]+$	-3.25	117.0695				

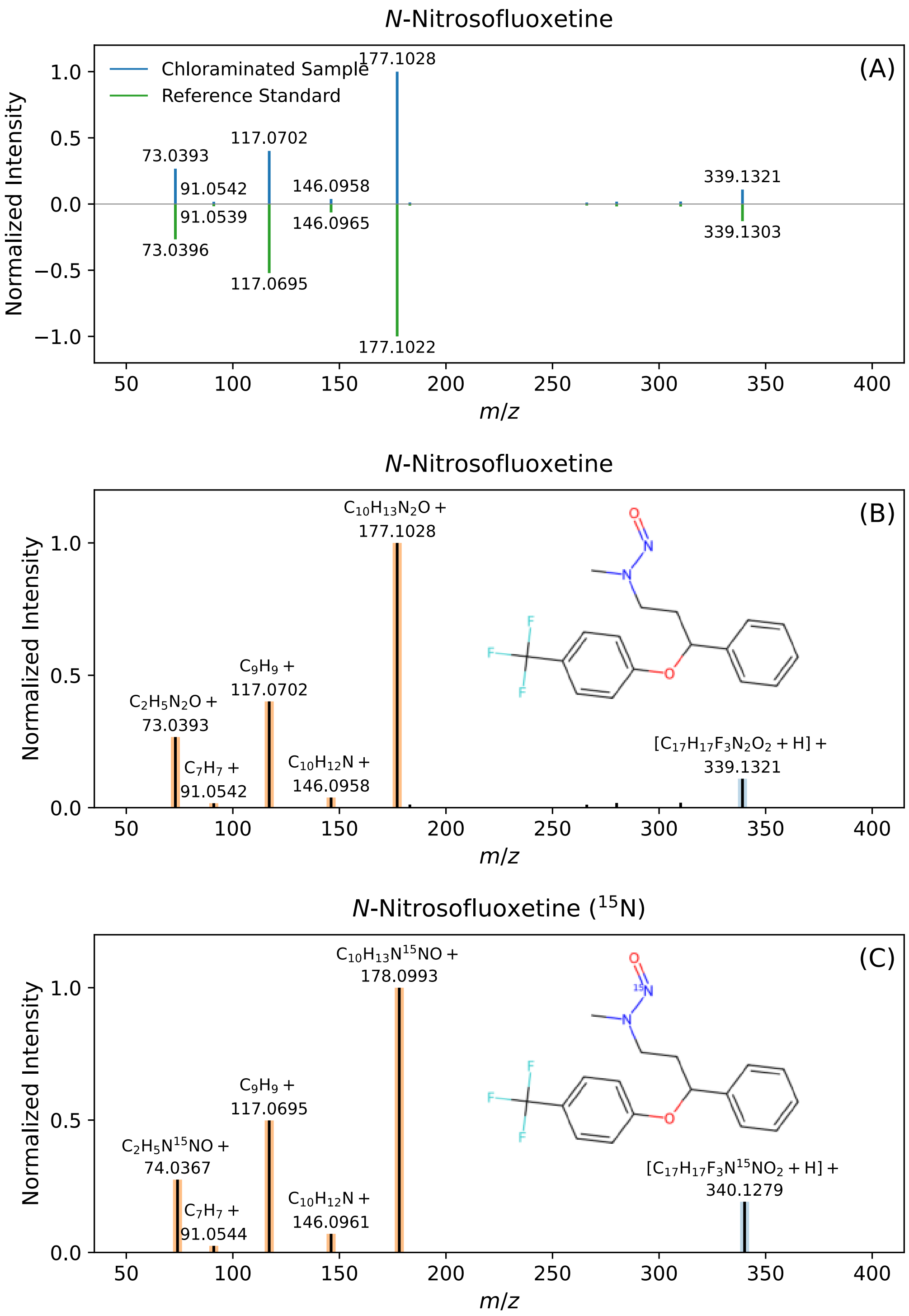

Figure S22. Confirmation of *N*-nitrosofluoxetine formation upon chloramination of fluoxetine under formation potential test conditions: **(A)** Head-to-tail plots of the dd-MS2 spectra of *N*-nitrosofluoxetine acquired from the chloraminated sample (top) and its reference standard (bottom). **(B)** Characteristic fragment ions highlighted in the dd-MS2 spectrum of *N*-nitrosofluoxetine acquired from the chloraminated sample (Table S19). **(C)** Characteristic fragment ions highlighted in the dd-MS2 spectrum of *N*-nitrosofluoxetine acquired from the ^{15}N chloraminated sample (Table S19).

Table S20. Confirmation of *N*-nitrosobetahistine formation by its reference standard and ^{15}N labeling

Molecular Structure	Molecular Formula	Chloraminated Sample			Reference Standard		
		ΔMass [ppm]	*m/z*	RT [min]	ΔMass [ppm]	*m/z*	RT [min]
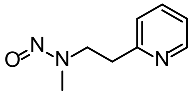	$[C_8H_{11}N_3O+H]+$	1.87	166.0978	3.90	-3.55	166.0969	3.86
Fragment Ion	Formula	ΔMass [ppm]	*m/z*	Fragment Ion	Formula	ΔMass [ppm]	*m/z*
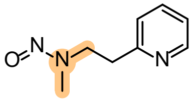	$[C_2H_6N]+$	-3.86	44.0493	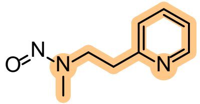	$[C_8H_{11}N_2]+$ (NO Loss)	-1.10	136.0999
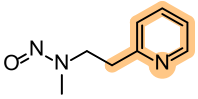	$[C_6H_7N]+$	-3.76	93.0575				
Molecular Structure	Molecular Formula	^{15}N Chloraminated Sample					
		ΔMass [ppm]	*m/z*	RT [min]			
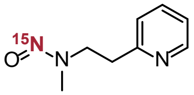	$[C_8H_{12}N_2{}^{15}NO+H]+$	-2.51	167.0941	3.98			
Fragment Ion	Formula	ΔMass [ppm]	*m/z*	Fragment Ion	Formula	ΔMass [ppm]	*m/z*
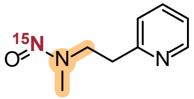	$[C_2H_6N]+$	0.68	44.0495	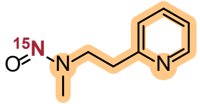	$[C_8H_{11}N_2]+$ (^{15}NO Loss)	-4.04	136.0995
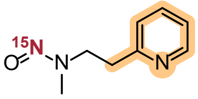	$[C_6H_7N]+$	0.54	93.0579				

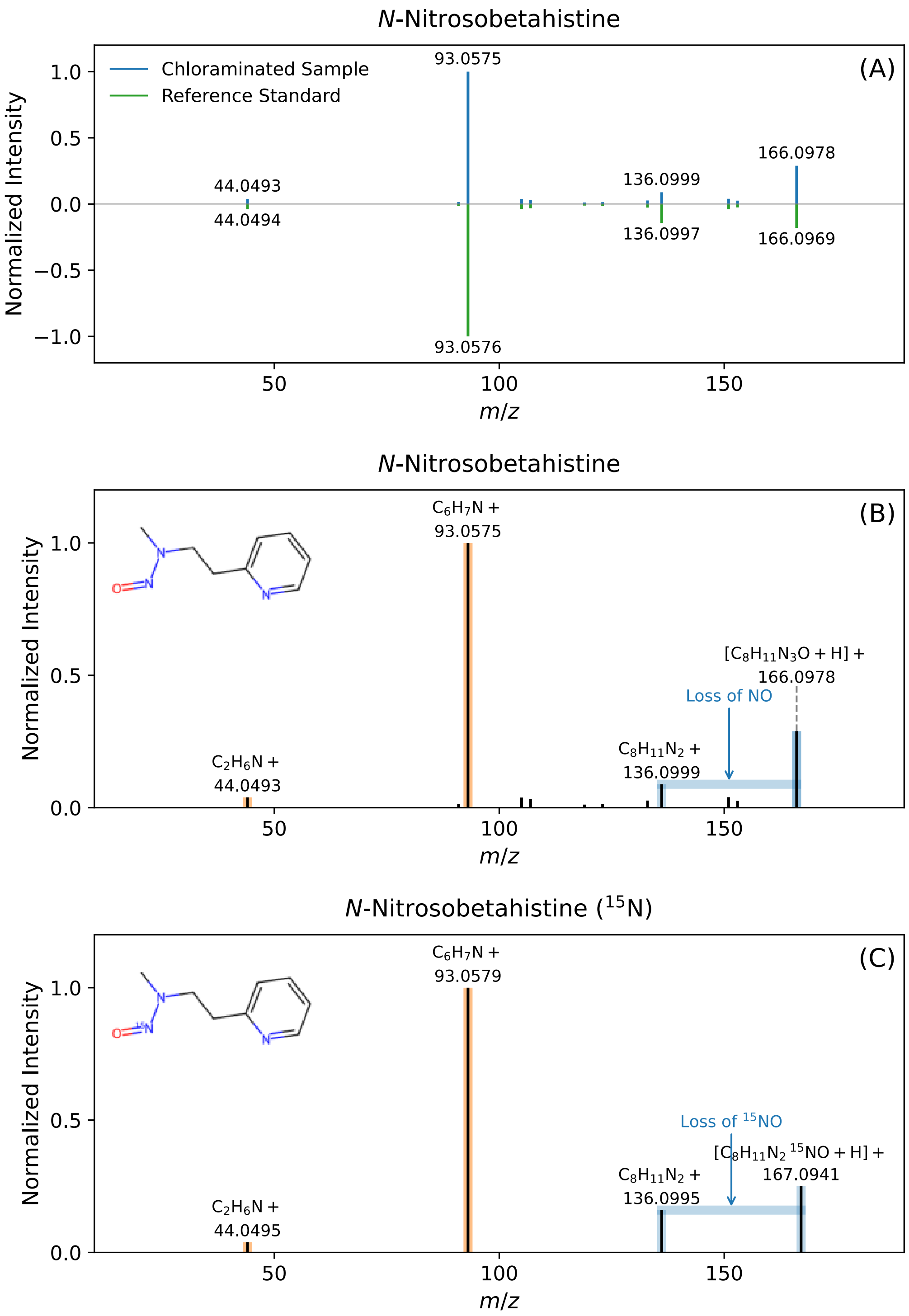

Figure S23. Confirmation of *N*-nitrosobetahistine formation upon chloramination of betahistine under formation potential test conditions: **(A)** Head-to-tail plots of the dd-MS2 spectra of *N*-nitrosobetahistine acquired from the chloraminated sample (top) and its reference standard (bottom). **(B)** Characteristic fragment ions highlighted in the dd-MS2 spectrum of *N*-nitrosobetahistine acquired from the chloraminated sample (Table S20). **(C)** Characteristic fragment ions highlighted in the dd-MS2 spectrum of *N*-nitrosobetahistine acquired from the ^{15}N chloraminated sample (Table S20).

**Table S21.** Confirmation of *N*-nitrosonebivolol formation by its reference standard and ^{15}N labeling							
Molecular Structure	Molecular Formula	Chloraminated Sample			Reference Standard		
		ΔMass [ppm]	*m/z*	RT [min]	ΔMass [ppm]	*m/z*	RT [min]
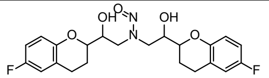	$[C_{22}H_{24}F_2N_2O_5+H]+$	-3.45	435.1711	18.50	-4.37	435.1707	18.50
Fragment Ion	Formula	ΔMass [ppm]	*m/z*	Fragment Ion	Formula	ΔMass [ppm]	*m/z*
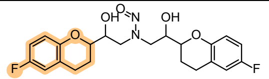	$[C_9H_8FO]+$	3.51	151.0559	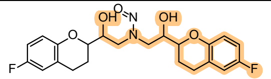	$[C_{13}H_{17}FNO_3]+$	1.97	254.1192
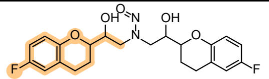	$[C_{11}H_{10}FO]+$	-1.81	177.0707	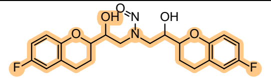	$[C_{22}H_{23}F_2NO_3]+$	2.32	387.1655
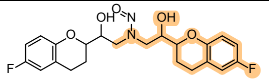	$[C_{12}H_{15}FNO_2]+$	4.33	224.1091	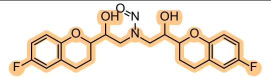	$[C_{22}H_{25}F_2NO_4]+$ (NO Loss)	2.32	405.1761
Molecular Structure	Molecular Formula	^{15}N Chloraminated Sample					
		ΔMass [ppm]	*m/z*	RT [min]			
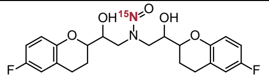	$[C_{22}H_{24}F_2N^{15}NO_5+H]+$	-1.93	436.1688	18.40			
Fragment Ion	Formula	ΔMass [ppm]	*m/z*	Fragment Ion	Formula	ΔMass [ppm]	*m/z*
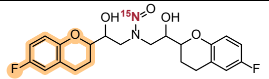	$[C_9H_8FO]+$	-0.46	151.0553	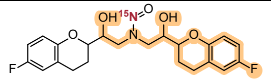	$[C_{13}H_{17}FNO_3]+$	0.39	254.1188
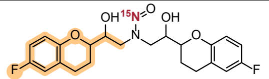	$[C_{11}H_{10}FO]+$	-0.11	177.0710	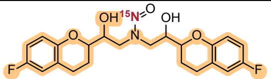	$[C_{22}H_{23}F_2NO_3]+$	-0.52	387.1644
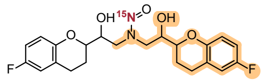	$[C_{12}H_{15}FNO_2]+$	-0.13	224.1081	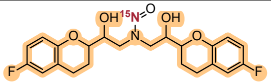	$[C_{22}H_{25}F_2NO_4]+$ (^{15}NO Loss)	-3.60	405.1737

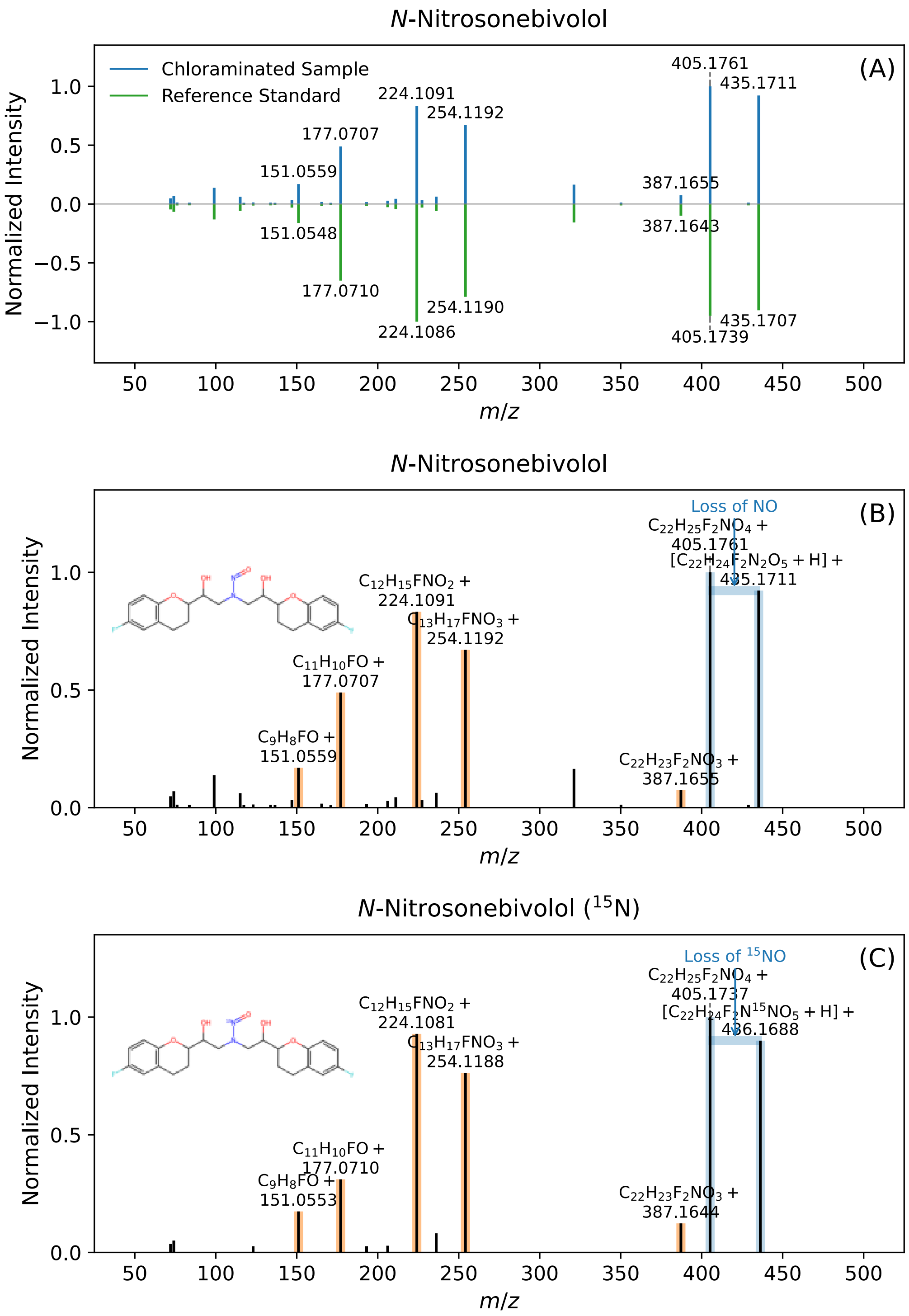

Figure S24. Confirmation of *N*-nitrosonebivolol formation upon chloramination of nebivolol under formation potential test conditions: **(A)** Head-to-tail plots of the dd-MS2 spectra of *N*-nitrosonebivolol acquired from the chloraminated sample (top) and its reference standard (bottom). **(B)** Characteristic fragment ions highlighted in the dd-MS2 spectrum of *N*-nitrosonebivolol acquired from the chloraminated sample (Table S21). **(C)** Characteristic fragment ions highlighted in the dd-MS2 spectrum of *N*-nitrosonebivolol acquired from the ^{15}N chloraminated sample (Table S21).

Table S22. Confirmation of *N*-nitrosometoprolol formation by its reference standard and ^{15}N labeling

Molecular Structure	Molecular Formula	Chloraminated Sample			Reference Standard		
		ΔMass [ppm]	*m/z*	RT [min]	ΔMass [ppm]	*m/z*	RT [min]
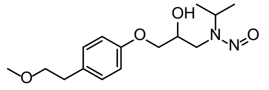	$[C_{15}H_{24}N_2O_4+H]+$	0.07	297.1809	16.90	1.08	297.1812	16.85
Fragment Ion	Formula	ΔMass [ppm]	*m/z*	Fragment Ion	Formula	ΔMass [ppm]	*m/z*
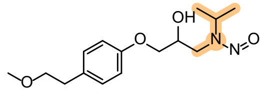	$[C_4H_{10}N]+$	-3.75	72.0805	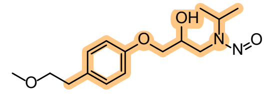	$[C_{13}H_{21}NO_2]+$	-4.17	223.1563
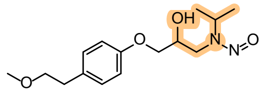	$[C_5H_{12}NO]+$	-1.37	102.0912	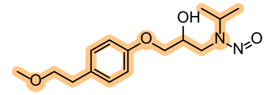	$[C_{15}H_{23}NO_2]+$	-4.73	249.1717
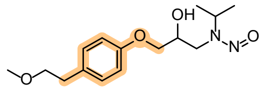	$[C_8H_9O]+$	-4.05	121.0643	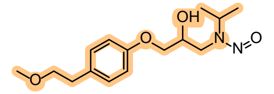	$[C_{15}H_{25}NO_3]+$ (NO Loss)	-3.52	267.1825
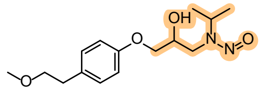	$[C_6H_{13}N_2O_2]+$	4.48	145.0978				
Molecular Structure	Molecular Formula	^{15}N Chloraminated Sample					
		ΔMass [ppm]	*m/z*	RT [min]			
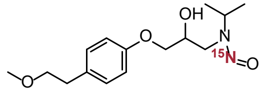	$[C_{15}H_{24}N^{15}NO_4+H]+$	-3.76	298.1768	16.98			
Fragment Ion	Formula	ΔMass [ppm]	*m/z*	Fragment Ion	Formula	ΔMass [ppm]	*m/z*
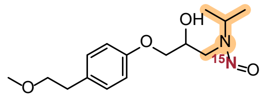	$[C_4H_{10}N]+$	1.80	72.0809	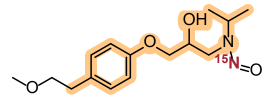	$[C_{13}H_{21}NO_2]+$	3.45	223.1580
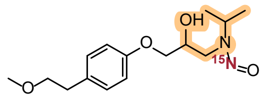	$[C_5H_{12}NO]+$	1.57	102.0915	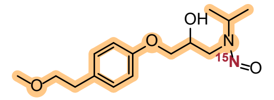	$[C_{15}H_{23}NO_2]+$	-1.93	249.1724
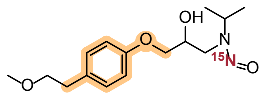	$[C_8H_9O]+$	-0.74	121.0647	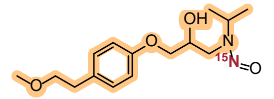	$[C_{15}H_{25}NO_3]+$ (^{15}NO Loss)	-3.14	267.1826
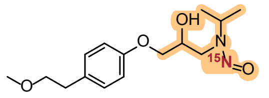	$[C_6H_{13}N^{15}NO_2]+$	-1.30	146.0940				

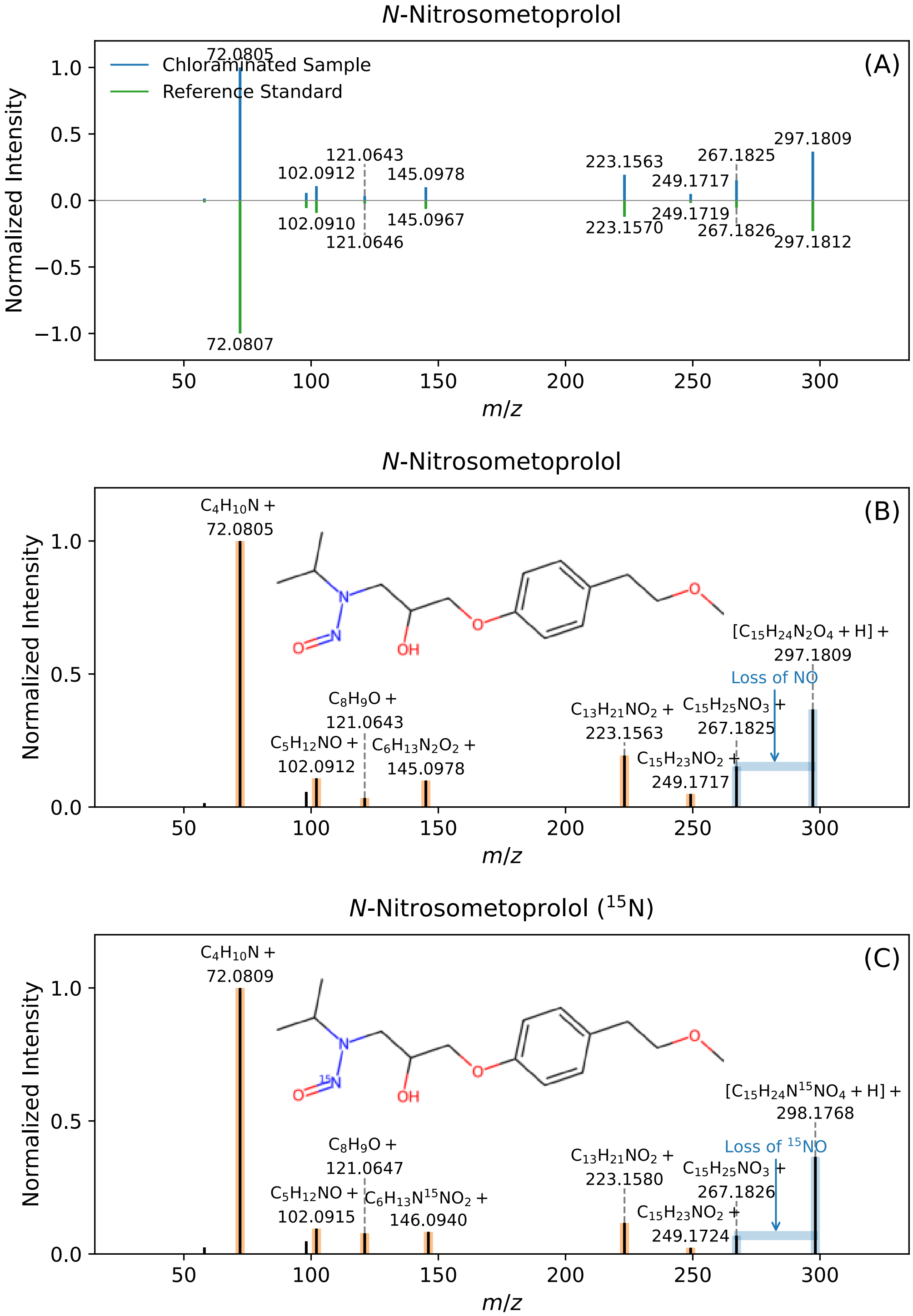

Figure S25. Confirmation of *N*-nitrosometoprolol formation upon chloramination of metoprolol under formation potential test conditions: **(A)** Head-to-tail plots of the dd-MS2 spectra of *N*-nitrosometoprolol acquired from the chloraminated sample (top) and its reference standard (bottom). **(B)** Characteristic fragment ions highlighted in the dd-MS2 spectrum of *N*-nitrosometoprolol acquired from the chloraminated sample (Table S22). **(C)** Characteristic fragment ions highlighted in the dd-MS2 spectrum of *N*-nitrosometoprolol acquired from the ^{15}N chloraminated sample (Table S22).

Table S23. Confirmation of *N*-nitrosodesipramine formation by its reference standard and ^{15}N labeling

Molecular Structure	Molecular Formula	Chloraminated Sample			Reference Standard		
		ΔMass [ppm]	*m/z*	RT [min]	ΔMass [ppm]	*m/z*	RT [min]
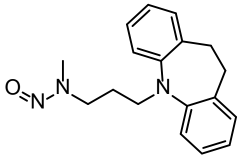	$[C_{18}H_{21}N_{3}O+H]+$	-3.82	296.1746	19.00	-0.14	296.1757	18.89
Fragment Ion	Formula	ΔMass [ppm]	*m/z*	Fragment Ion	Formula	ΔMass [ppm]	*m/z*
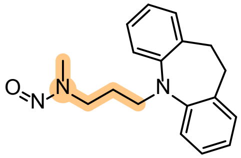	$[C_{4}H_{9}N]+$	-2.81	71.0733	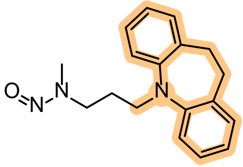	$[C_{15}H_{14}N]+$	-1.30	208.1118
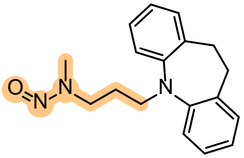	$[C_{4}H_{9}N_{2}O]+$	-1.39	101.0708	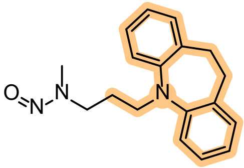	$[C_{16}H_{16}N]+$	-0.99	222.1275
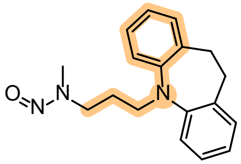	$[C_{9}H_{8}N]+$	-1.69	130.0649	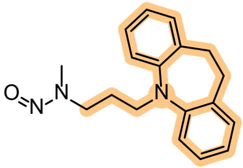	$[C_{17}H_{17}N]+$	-2.55	235.1355
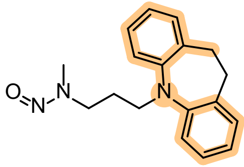	$[C_{13}H_{10}N]+$	-0.39	180.0807	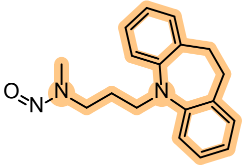	$[C_{18}H_{22}N_{2}]+$ (NO Loss)	-4.13	266.1772
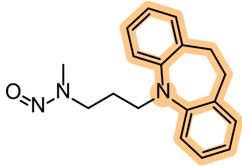	$[C_{14}H_{13}N]+$	-3.59	195.1041				

Table S23. Confirmation of *N*-nitrosodesipramine formation by its reference standard and ^{15}N labeling (continued)

Molecular Structure	Molecular Formula	^{15}N Chloraminated Sample					
		ΔMass [ppm]	*m/z*	RT [min]			
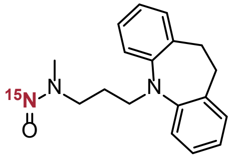	$[C_{18}H_{21}N_2{}^{15}NO+H]+$	-0.91	297.1725	18.84			
Fragment Ion	Formula	ΔMass [ppm]	*m/z*	Fragment Ion	Formula	ΔMass [ppm]	*m/z*
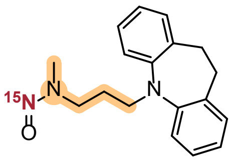	$[C_4H_9N]+$	-1.41	71.0734	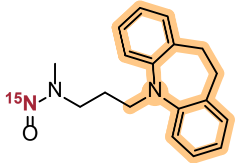	$[C_{15}H_{14}N]+$	1.11	208.1123
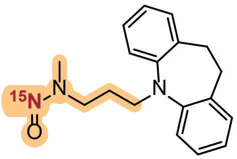	$[C_4H_9N{}^{15}NO]+$	-1.67	102.0678	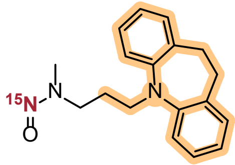	$[C_{16}H_{16}N]+$	-0.99	222.1275
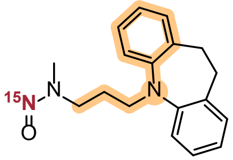	$[C_9H_8N]+$	-1.69	130.0649	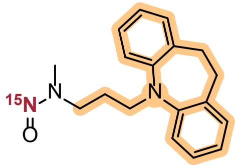	$[C_{17}H_{17}N]+$	-4.68	235.1350
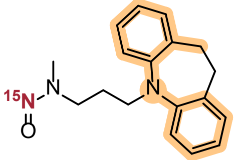	$[C_{13}H_{10}N]+$	-0.39	180.0807	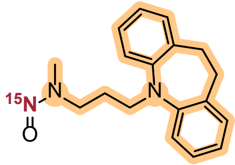	$[C_{18}H_{22}N_2]+$ (^{15}NO Loss)	-1.88	266.1778
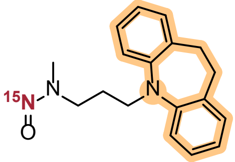	$[C_{14}H_{13}N]+$	-2.56	195.1043				

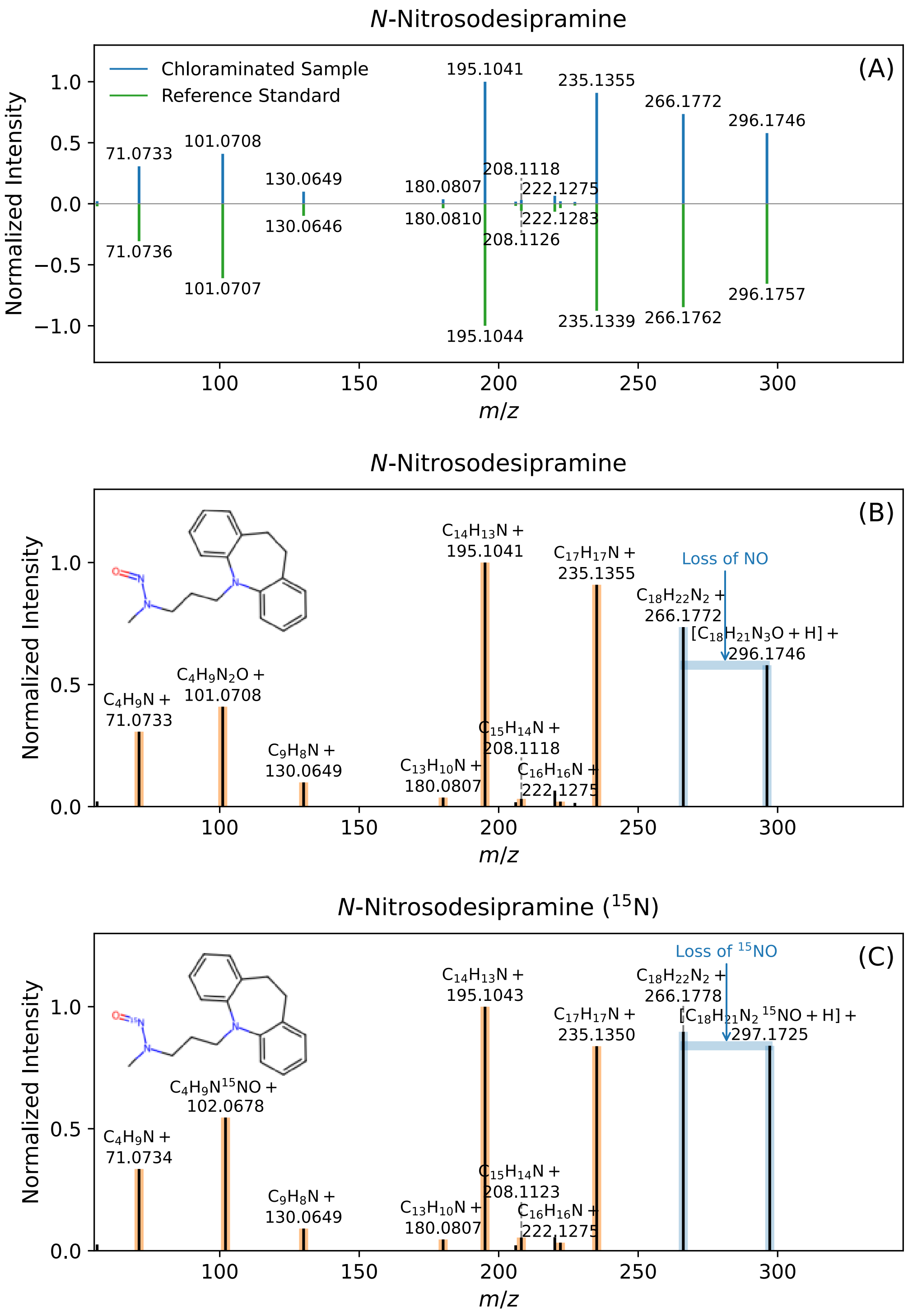

Figure S26. Confirmation of *N*-nitrosodesipramine formation upon chloramination of desipramine under formation potential test conditions: **(A)** Head-to-tail plots of the dd-MS2 spectra of *N*-nitrosodesipramine acquired from the chloraminated sample (top) and its reference standard (bottom). **(B)** Characteristic fragment ions highlighted in the dd-MS2 spectrum of *N*-nitrosodesipramine acquired from the chloraminated sample (Table S23). **(C)** Characteristic fragment ions highlighted in the dd-MS2 spectrum of *N*-nitrosodesipramine acquired from the ^{15}N chloraminated sample (Table S23).

Table S24. Confirmation of *N*-nitrosonortriptyline formation by its reference standard and ^{15}N labeling

Molecular Structure	Molecular Formula	Chloraminated Sample			Reference Standard		
		ΔMass [ppm]	*m/z*	RT [min]	ΔMass [ppm]	*m/z*	RT [min]
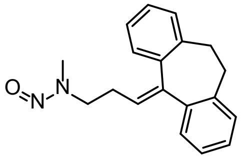	$[C_{19}H_{20}N_2O+H]+$	-4.57	293.1635	18.80	-3.21	293.1639	18.79
Fragment Ion	Formula	ΔMass [ppm]	*m/z*	Fragment Ion	Formula	ΔMass [ppm]	*m/z*
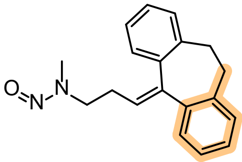	$[C_7H_7]+$	-4.72	91.0538	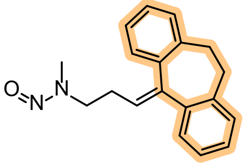	$[C_{15}H_{11}]+$	-2.25	191.0851
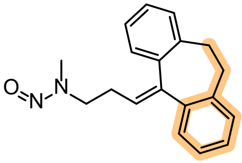	$[C_8H_9]+$	-2.66	105.0696	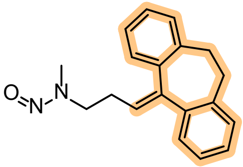	$[C_{16}H_{13}]+$	-1.85	205.1008
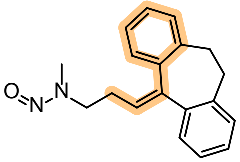	$[C_9H_9]+$	-2.39	117.0696	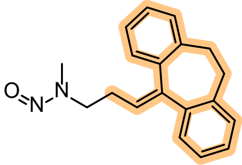	$[C_{17}H_{14}]+$	-4.36	218.1086
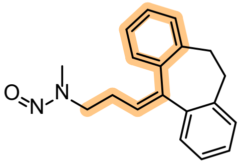	$[C_{10}H_9]+$	-4.49	129.0693	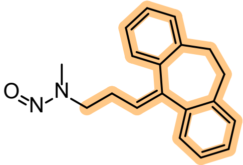	$[C_{18}H_{17}]+$	-2.06	233.1320
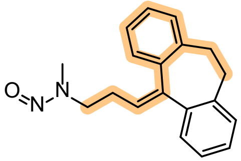	$[C_{12}H_{11}]+$	-2.13	155.0852	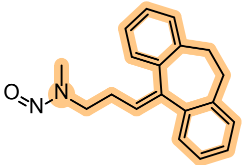	$[C_{19}H_{21}N]+$ (NO Loss)	-4.56	263.1662

**Table S24.** Confirmation of *N*-nitrosonortriptyline formation by its reference standard and ^{15}N labeling (continued)							
Molecular Structure	Molecular Formula	^{15}N Chloraminated Sample					
		ΔMass [ppm]	*m/z*	RT [min]			
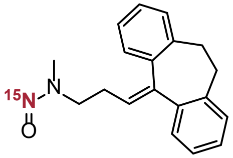	$[C_{19}H_{20}N^{15}NO+H]+$	-3.30	294.1609	18.67			
Fragment Ion	Formula	ΔMass [ppm]	*m/z*	Fragment Ion	Formula	ΔMass [ppm]	*m/z*
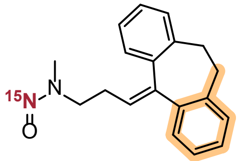	$[C_7H_7]+$	-0.33	91.0542	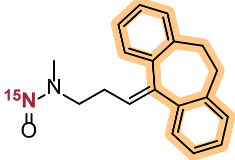	$[C_{15}H_{11}]+$	-0.68	191.0854
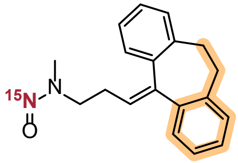	$[C_8H_9]+$	0.19	105.0699	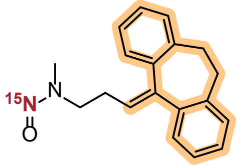	$[C_{16}H_{13}]+$	0.10	205.1012
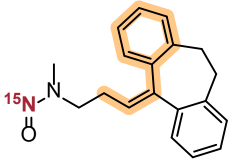	$[C_9H_9]+$	-2.39	117.0696	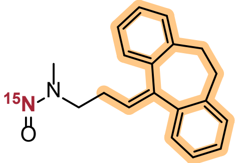	$[C_{17}H_{14}]+$	-2.52	218.1090
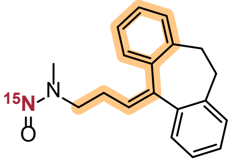	$[C_{10}H_9]+$	-2.17	129.0696	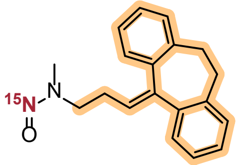	$[C_{18}H_{17}]+$	-1.63	233.1321
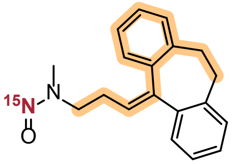	$[C_{12}H_{11}]+$	0.45	155.0856	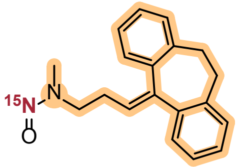	$[C_{19}H_{21}N]+$ (^{15}NO Loss)	-4.94	263.1661

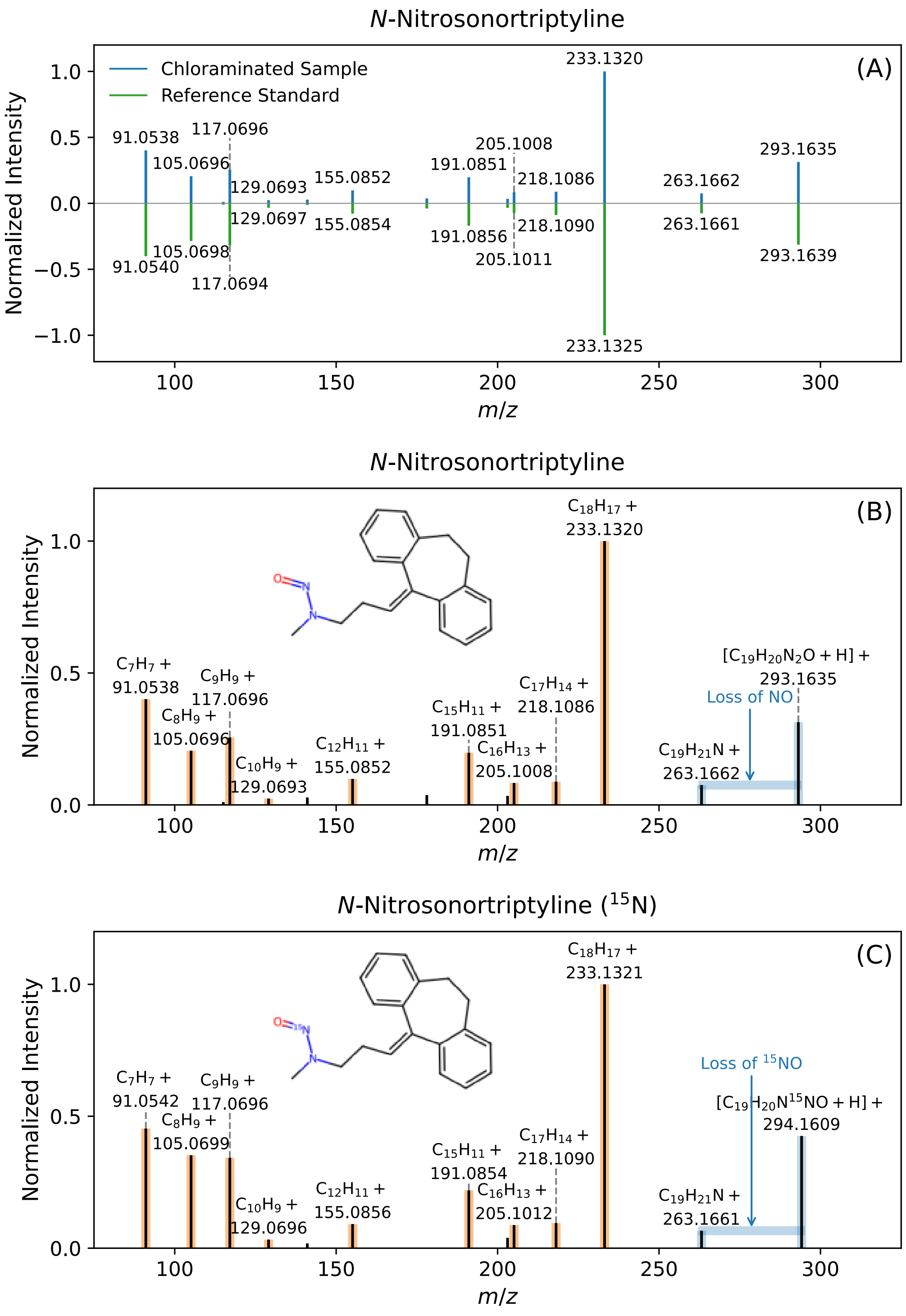

Figure S27. Confirmation of *N*-nitrosonortriptyline formation upon chloramination of nortriptyline under formation potential test conditions: **(A)** Head-to-tail plots of the dd-MS2 spectra of *N*-nitrosonortriptyline acquired from the chloraminated sample (top) and its reference standard (bottom). **(B)** Characteristic fragment ions highlighted in the dd-MS2 spectrum of *N*-nitrosonortriptyline acquired from the chloraminated sample (Table S24). **(C)** Characteristic fragment ions highlighted in the dd-MS2 spectrum of *N*-nitrosonortriptyline acquired from the ^{15}N chloraminated sample (Table S24).

Table S25. Confirmation of *N*-nitrosoiminodibenzyl formation by its reference standard and ^{15}N labeling

Molecular Structure	Molecular Formula	Chloraminated Sample			Reference Standard		
		ΔMass [ppm]	*m/z*	RT [min]	ΔMass [ppm]	*m/z*	RT [min]
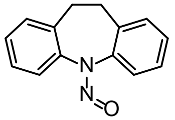	$[C_{14}H_{12}N_2O+H]+$	1.60	225.1026	18.00	-0.18	225.1022	17.96
Fragment Ion	Formula	ΔMass [ppm]	*m/z*	Fragment Ion	Formula	ΔMass [ppm]	*m/z*
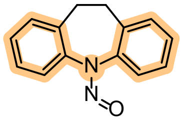	$[C_{12}H_{11}N]+$	-3.84	169.0885	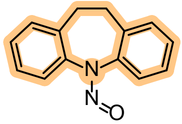	$[C_{14}H_{13}N]+$ (NO Loss)	-3.59	195.1041
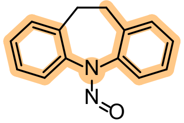	$[C_{13}H_{10}N]+$	-0.39	180.0807				
Molecular Structure	Molecular Formula	^{15}N Chloraminated Sample					
		ΔMass [ppm]	*m/z*	RT [min]			
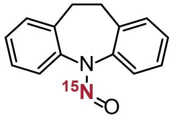	$[C_{14}H_{12}N^{15}NO+H]+$	-2.52	226.0987	17.87			
Fragment Ion	Formula	ΔMass [ppm]	*m/z*	Fragment Ion	Formula	ΔMass [ppm]	*m/z*
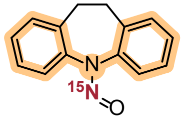	$[C_{12}H_{11}N]+$	-2.66	169.0887	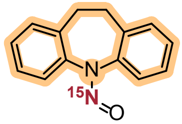	$[C_{14}H_{13}N]+$ (^{15}NO Loss)	-2.56	195.1043
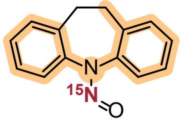	$[C_{13}H_{10}N]+$	-0.94	180.0806				

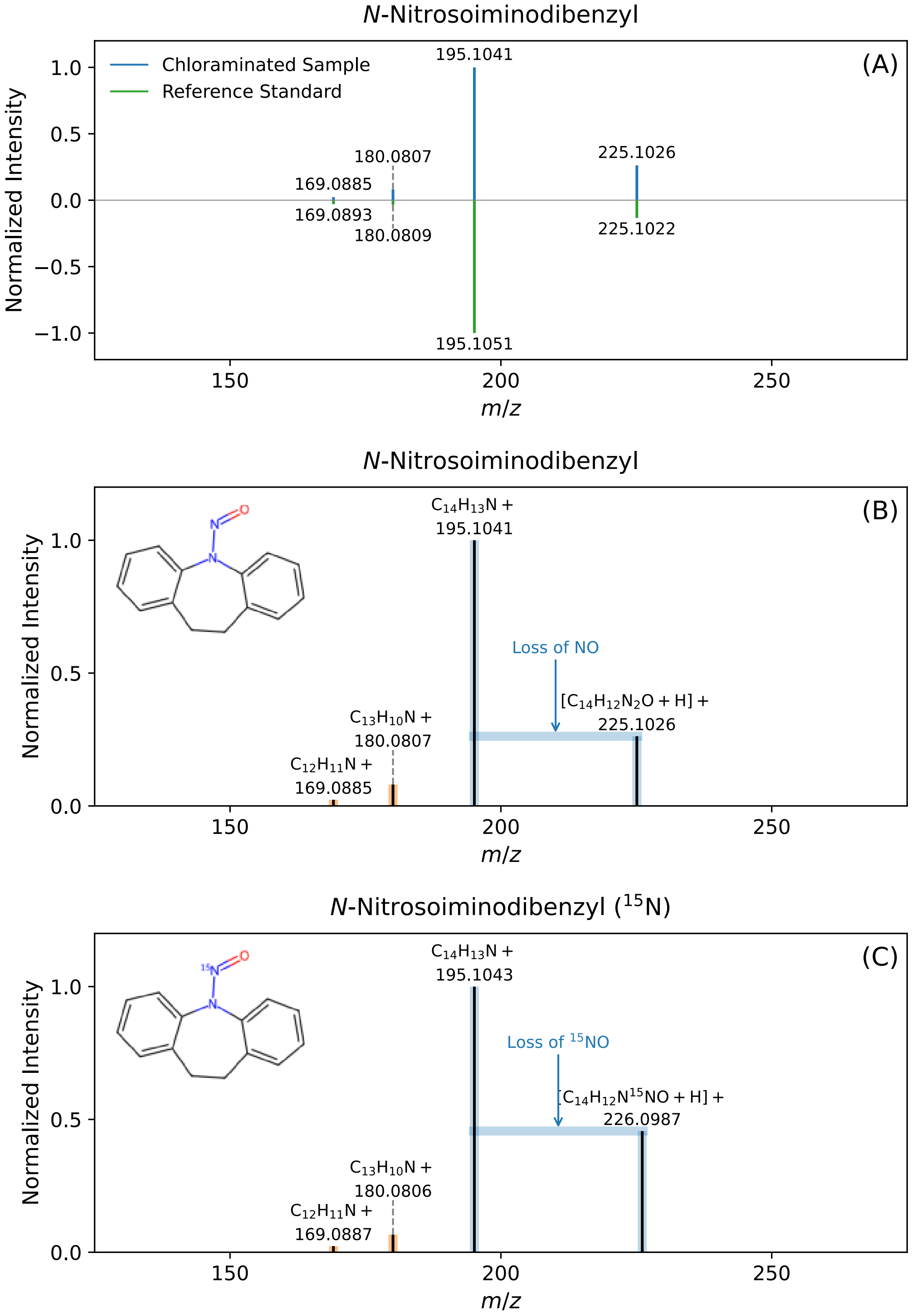

Figure S28. Confirmation of *N*-nitrosoiminodibenzyl formation upon chloramination of iminodibenzyl under formation potential test conditions: **(A)** Head-to-tail plots of the dd-MS2 spectra of *N*-nitrosoiminodibenzyl acquired from the chloraminated sample (top) and its reference standard (bottom). **(B)** Characteristic fragment ions highlighted in the dd-MS2 spectrum of *N*-nitrosoiminodibenzyl acquired from the chloraminated sample (Table S25). **(C)** Characteristic fragment ions highlighted in the dd-MS2 spectrum of *N*-nitrosoiminodibenzyl acquired from the ^{15}N chloraminated sample (Table S25).

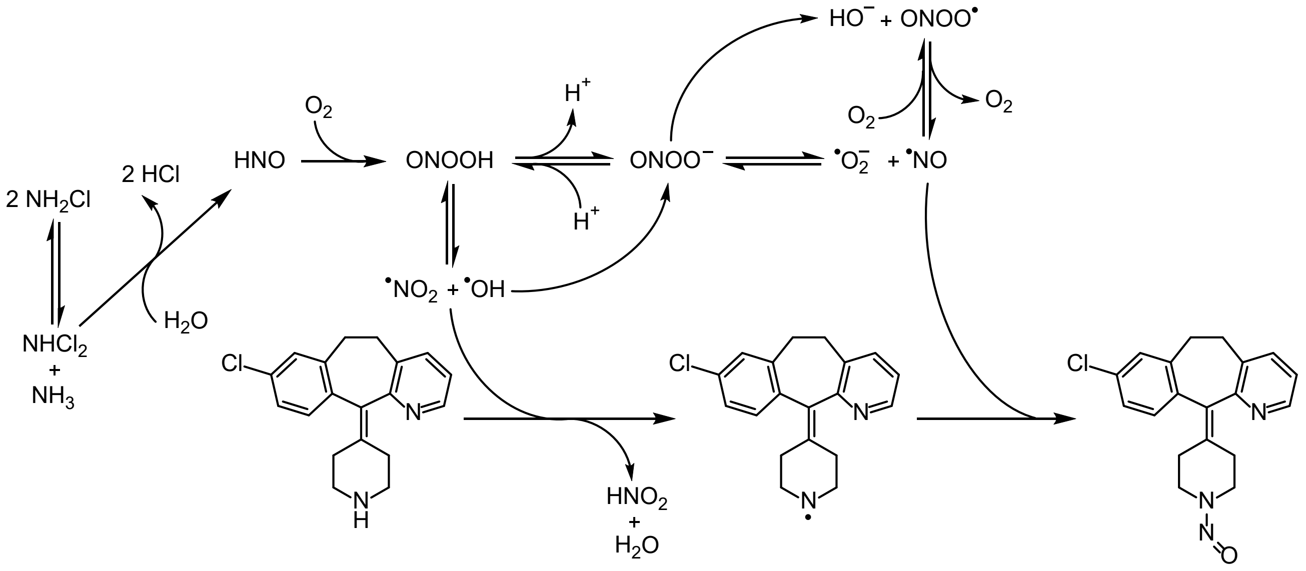

Figure S29. Proposed pathway for the formation of *N*-nitrosodesloratadine upon chloramination of desloratadine adapted after Pham *et al.* (2021),[8] Masuda *et al.* (2000),[9] Uppu *et al.* (2000),[10] Kirsch *et al.* (2003),[11] and Schreiber and Mitch (2007).[12] Only the reactions leading to *N*-nitrosodesloratadine formation are shown, and further mechanistic studies are required to identify intermediates and/or products of interest.

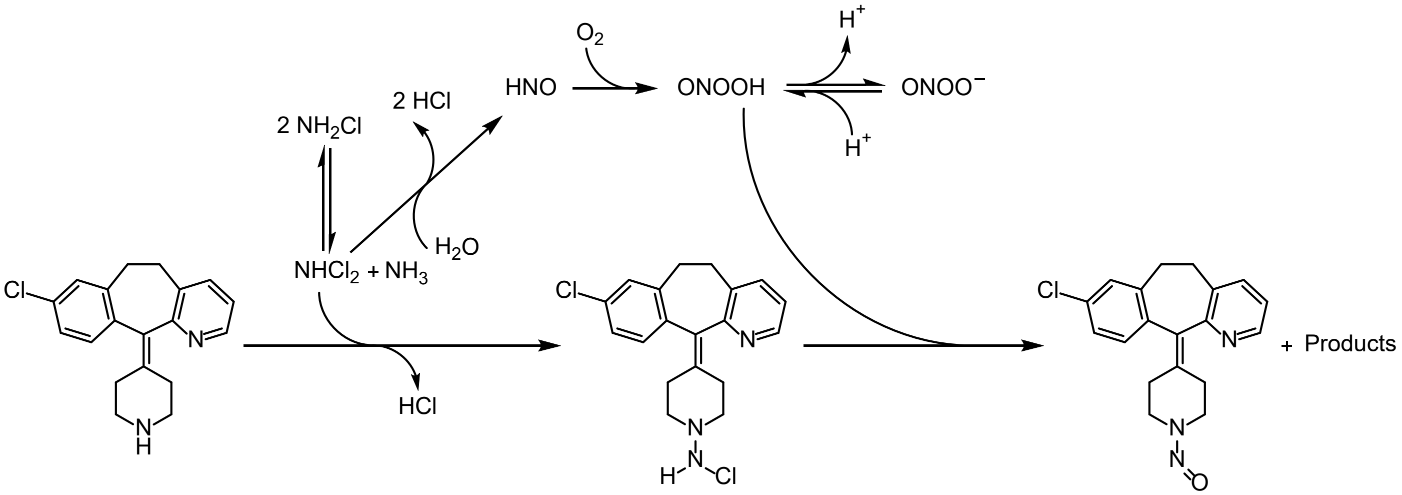

Figure S30. Proposed pathway for the formation of *N*-nitrosodesloratadine upon chloramination of desloratadine adapted after Schreiber and Mitch (2006)[13] and Pham *et al.* (2021).[8] Only the reactions leading to *N*-nitrosodesloratadine formation are shown, and further mechanistic studies are required to identify intermediates and/or products of interest.

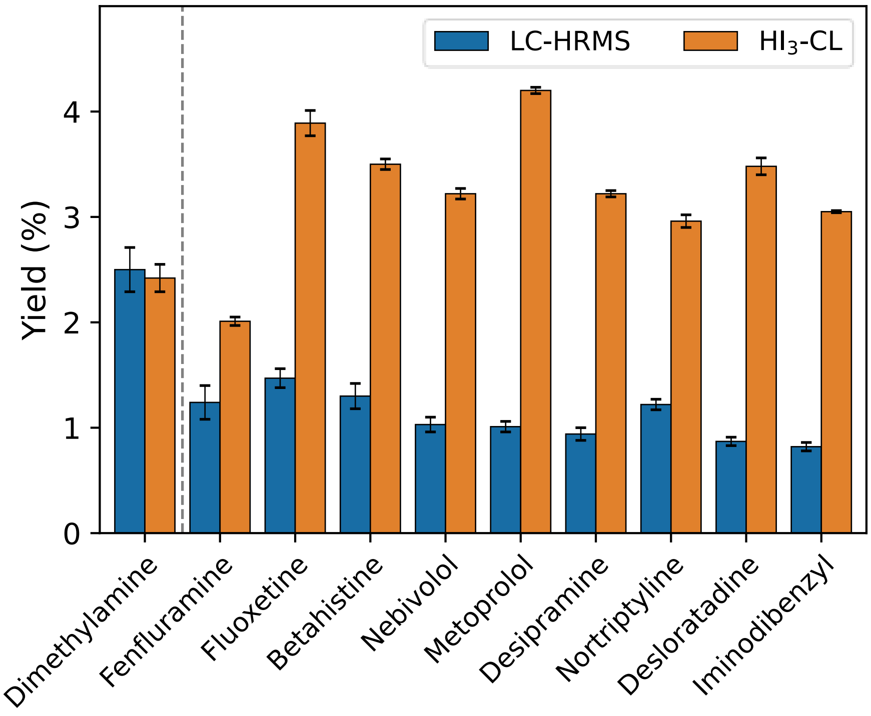

Figure S31. Formation of *N*-nitroso derivatives and other co-occurring *N*-nitrosamines from nine secondary amine-containing pharmaceuticals (10 µM) upon chloramination ($[NH_2Cl]_0$ = 2 mM at pH 6.9±0.2) measured by LC-HRMS and the acidic triiodide-chemiluminescence (HI_3-CL) method. NDMA yields from dimethylamine were determined for comparison with existing literature data.

12. Covariation of uncharacterized TONO formation potential with protein-like organic matter

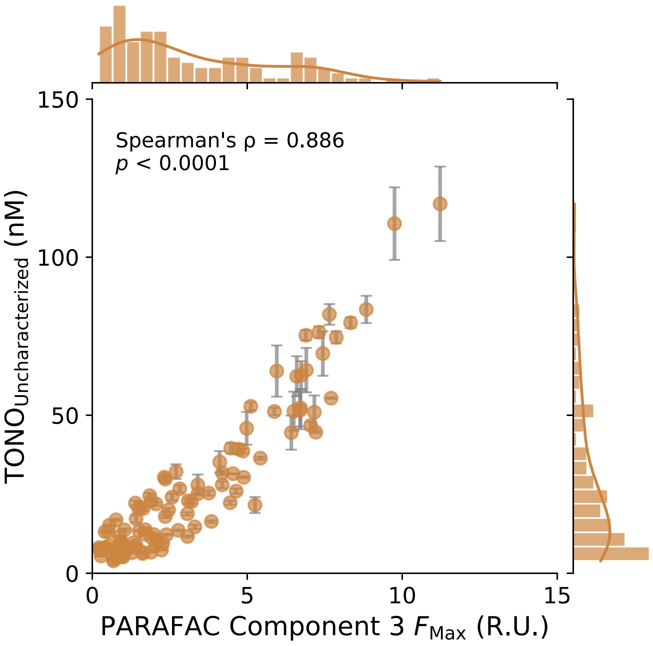

Figure S32. Spearman's correlation between uncharacterized TONO formation potential and the maximum fluorescence intensity of PARAFAC component 3 (a tryptophan-like component) in wastewater samples from WWTPs A-H. Error bars represent the standard deviations from duplicate measurements; where absent, bars fall within symbols.

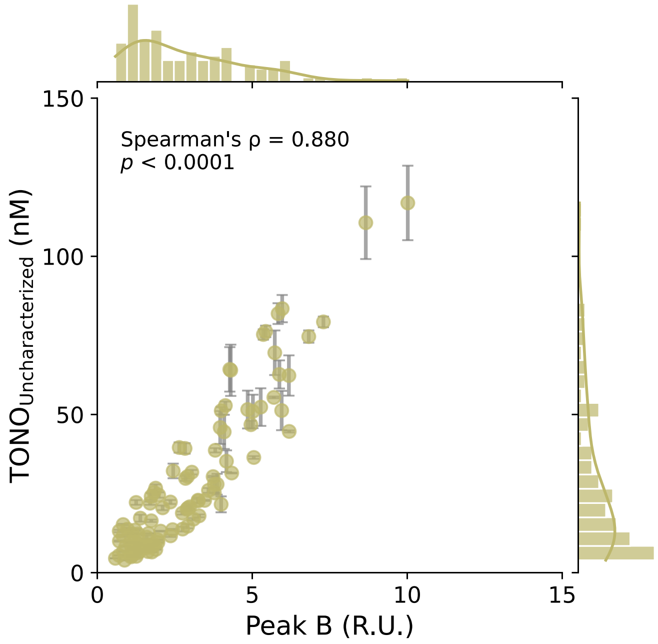

Figure S33. Spearman's correlation between uncharacterized TONO formation potential and the fluorescence intensity of Peak B (an indicator of the abundance of the tyrosine-like organic matter fraction) in wastewater samples from WWTPs A-H. Error bars represent the standard deviations from duplicate measurements; where absent, bars fall within symbols.

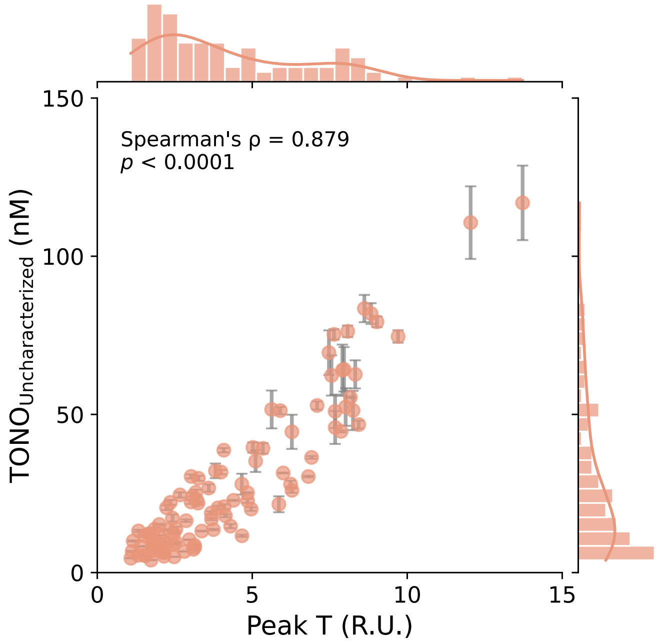

Figure S34. Spearman's correlation between uncharacterized TONO formation potential and the fluorescence intensity of Peak T (an indicator of the abundance of the tryptophan-like organic matter fraction) in wastewater samples from WWTPs A-H. Error bars represent the standard deviations from duplicate measurements; where absent, bars fall within symbols.

References

(1) Schreiber, I. M.; Mitch, W. A. Influence of the order of reagent addition on NDMA formation during chloramination. *Environmental Science & Technology* **2005**, *39* (10), 3811-3818.
(2) Murphy, K. R.; Stedmon, C. A.; Graeber, D.; Bro, R. Fluorescence spectroscopy and multi-way techniques. PARAFAC. *Analytical Methods* **2013**, *5* (23), 6557-6566.
(3) Murphy, K. R.; Timko, S. A.; Gonsior, M.; Powers, L. C.; Wünsch, U. J.; Stedmon, C. A. Photochemistry illuminates ubiquitous organic matter fluorescence spectra. *Environmental Science & Technology* **2018**, *52* (19), 11243-11250.
(4) Murphy, K. R.; Stedmon, C. A.; Wenig, P.; Bro, R. OpenFluor- an online spectral library of auto-fluorescence by organic compounds in the environment. *Analytical Methods* **2014**, *6* (3), 658-661.
(5) Lambert, T.; Bouillon, S.; Darchambeau, F.; Massicotte, P.; Borges, A. V. Shift in the chemical composition of dissolved organic matter in the Congo River network. *Biogeosciences* **2016**, *13* (18), 5405-5420.
(6) Fellman, J. B.; Hood, E.; Spencer, R. G. M. Fluorescence spectroscopy opens new windows into dissolved organic matter dynamics in freshwater ecosystems: A review. *Limnology and Oceanography* **2010**, *55* (6), 2452-2462.
(7) Murphy, K. R.; Hambly, A.; Singh, S.; Henderson, R. K.; Baker, A.; Stuetz, R.; Khan, S. J. Organic matter fluorescence in municipal water recycling schemes: Toward a unified PARAFAC model. *Environmental Science & Technology* **2011**, *45* (7), 2909-2916.
(8) Pham, H. T.; Wahman, D. G.; Fairey, J. L. Updated reaction pathway for dichloramine decomposition: Formation of reactive nitrogen species and *N*-nitrosodimethylamine. *Environmental Science & Technology* **2021**, *55* (3), 1740-1749.
(9) Masuda, M.; Mower, H. F.; Pignatelli, B.; Celan, I.; Friesen, M. D.; Nishino, H.; Ohshima, H. Formation of *N*-nitrosamines and *N*-nitramines by the reaction of secondary amines with peroxynitrite and other reactive nitrogen species: Comparison with nitrotyrosine formation. *Chemical Research in Toxicology* **2000**, *13* (4), 301-308.
(10) Uppu, R. M.; Squadrito, G. L.; Bolzan, R. M.; Pryor, W. A. Nitration and nitrosation by peroxynitrite: Role of CO_2 and evidence for common intermediates. *Journal of the American Chemical Society* **2000**, *122* (29), 6911-6916.
(11) Kirsch, M.; Korth, H.-G.; Wensing, A.; Sustmann, R.; de Groot, H. Product formation and kinetic simulations in the pH range 1–14 account for a free-radical mechanism of peroxynitrite decomposition. *Archives of Biochemistry and Biophysics* **2003**, *418* (2), 133-150.
(12) Schreiber, I. M.; Mitch, W. A. Enhanced nitrogenous disinfection byproduct formation near the breakpoint: Implications for nitrification control. *Environmental Science & Technology* **2007**, *41* (20), 7039-7046.
(13) Schreiber, I. M.; Mitch, W. A. Nitrosamine formation pathway revisited: The importance of chloramine speciation and dissolved oxygen. *Environmental Science & Technology* **2006**, *40* (19), 6007-6014.
